# Regional and national burden of six major digestive cancers in Asia, 1990–2023: An analysis of the GBD Study 2023

**DOI:** 10.1016/j.isci.2026.115728

**Published:** 2026-04-15

**Authors:** Liqun Zhang, Fang Li, Jingdong Zhang

**Affiliations:** 1 Department of Gastrointestinal Medical Oncology, Cancer Hospital of Dalian University of Technology, Liaoning Cancer Hospital & Institute, No.44 Xiaoheyan Road, Dadong District, Shenyang, Liaoning 110042, China; 2 School of Biomedical Engineering, Faculty of Medicine, Dalian University of Technology, No.2 Linggong Road, Ganjingzi District, Dalian, Liaoning 116024, China; 3 Department of Hepatobiliary Surgery, Cancer Hospital of Dalian University of Technology, Liaoning Cancer Hospital & Institute, No.44 Xiaoheyan Road, Dadong District, Shenyang, Liaoning 110042, China

**Keywords:** Health sciences, Medicine, Medical specialty, Oncology, Public health

## Abstract

Updated pan-Asian analyses capturing divergent digestive cancer trajectories through the COVID-19 era have been lacking. Using Global Burden of Disease 2023 data, we analyzed disability-adjusted life years (DALYs) for 6 digestive cancers across 34 Asian countries and territories from 1990 to 2023, with projections through 2040. Long-term declines in gastric cancer (GC) contrasted with rises in pancreatic cancer (PC) and colorectal cancer (CRC): GC average annual percent changes were −3.49% in East Asia and −3.77% in high-income Asia Pacific, whereas PC increased in South Asia (+1.53%) and Southeast Asia (+1.54%), and CRC rose in Southeast Asia (+1.30%). Notable pandemic-era shifts emerged: East Asian GC reversed from −6.94% in 2017–2020 to +2.04% in 2020–2023, while PC accelerated sharply, reaching +4.19% in East Asia (2020–2023) and +4.46% in South Asia (2015–2023). Absolute DALYs are projected to rise substantially by 2040 across nearly all regions, with South Asian esophageal cancer DALYs increasing from 3.44 to 8.86 million. Even in regions achieving age-standardized rate declines, demographic pressures will drive absolute burden upward.

## Highlights

• Pandemic reversed falling digestive cancer rates in high-income Asia
• Population aging and growth dominated absolute digestive cancer expansion
• Pancreatic and colorectal cancers escalated rapidly
• Absolute digestive cancer burdens will surge by 2040

## Graphical abstract

**Figure:**
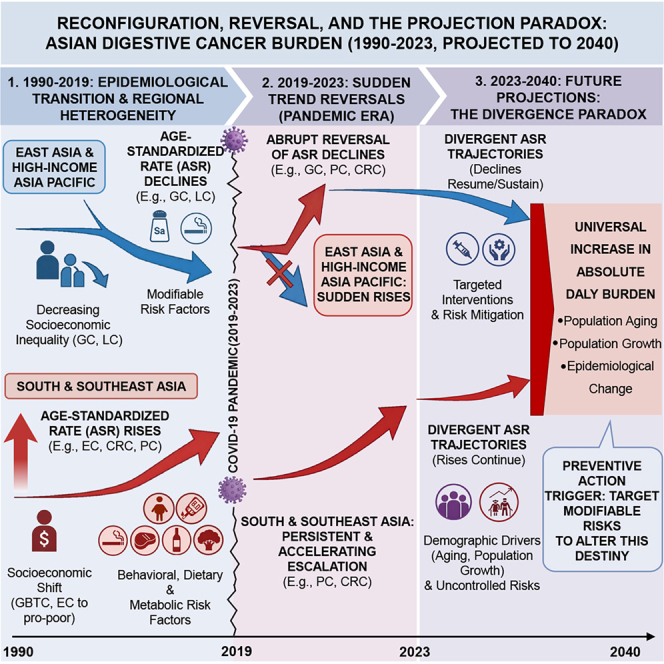

## Introduction

Global cancer mortality statistics indicate that digestive system malignancies impose a substantial burden on public health.^1^^,^^2^ Gastric (GC), liver (LC), gallbladder and biliary tract (GBTC), colorectal (CRC), pancreatic (PC), and esophageal (EC) cancers collectively constitute oncology’s most formidable challenge. Asia confronts this challenge with particular intensity.^3^ Home to more than half of humanity, the continent navigates a complex epidemiological transition where declining rates of infection-associated malignancies in economically advanced nations contrast with the rapid acceleration of lifestyle-driven cancers across diverse populations. High-income Asian countries have achieved notable reductions in GC and LC burden. Yet this progress masks an emerging crisis: PC and CRC are ascending rapidly, propelled by fundamental shifts in dietary patterns, metabolic profiles, and lifestyle behaviors.^4^^,^^5^^,^^6^^,^^7^

The continent’s socioeconomic heterogeneity generates profound analytical challenges.^8^^,^^9^ Although recent studies have provided valuable assessments of gastrointestinal cancer burdens in Asia using global burden of disease study (GBD) 2019 and 2021 data,^10^^,^^11^^,^^12^ an updated evaluation incorporating the latest GBD 2023 estimates is crucial. Prior analyses could not fully capture the divergent trajectories through the complete COVID-19 pandemic era (2019–2023). These disruptions may have substantially altered decades of cancer control momentum, yet their long-term impact remains inadequately quantified.^13^^,^^14^ Strategic resource allocation demands more than crude burden estimates; it requires parsing demographic forces (population growth and aging) from genuine epidemiological change—a systematic decomposition of drivers rarely executed at a continental scale.^15^ Without understanding how modifiable risk factors, socioeconomic disparities, and temporal dynamics intersect, public health interventions will remain inadequately targeted.^16^^,^^17^

To address these gaps, this study utilizes the GBD 2023 database to systematically analyze trends across 34 Asian countries and territories from 1990 to 2023 through four specific objectives.^17^ We primarily aimed to quantify the evolving burden of 6 major digestive cancers using disability-adjusted life years (DALYs)—corroborated by incidence and mortality patterns—and to detect significant temporal inflection points, with a specific focus on identifying trend reversals coincident with the COVID-19 pandemic era (2019–2023). Furthermore, we sought to decompose absolute burden changes into contributions driven by population growth, aging, and epidemiological shifts; evaluate socioeconomic inequalities in disease distribution using the slope index of inequality (SII) and concentration index (CIX); and project future burden and risk-attributable fractions through 2040 to inform targeted prevention strategies.

## Results

### Asian trends in digestive cancer burden

Global digestive cancer burden evolved with striking regional heterogeneity between 1990 and 2023, revealing catastrophic increases in several Asian subregions despite modest age-standardized rate (ASR) improvements elsewhere (Table 1). South Asia confronted the most alarming epidemiological trajectory. EC incidence surged 360.27% in absolute terms while age-standardized incidence rate (ASIR) climbed 74.72%; mortality escalated even more dramatically—up 405.90%—with age-standardized mortality rate (ASMR) rising 91.02%. DALYs increased 364.80%, accompanied by an 81.84% elevation in age-standardized DALY rate (ASDR). GBTC burden also intensified substantially: incident cases rose 277.52% (ASIR +36.66%), while mortality surged 313.03% (ASMR +47.10%).

**Table 1:** Temporal trends in the burden of six major digestive system cancers from 1990 to 2023: Asian estimates of incidence, prevalence, mortality, and DALYs

Measure	Location	EC			GC			LC			PC			GBTC			CRC		
		Number in thousands (ASR, per 100,000)		Change of all ages number (ASR) (%)	Number in thousands (ASR, per 100,000)		Change of all ages number (ASR) (%)	Number in thousands (ASR, per 100,000)		Change of all ages number (ASR) (%)	Number in thousands (ASR, per 100,000)		Change of all ages number (ASR) (%)	Number in thousands (ASR, per 100,000)		Change of all ages number (ASR) (%)	Number in thousands (ASR, per 100,000)		Change of all ages number (ASR) (%)
		1990	2023		1990	2023		1990	2023		1990	2023		1990	2023		1990	2023	
DALYs	Global	10275.61 (247.74)	14073.19 (152.93)	36.96 (−38.27)	24715.47 (592.53)	22528.49 (246.7)	−8.85 (−58.37)	7892.14 (179.8)	13931.34 (154.82)	76.52 (−13.89)	5433.33 (134.15)	12268.53 (133.3)	125.8 (−0.63)	2392.19 (59.88)	4036.21 (44.04)	68.72 (−26.46)	14839.51 (364.64)	26155.84 (287.36)	76.26 (−21.19)
DALYs	Central Asia	167.98 (351.14)	100.48 (106.6)	−40.19 (−69.64)	362.01 (731.87)	286.7 (299.11)	−20.8 (−59.13)	114.17 (222.39)	160.25 (167.56)	40.35 (−24.66)	46.16 (94.88)	99.82 (105.69)	116.24 (11.39)	13.63 (28.94)	15.47 (16.68)	13.44 (−42.36)	142.48 (288.14)	179.05 (191.23)	25.67 (−33.63)
DALYs	South Asia	739.34 (114.81)	3436.47 (208.76)	364.8 (81.84)	1548.46 (233.61)	3112.04 (185.74)	100.98 (−20.49)	407 (58.65)	1410.4 (83.26)	246.53 (41.97)	189.4 (30.58)	814.83 (49.95)	330.22 (63.34)	288.49 (47.42)	1114.49 (69.1)	286.32 (45.72)	765.08 (115.59)	2463.88 (147.94)	222.04 (27.98)
DALYs	East Asia	6483.02 (692.82)	5896.09 (243.29)	−9.05 (−64.88)	12355.48 (1290)	9139.07 (387.16)	−26.03 (−69.99)	3173.62 (308.81)	4222.51 (191.01)	33.05 (−38.15)	1268.55 (131.76)	2896.65 (122.19)	128.34 (−7.26)	513.72 (55.6)	788.43 (33.18)	53.48 (−40.32)	4229.71 (430.73)	6647.16 (288.99)	57.15 (−32.91)
DALYs	Southeast Asia	221.88 (77.9)	595.76 (76.79)	168.5 (−1.43)	768.54 (264.21)	1481.99 (193.37)	92.83 (−26.81)	607.32 (199.04)	1534.93 (198.58)	152.74 (−0.23)	164.41 (58.48)	735.72 (96.29)	347.51 (64.67)	124.14 (46.61)	347.62 (46.97)	180.03 (0.78)	661.1 (228.3)	2621.32 (344.35)	296.51 (50.83)
DALYs	High-income Asia Pacific	267.52 (129.58)	328.15 (74.38)	22.66 (−42.6)	1890.71 (933.81)	1220.26 (267.68)	−35.46 (−71.33)	806.68 (391.08)	803.31 (187.47)	−0.42 (−52.06)	420.59 (206.52)	1014.64 (223.17)	141.24 (8.06)	340.82 (169.41)	459.97 (89.67)	34.96 (−47.07)	918.09 (455.06)	1586.06 (353.44)	72.76 (−22.33)
Incidence	Global	395.28 (9.8)	605.29 (6.58)	53.13 (−32.82)	1051.32 (26.32)	1261.97 (13.81)	20.04 (−47.51)	271.57 (6.49)	569.96 (6.27)	109.88 (−3.37)	226.92 (5.91)	573.57 (6.28)	152.76 (6.32)	111.42 (2.95)	226.62 (2.5)	103.38 (−15.35)	944.52 (24.6)	2291.95 (25.11)	142.66 (2.08)
Incidence	Central Asia	6.3 (13.84)	3.85 (4.31)	−38.83 (−68.83)	12.81 (27.26)	10.84 (11.91)	−15.37 (−56.32)	3.83 (8.04)	5.84 (6.43)	52.41 (−19.99)	1.69 (3.65)	3.82 (4.3)	126.45 (17.83)	0.52 (1.16)	0.61 (0.7)	18.28 (−39.3)	6.19 (13.16)	9.54 (10.65)	54.12 (−19.07)
Incidence	South Asia	25.86 (4.36)	119 (7.61)	360.27 (74.72)	52.93 (8.72)	106.83 (6.69)	101.83 (−23.32)	13.21 (2.11)	46.21 (2.84)	249.73 (34.49)	6.94 (1.22)	29.16 (1.88)	320 (53.83)	10.62 (1.91)	40.09 (2.61)	277.52 (36.66)	28.65 (4.75)	104.86 (6.61)	266.04 (39.07)
Incidence	East Asia	245.35 (27.47)	271.95 (11.27)	10.84 (−58.98)	480.28 (53.12)	583 (24.54)	21.39 (−53.79)	101.82 (10.47)	170.54 (7.51)	67.5 (−28.32)	45.39 (5.03)	122.47 (5.14)	169.79 (2.22)	20.2 (2.34)	47.37 (2)	134.47 (−14.55)	192.86 (21.36)	640.6 (27)	232.17 (26.36)
Incidence	Southeast Asia	7.93 (2.98)	21.66 (2.9)	173.28 (−2.59)	27.21 (10.18)	56.46 (7.67)	107.49 (−24.65)	19.89 (7.16)	53.09 (7.1)	166.91 (−0.8)	5.93 (2.28)	26.61 (3.64)	348.82 (59.7)	4.88 (1.97)	15.09 (2.15)	209.12 (9.11)	27.77 (10.42)	137.37 (18.62)	394.69 (78.8)
Incidence	High-income Asia Pacific	13.7 (6.76)	26.55 (5.47)	93.8 (−19.07)	130.36 (65.03)	132.78 (25.94)	1.85 (−60.12)	34.85 (17.06)	58.81 (11.87)	68.75 (−30.42)	19.45 (9.82)	67.63 (12.54)	247.68 (27.63)	18.33 (9.41)	42.38 (7.12)	131.15 (−24.33)	81.15 (40.62)	236.58 (48.83)	191.53 (20.2)
Prevalence	Global	642.5 (15.47)	1076.27 (11.69)	67.51 (−24.46)	1793.98 (43.42)	2738.67 (29.85)	52.66 (−31.25)	348.3 (8.15)	837.17 (9.27)	140.36 (13.81)	201.08 (5.1)	550.08 (6.05)	173.56 (18.71)	135.76 (3.5)	325.37 (3.57)	139.66 (1.98)	4387.27 (111.1)	12274.04 (133.69)	179.76 (20.33)
Prevalence	Central Asia	9.67 (20.2)	6.05 (6.4)	−37.42 (−68.31)	18.11 (36.87)	15.94 (16.71)	−11.99 (−54.67)	4.33 (8.79)	6.65 (7.06)	53.86 (−19.59)	1.45 (3.02)	3.28 (3.53)	125.21 (16.8)	0.55 (1.19)	0.67 (0.74)	20.84 (−38.29)	22.91 (46.64)	40.41 (42.91)	76.37 (−8)
Prevalence	South Asia	41.71 (6.47)	194.64 (11.71)	366.59 (81.19)	75.57 (11.54)	158 (9.46)	109.07 (−18.01)	15.38 (2.34)	55.46 (3.31)	260.7 (41.6)	5.91 (0.97)	25.14 (1.56)	325.72 (60.03)	11.38 (1.93)	43.81 (2.76)	285.01 (42.96)	94.94 (14.62)	403.76 (24.21)	325.25 (65.64)
Prevalence	East Asia	386.33 (40.97)	466.28 (19.38)	20.69 (−52.69)	748.69 (78.02)	1389.17 (58.72)	85.55 (−24.74)	127.26 (12.62)	248.37 (11.44)	95.16 (−9.28)	39.62 (4.18)	107.49 (4.57)	171.27 (9.19)	22.87 (2.52)	71.28 (3.02)	211.67 (19.91)	796.39 (83.87)	3569.39 (149.13)	348.19 (77.8)
Prevalence	Southeast Asia	13.11 (4.58)	38.59 (4.99)	194.44 (8.98)	39.87 (13.82)	94.14 (12.32)	136.14 (−10.84)	23.58 (8.06)	66.68 (8.71)	182.82 (8.1)	5.16 (1.86)	23.7 (3.14)	358.89 (68.54)	5.43 (2.09)	18.54 (2.56)	241.6 (22.7)	103.9 (36.4)	617.07 (80.88)	493.89 (122.22)
Prevalence	High-income Asia Pacific	32.3 (15.68)	77.58 (17.43)	140.19 (11.19)	369.12 (180.34)	369.7 (81.27)	0.16 (−54.93)	52.82 (25.87)	113.44 (24.46)	114.75 (−5.45)	18.2 (9.1)	81.43 (15.02)	347.34 (65.03)	22.77 (11.52)	63.1 (11.1)	177.11 (−3.66)	460.51 (226.79)	1400.35 (308.84)	204.09 (36.18)
Mortality	Global	381.86 (9.62)	577.44 (6.3)	51.22 (−34.55)	912.48 (23.34)	934.8 (10.27)	2.45 (−56)	248.55 (6.05)	508.1 (5.59)	104.43 (−7.62)	220.26 (5.84)	551.68 (6.05)	150.47 (3.56)	101.27 (2.73)	185.15 (2.04)	82.82 (−25.24)	581.78 (15.69)	1106.74 (12.24)	90.23 (−22)
Mortality	Central Asia	6.25 (14.11)	3.79 (4.37)	−39.32 (−69)	12.34 (26.96)	10.25 (11.58)	−16.92 (−57.06)	3.73 (8.05)	5.69 (6.46)	52.7 (−19.75)	1.65 (3.66)	3.75 (4.33)	126.66 (18.17)	0.52 (1.2)	0.61 (0.72)	15.92 (−40.25)	4.87 (10.77)	6.56 (7.7)	34.72 (−28.47)
Mortality	South Asia	24.74 (4.36)	125.17 (8.34)	405.9 (91.02)	49.88 (8.62)	108.64 (7.03)	117.8 (−18.44)	12.48 (2.08)	47.38 (2.99)	279.55 (43.92)	6.73 (1.23)	30.81 (2.04)	357.73 (65.48)	10.45 (1.98)	43.16 (2.91)	313.03 (47.1)	24.49 (4.3)	85.7 (5.68)	249.88 (31.93)
Mortality	East Asia	240.76 (27.8)	259.86 (10.81)	7.93 (−61.11)	435.81 (50.07)	390.37 (16.46)	−10.43 (−67.14)	94.1 (9.96)	149.71 (6.49)	59.1 (−34.83)	44.2 (5.05)	120.41 (5.04)	172.42 (−0.12)	19.33 (2.34)	35.49 (1.5)	83.58 (−35.86)	138.95 (16.23)	273.57 (11.75)	96.88 (−27.62)
Mortality	Southeast Asia	7.48 (2.92)	20.44 (2.79)	173.24 (−4.59)	25.41 (9.95)	50.56 (7.03)	99.01 (−29.36)	18.72 (7.01)	50.3 (6.84)	168.71 (−2.4)	5.71 (2.29)	26.23 (3.66)	358.97 (59.88)	4.68 (1.96)	13.71 (2)	193.05 (1.73)	21.9 (8.75)	90.22 (12.68)	312.01 (44.98)
Mortality	High-income Asia Pacific	10.68 (5.33)	17.85 (3.47)	67.1 (−34.95)	74.77 (38.15)	74.53 (13.13)	−0.32 (−65.58)	29.26 (14.39)	45.26 (8.63)	54.69 (−40.03)	18.12 (9.21)	59.81 (11)	230.08 (19.47)	16.07 (8.33)	31.11 (5.08)	93.51 (−38.98)	37.27 (19.28)	90.34 (16.12)	142.39 (−16.39)

ASR, age-standardized rate; DALYs, disability-adjusted life years.

Southeast Asia experienced equally profound deterioration across multiple malignancies. CRC incidence increased 394.69%, with ASIR climbing 78.80%. PC cases surged 348.82% alongside a 59.70% ASIR increase, while LC and GBTC incidence rose 166.91% and 209.12%, respectively. CRC-related DALYs nearly quadrupled (296.51% increase), driving ASDR upward by 50.83%—a mounting public health crisis. East Asia presented a paradoxical picture: despite substantial ASIR declines for several cancers, absolute burden remained concerning. LC incidence increased 67.50% even as ASIR decreased 28.32%. PC cases rose even more dramatically—by 169.79%—driven predominantly by demographic transformation, as ASIR increased only marginally (+2.22%).

Contrasting sharply with these trends, Central Asia and high-income Asia Pacific achieved favorable trajectories in ASRs for multiple cancers. Central Asia reduced EC ASDR by 69.64% and ASMR by 69.00%, while GC ASDR decreased 59.13%. High-income Asia Pacific experienced comparable declines: GC ASMR fell 65.58% and ASDR dropped 71.33%, with marked reductions in EC ASDR (42.60%) and LC ASDR (52.06%). It is critical to note that while ASRs reflect shifts in epidemiological risk, absolute case counts frequently continued to rise in high-income regions, a trend predominantly driven by population growth and aging dynamics.

### National-level variation in ASRs (2023)

**Figure 1:**
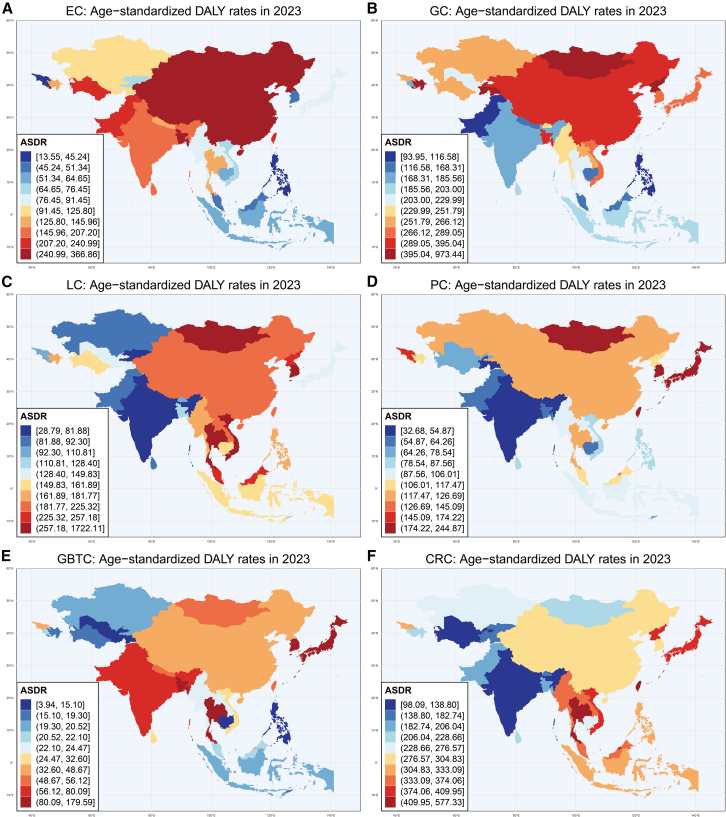
Geographical variation in ASDR for six major digestive system cancers by country and territory, 2023 (A) Esophageal cancer (EC). (B) Gastric cancer (GC). (C) Liver cancer (LC). (D) Pancreatic cancer (PC). (E) Gallbladder and biliary tract cancer (GBTC). (F) Colorectal cancer (CRC). Rates are shown per 100,000 population. Abbreviations: ASDR, age-standardized disability-adjusted life year (DALY) rates.

Geographic disparities in 2023 cancer burden revealed country-specific heterogeneity when examining ASDRs (Figure 1). Mongolia bore the most severe burden across multiple digestive malignancies: LC ASDR reached 1722.11 per 100,000, GC 973.44, and EC 366.86. For PC, Mongolia ranked second among all Asian nations (ASDR: 223.21), trailing only Japan, which recorded the highest burden (ASDR: 244.87). Bangladesh recorded the second-highest EC ASDR (283.51) and third-highest GBTC burden (96.23), indicating substantial population-level disability. The Democratic People’s Republic of Korea exhibited the third-highest ASDRs for both EC (257.61) and GC (480.73), while Tajikistan ranked second in GC ASDR (487.91). Economic advancement did not guarantee protection from high burden. Taiwan (Province of China) recorded the highest CRC ASDR (577.33) and third-highest PC ASDR (198.16) among all Asian nations and territories. Thailand demonstrated the second-highest LC ASDR (312.41) alongside the region’s peak GBTC ASDR (179.59). The Republic of Korea ranked third for LC ASDR (307.44) and second for GBTC ASDR (115.70), illustrating that high-income Asian nations carry substantial burdens of lifestyle- and metabolism-related digestive cancers despite their economic status.

The ASIRs largely paralleled ASDR distributions. Mongolia again demonstrated maximum EC incidence (16.02), GC (38.78), and LC (70.09). China ranked second for EC ASIR (11.31), reflecting persistent high incidence despite population-level interventions. Japan exhibited the highest PC ASIR (13.58), while Taiwan (Province of China) recorded the highest CRC ASIR (62.54) among all Asian countries and territories, followed by Japan (53.78). Figures S1–S3 present comprehensive ASIR, age-standardized prevalence rate (ASPR), and ASMR results.

### Age-specific patterns and trends (2023)

DALY rates for all six digestive cancers exhibited profound age dependency, escalating sharply in older populations (Figures S4–S27). The age of rapid burden escalation differed by malignancy: LC burden began accelerating earlier (around ages 40–44), whereas most other digestive cancers, including GC and PC, demonstrated steeper increases beginning at ages 50–59. Burden in younger age groups remained minimal across all cancers. Male predominance characterized DALY distributions for EC, GC, LC, PC, and CRC across most Asian regions. GBTC diverged from this pattern, exhibiting female predominance particularly pronounced in high-risk areas like South Asia. These age, sex, and geographic patterns held for incidence, prevalence, and mortality (Figures S4–S27).

### Distribution of absolute burden by age, sex, and region (2023)

Largely reflecting its massive population size, China shouldered the overwhelming majority of absolute DALY burden in 2023 across all 6 malignancies. India, similarly reflecting its demographic magnitude, contributed substantially to GBTC burden, while Japan, India, and the Republic of Korea bore considerable CRC burden alongside China. These absolute rankings primarily reflect population scale; comparative epidemiological risk is better assessed through ASRs (Figure 1). Age concentration patterns remained consistent: burden escalated sharply beyond ages 50 for most cancers, though LC demonstrated earlier acceleration around age 40.

Male predominance in absolute DALYs persisted for EC, GC, LC, PC, and CRC—often pronounced in high-burden nations such as China. GBTC again deviated, demonstrating clear female predominance particularly evident in China and India. These geographic, age, and sex patterns held for absolute incidence, prevalence, and mortality as well (Figures S28–S33).

### Long-term trends in ASRs (joinpoint analysis)

Joinpoint analysis of ASDR trends from 1990 to 2023 revealed divergent regional trajectories (Figures S34–S39). East Asia and high-income Asia Pacific achieved long-term declines for EC, GC, LC, and GBTC—exemplified by GC average annual percent change (AAPC) of −3.49% and −3.77%, respectively. South Asia confronted contrasting deterioration: EC (AAPC = +1.74%), PC (AAPC = +1.53%), GBTC (AAPC = +1.07%), and CRC (AAPC = +0.69%) all increased. Southeast Asia experienced similar escalation for PC (AAPC = +1.54%) and CRC (AAPC = +1.30%).

The final trend segment, which began in 2019, 2020, or 2021 depending on the location and cancer type, exhibited a dominant and concerning pattern: previously favorable declines in East Asia and high-income Asia Pacific abruptly reversed into significantly increasing ASDR trends. East Asian GC declined rapidly through 2020 (annual percent change [APC], 2017–2020: −6.94%) before reversing sharply (APC, 2020–2023: +2.04%). Similar significant reversals affected other malignancies across these regions.

PC exhibited particularly alarming dynamics. South Asia’s persistent long-term increases (AAPC = +1.53%) accelerated dramatically in recent years (APC, 2015–2023: +4.46%), while East Asia and high-income Asia Pacific experienced pandemic-era reversals, with recent APCs of +4.19% and +3.24%, respectively. CRC also demonstrated unfavorable trends: ongoing Southeast Asian increases (recent APC, 2018–2023: +1.72%) compounded sharp reversals in East Asia (APC, 2020–2023: +3.82%) and high-income Asia Pacific (APC, 2021–2023: +2.57%). ASIR, and ASPR strongly corroborated these ASDR findings, particularly the critical pandemic-era reversals and persistently rising PC and CRC burden across multiple regions (Figures S34–S39). Similar patterns emerged in mortality trends (ASMR APC 2020–2023: +1.99%), consistent with the DALY trajectory.

Country-level AAPCs for all 34 Asian nations and territories are provided in Table S1.

### APC in ASRs by country and region during the COVID-19 pandemic era (final trend segment, 2019–2023)

**Figure 2:**
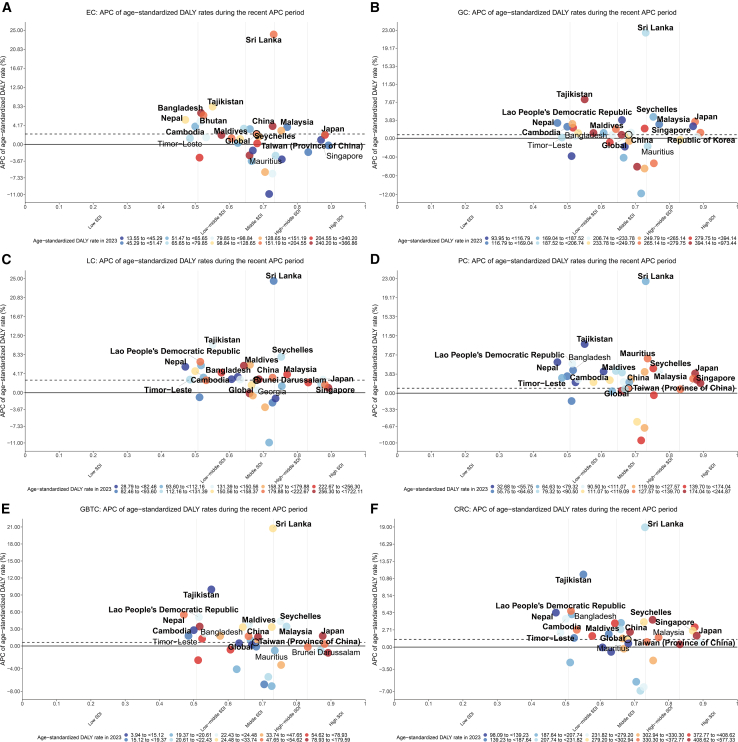
Final segment APC in age-standardized disability-adjusted life year (DALY) rates for six major digestive system cancers across Asian countries and territories, highlighting trends during the COVID-19 pandemic and leading countries by SDI quintile (A) Esophageal cancer (EC). (B) Gastric cancer (GC). (C) Liver cancer (LC). (D) Pancreatic cancer (PC). (E) Gallbladder and biliary tract cancer (GBTC). (F) Colorectal cancer (CRC). Scatterplots display the annual percent change (APC) from the final trend segment (ending 2023) identified by joinpoint regression (*y* axis) against the SDI quintile (*x* axis) for Asian countries and territories. The color of each point indicates the region’s age-standardized DALY rate in 2023 (per 100,000 population). Color scales rely on quantile classification (specifically deciles) calculated independently for each cancer type to maximize visual contrast across the diverse burden ranges; thus, identical colors in different graphs represent the same relative percentile rank rather than the same absolute rate. The horizontal dashed line represents the Global APC specifically for the 2019–2023 period; points falling above this line indicate a burden acceleration exceeding the global average, while points below indicate a more favorable trajectory. Vertical gray lines delineate the 5 SDI quintiles (low to high) based on 2023 SDI values for the 34 Asian locations, grouping countries by their developmental status. Labels identify the three countries or territories with the highest APC within each SDI quintile. Labels are bolded if the final trend segment began in 2019, 2020, or 2021, indicating a trend change during the COVID-19 pandemic period. Abbreviations: SDI, sociodemographic index.

APC analysis of the final trend segments ending in 2023 (initiated between 2019 and 2021) revealed notable burden escalations in several nations, particularly those with developing cancer registry systems (Figure 2). Sri Lanka emerged as the epicenter of crisis, registering catastrophic ASDR increases across every cancer type: LC APC reached +24.49%, EC +24.08%, GC +22.47%, PC +22.47%, GBTC +20.75%, and CRC +18.94%—the highest values for all six malignancies. However, APCs of unusually high magnitude, particularly those showing near-uniform elevation across multiple etiologically distinct cancer types, warrant cautious interpretation. In settings with developing cancer registry systems, such meteoric rises may partially reflect improvements in case ascertainment, changes in reporting practices, or instability in modeled estimates, rather than solely epidemiological shifts. Tajikistan consistently ranked second across all cancer types, with ASDR APCs ranging from +8.26% (EC) to +11.49% (CRC).

Additional nations experienced substantial burden increases during this period. The Lao People’s Democratic Republic recorded a CRC APC of +5.71%, Bangladesh an EC APC of +6.88%, and Nepal a GBTC APC of +5.51%. Seychelles recorded notable increases for both GC (+4.55%) and LC (+7.87%), while Mauritius demonstrated PC acceleration, with an APC of +6.95%. Parallel findings emerged across incidence, mortality, and prevalence metrics (Figures S40–S42). Detailed statistical metrics for all identified trend segments, including specific APC values, 95% confidence intervals, and *p* values, are presented in Table S2.

### Ranking of relative burden in 2023 and final segment APC values reflecting the COVID-19 pandemic era

**Figure 3:**
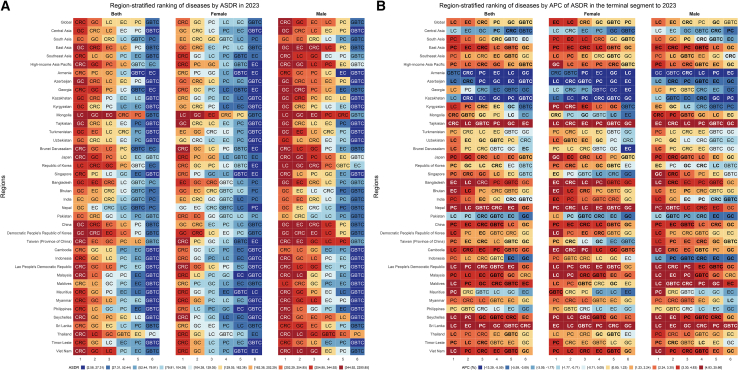
Comparative ranking of six major digestive system cancers based on 2023 DALY rates and recent trends (final segment APC), across geographic regions by sex Cell color corresponds to the magnitude of the ranked metric. (A) DALY rates. (B) Final segment APC values. The color scale utilizes quantile classification (deciles) derived from the pooled distribution of values across all 6 cancer types, enabling direct visual comparison of burden magnitude between different malignancies. In (B), cancer abbreviations within cells are bolded if the final trend segment began in 2019, 2020, or 2021, indicating a trend change during the COVID-19 pandemic period. Abbreviations: DALY, disability-adjusted life years; ASDR, age-standardized disability-adjusted life year rate; APC, annual percent change; EC, esophageal cancer; GC, gastric cancer; LC, liver cancer; PC, pancreatic cancer; GBTC, gallbladder and biliary tract cancer; CRC, colorectal cancer.

Regional heterogeneity was evident in 2023 burden rankings by ASDR (Figure 3A). GC and CRC imposed the greatest ASDR burden across East Asia, Central Asia, and high-income Asia Pacific. South Asia presented a contrasting profile: EC constituted the principal burden. LC ranked as the second-leading cause in Southeast Asia, while PC occupied third position in high-income Asia Pacific. Sex-stratified analysis revealed largely parallel burden rankings for males and females.

Recent ASDR trends—measured by APC in the terminal period through 2023 encompassing the pandemic—exposed profoundly negative trajectories (Figure 3B). PC demonstrated the most rapid acceleration, claiming first rank for highest ASDR APC across four of five major Asian regions: East Asia, Southeast Asia, high-income Asia Pacific, and South Asia. LC exhibited widespread deterioration, ranking second for ASDR APC in South Asia, Southeast Asia, and high-income Asia Pacific. CRC and EC also ranked among the three fastest-growing burdens in East Asia and high-income Asia Pacific, though generally at lower magnitudes than PC.

These DALY-based rankings were consistent with rankings based on incidence, mortality, and prevalence (Figures S43–S45).

### Decomposition analysis of changes in absolute burden (1990–2023)

**Figure 4:**
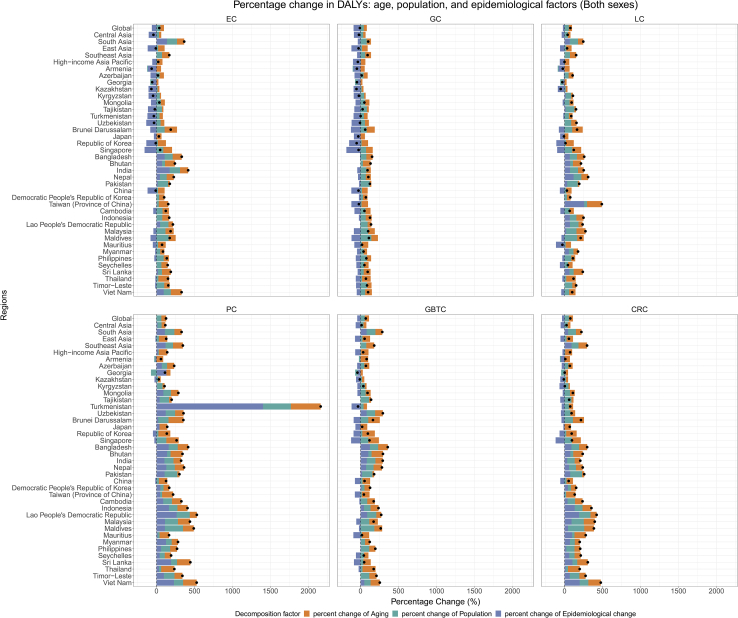
Decomposition analysis of the percentage change in absolute DALY numbers between 1990 and 2023 for six major digestive system cancers across Asian regions The total percentage change (indicated by points) is attributed to changes in population aging, population growth, and epidemiological factors (changes in age-specific DALY rates), represented by the colored bars. Abbreviations: DALY, disability-adjusted life year; EC, esophageal cancer; GC, gastric cancer; LC, liver cancer; PC, pancreatic cancer; GBTC, gallbladder and biliary tract cancer; CRC, colorectal cancer.

Decomposition analysis addressed two questions: which cancers and countries showed epidemiological deterioration that compounded demographic pressures, and which showed epidemiological improvements that offset them. Population aging and growth dominated the expansion of absolute DALY burden for most digestive cancers between 1990 and 2023 across Asian nations (Figure 4). Epidemiological change, however, exerted profoundly variable influence. Adverse epidemiological trends—manifesting as increasing age-specific rates—intensified PC DALY burden dramatically in Vietnam (+228.92%), Sri Lanka (+192.50%), and Georgia (+130.75%). An extreme value was also observed in Turkmenistan (+1403.41%); however, this likely reflects a low 1990 baseline and modeling instability in data-sparse settings, and should be interpreted with caution. CRC experienced comparable epidemiological deterioration in Vietnam (+198.61%), Sri Lanka (+103.78%), and Mauritius (+99.03%). LC burden escalated through unfavorable epidemiological shifts in Nepal (+118.58%) and Sri Lanka (+63.79%), while EC intensified in Vietnam (+95.28%), Nepal (+44.29%), and Sri Lanka (+20.88%). These epidemiological forces, compounding demographic pressures, drove particularly severe overall DALY increases: Vietnam's total DALYs grew by 529.00% for PC and 476.88% for CRC; Sri Lanka's grew by 446.23% for PC and 306.11% for CRC. Azerbaijan also showed notable DALY growth for both PC (+232.84%) and LC (+106.44%), reflecting combined demographic and epidemiological pressures of moderate magnitude.

Contrasting trajectories emerged where epidemiological improvements substantially counteracted demographic pressures. Note that strongly negative epidemiological contributions (below −100%) indicate that substantial improvements in age-specific rates were powerful enough to completely neutralize the upward drivers of population aging and growth, resulting in a net decrease in absolute burden. GC benefited from favorable epidemiological shifts across most analyzed nations, yielding overall DALY reductions in Japan (−87.16% contribution), Republic of Korea (−150.40%), and China (−120.88%). EC experienced similarly robust epidemiological improvements, fully neutralizing demographic pressures in Republic of Korea (−134.15%), Armenia (−113.33%), China (−117.54%), and Kazakhstan (−105.14%), with substantial though incomplete offsets in Azerbaijan (−78.58%) and Georgia (−50.96%). LC burden declined or stabilized through favorable epidemiological change in multiple nations, most strongly in Republic of Korea (−108.05%) and Mauritius (−112.96%), with progressively smaller offsets in Kazakhstan (−88.87%), Armenia (−77.84%), China (−61.78%), Japan (−57.77%), and Georgia (−18.52%). For CRC, PC, and GBTC, epidemiological improvements materialized in specific locations but generally proved insufficient to offset demographic momentum; EC showed more widespread epidemiological offsets in select nations (as detailed above). Parallel decomposition analyses for absolute incidence, prevalence, and mortality are provided in Figures S46–S48.

### Socioeconomic inequalities in age-standardized burden and temporal trends (1990 vs. 2023)

**Figure 5:**
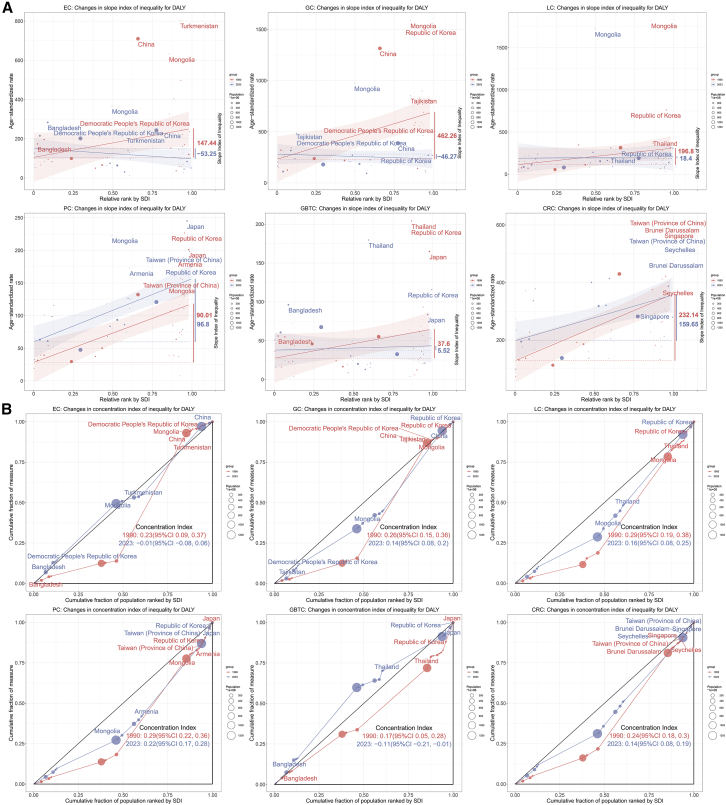
Changes in absolute inequality (slope index of inequality) and relative inequality (concentration index) for age-standardized DALY rates of six major digestive system cancers across the SDI spectrum, 1990–2023 (A) Changes in absolute inequality (slope index of inequality). Scatterplots display age-standardized rates (per 100,000 population) in 1990 (red points/line) and 2023 (blue points/line) against the relative rank of countries/territories by SDI. Labels indicate the 3 countries or territories with the highest age-standardized rates in 1990 and 2023, respectively. (B) Changes in relative inequality (concentration index). Concentration curves illustrating relative inequality in age-standardized rates for six major digestive system cancers across the SDI spectrum in 1990 (red line/points) and 2023 (blue line/points). Labels indicate the three countries or territories with the highest age-standardized rates in 1990 and 2023, respectively. Abbreviations: DALY, disability-adjusted life year; EC, esophageal cancer; GC, gastric cancer; LC, liver cancer; PC, pancreatic cancer; GBTC, gallbladder and biliary tract cancer; CRC, colorectal cancer; SDI, sociodemographic index.

Socioeconomic disparities underwent reconfiguration between 1990 and 2023 (Figure 5A). Positive SII values indicate higher burden in high-sociodemographic index (SDI) populations (pro-rich distribution), while negative values indicate concentration in low-SDI populations (pro-poor distribution). The SII declined substantially for multiple cancers. EC shifted in direction and magnitude—from pronounced high-SDI concentration (SII: 147.44 per 100,000) to modest low-SDI skewing (SII: −53.25 per 100,000), with the 2023 absolute disparity substantially smaller than in 1990. GC shifted from steep inequality favoring high-SDI nations (SII: 462.26) to modest low-SDI concentration (SII: −46.27). LC and GBTC inequality contracted markedly in both absolute and relative terms: LC fell from 196.80 to 18.40 (−91%), while GBTC decreased from 37.60 to 5.52 (−85%). PC maintained relatively stable absolute inequality, changing minimally from 90.01 to 96.80. CRC inequality diminished moderately—from 232.14 to 159.65. Incidence, mortality, and prevalence SII trends largely paralleled these DALY patterns (Figures S49–S51).

The CIX trends largely paralleled SII patterns, with most cancers showing declines in the 1990–2023 period (Figure 5B). All cancers demonstrated pro-rich inequality in 1990: GC exhibited CIX of 0.26, LC reached 0.29, and CRC registered 0.24. By 2023, EC approached distributional neutrality (CIX: −0.01), while GC (0.14), LC (0.16), PC (0.22), and CRC (0.14) showed attenuated yet persistent pro-rich concentration. GBTC underwent striking transformation, transitioning to pro-poor inequality (CIX: −0.11)—signaling disproportionate burden shift toward lower-SDI countries by 2023. Mortality CIX trajectories generally tracked DALY trends. Prevalence inequality, however, diverged notably: relative disparities stabilized or intensified for GC, CRC, and PC, indicating widening gaps in prevalent case concentration among higher-SDI nations (Figures S52–S54).

### Behavioral (including dietary) and metabolic risk factors driving digestive cancer burden (2023)

Modifiable risk factors accounted for substantial proportions of digestive cancer ASDR across Asian regions in 2023 (Figures S55–S72). These risk-attributable fractions, derived from the GBD comparative risk assessment framework, are not strictly additive due to potential overlap and mediation between risks (e.g., dietary patterns influencing BMI, which in turn affects fasting plasma glucose). Dietary patterns emerged as particularly influential drivers. Inadequate whole grain, calcium, milk, and fiber consumption each contributed significantly to CRC ASDR—frequently exceeding 10% in East Asia, Southeast Asia, and high-income Asia Pacific. Excessive red meat and processed meat intake contributed similarly to CRC burden in these higher-SDI regions and Central Asia. High sodium consumption contributed to GC ASDR consistently across most populations (approximately 6%–8%), while insufficient vegetable intake contributed moderately yet ubiquitously to EC ASDR.

Behavioral exposures—principally smoking and alcohol—represented dominant contributors to multiple cancer ASDRs, exhibiting pronounced male predominance. Smoking constituted the primary risk factor for EC, particularly among males in East Asia, Central Asia, and high-income Asia Pacific, where attributable fractions often surpassed 40%. This exposure also drove substantial GC, LC, and PC burden in these populations. Alcohol use ranked second for EC ASDR, again demonstrating strong male concentration in East Asia, high-income Asia Pacific, and Southeast Asia, while contributing significantly to LC and GC burden particularly among males. In South Asia, tobacco chewing represented a regionally specific EC risk factor. Drug use drove extraordinarily large fractions of LC ASDR in Central Asia, exemplified by Kazakhstan and Kyrgyzstan. However, given the well-documented challenges in obtaining precise illicit drug use data, these attributable estimates should be interpreted with caution.

Metabolic derangements exerted substantial influence. Elevated body-mass index (BMI) contributed notably to CRC, LC, GBTC, and PC ASDRs, with maximal attributable fractions concentrated in Central Asia. Elevated fasting plasma glucose emerged as a major PC ASDR driver—frequently exceeding 20%—while contributing moderately to LC and CRC burden. Physical inactivity demonstrated moderate attributable burden for CRC across regions. ASMR attributable fractions largely paralleled these ASDR patterns across all risk factors and malignancy types (Figures S55–S72).

### Projections of future burden to 2040

**Figure 6:**
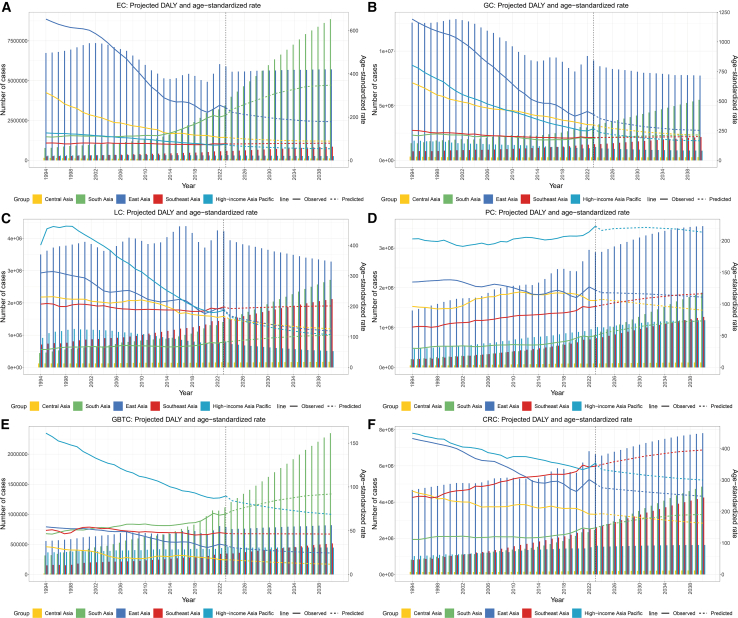
Nordpred projections of absolute DALYs and age-standardized rates for six major digestive system cancers in Asian regions, 2024–2040 (A) Esophageal cancer (EC). (B) Gastric cancer (GC). (C) Liver cancer (LC). (D) Pancreatic cancer (PC). (E) Gallbladder and biliary tract cancer (GBTC). (F) Colorectal cancer (CRC). Abbreviations: DALY, disability-adjusted life year.

Predictive modeling through 2040 reveals a consistent pattern: divergent ASDR trajectories alongside absolute DALY escalation for most digestive cancers, reflecting the dominance of demographic forces over rate improvements (Figure 6). These forecasts use a 1994–2023 fitting window, explicitly incorporating pandemic-era shifts (2019–2023) into baseline assumptions. East Asia and high-income Asia Pacific are projected to sustain ASDR declines observed from 1994 to 2023 for GC, LC, and EC. Contrasting projections emerge for South and Southeast Asia. In South Asia, ASDRs (per 100,000) are projected to rise for EC (from 208.76 to 347.96), CRC (147.94 to 190.74), LC (83.23 to 105.36), PC (49.95 to 73.48), and GBTC (69.10 to 92.01). Southeast Asia is projected to experience increases for CRC (344.35 to 395.57), PC (96.29 to 116.44), and LC (198.56 to 201.62).

Regional ASDR improvements notwithstanding, absolute DALY burden will escalate substantially across nearly all regions for most cancers between 2023 and 2040—driven predominantly by demographic transformation. South Asia faces projected increases, led by an unprecedented 2.6-fold rise in EC DALYs from 3.44 million to 8.86 million, followed by PC (0.81 to 1.88 million), GBTC (1.11 to 2.35 million), CRC (2.46 to 4.85 million), and LC (1.41 to 2.71 million). Southeast Asia faces similarly concerning trajectories—CRC escalating from 2.62 million to 4.25 million, PC from 0.74 million to 1.27 million, LC from 1.53 million to 2.12 million, EC from 0.60 million to 0.86 million, and GC from 1.48 million to 2.13 million. Even East Asia, despite achieving ASDR declines for most malignancies, will experience absolute CRC DALY growth from 6.65 million to 7.81 million. Parallel projections for absolute incidence, prevalence, and mortality through 2040 are provided in Figures S73–S75, all indicating continued expansion of the digestive cancer burden across Asia if current trends persist.

## Discussion

This pan-Asian analysis spanning three decades demonstrates the profound heterogeneity in digestive cancer dynamics across regions, malignancy types, and temporal phases. Population aging and growth drive increases in absolute disease burden throughout much of the continent, even where ASRs decline—a phenomenon particularly evident in East Asia and the high-income Asia Pacific. Significant trend reversals coincided with the COVID-19 era: previously favorable trajectories shifted in multiple settings, with PC and CRC demonstrating the most widespread recent acceleration, though the durability of these reversals remains to be confirmed with additional data. Modifiable dietary, metabolic, and behavioral exposures account for substantial risk-attributable fractions that correspond closely to observed sex-specific patterns and regional disparities, identifying potential targets for prevention and health-system intervention.

The regional patterns observed here reflect distinct epidemiological trajectories across Asia. Among the most adverse long-term patterns, South Asia saw EC incidence rise 360.27% and mortality 405.90% in absolute terms, accompanied by substantial increases in ASRs (ASIR +74.72%; ASMR +91.02%). GBTC followed similar expansionary paths in both raw counts and adjusted rates. Southeast Asia experienced substantial growth in CRC and PC—incidence rose by roughly five-fold for both CRC and PC, accompanied by sizable rate increases—alongside mounting burdens from LC and GBTC. Contrasting sharply with these patterns, East Asia and the high-income Asia Pacific achieved substantial reductions in ASMR and DALY rates for EC, GC, and LC; absolute case numbers nonetheless often climbed, exemplifying how demographic forces can undermines epidemiological gains. Central Asia recorded some of the most encouraging rate declines, particularly for EC and GC. This heterogeneity directly addresses the study’s foundational objective: disentangling demographic forces from true epidemiological shifts at scale while demonstrating that rate improvements may paradoxically coexist with expanding clinical demands.^18^

National profiles add critical granularity to these regional patterns. Mongolia shoulders the highest ASDR for LC, GC, and EC while ranking second for PC (after Japan); Bangladesh records the second-highest EC and third-highest GBTC burdens, while the Democratic People's Republic of Korea confronts similarly extreme EC and GC burdens. High-income and rapidly modernizing economies face distinct challenges characteristic of lifestyle- and metabolism-related cancers: Taiwan (Province of China) records the highest CRC burden despite its high SDI status, Thailand exhibits peak GBTC DALY rates alongside exceptionally high LC burden, and the Republic of Korea ranks near the summit for LC and GBTC. Such distributions underscore that socioeconomic advancement reshapes rather than eliminates cancer burden, with high-SDI nations facing a distinct spectrum dominated by lifestyle- and metabolism-related malignancies.^19^^,^^20^

Demographic patterns across age and sex are consistent with established risk factor distributions. DALY rates escalate sharply with advancing age for all six malignancies, though LC exhibits earlier acceleration than other digestive cancers, reflecting the typical 20–30 year interval between chronic HBV/HCV infection and hepatocellular carcinogenesis. Male predominance characterizes EC, GC, LC, PC, and CRC, while GBTC reverses this pattern, demonstrating female preponderance most pronounced in South Asia. These sex-specific and age-related distributions correspond closely to documented risk-attributable fractions: smoking and alcohol drive substantial proportions of upper gastrointestinal malignancies, sodium-rich diets contribute consistently to GC burden, elevated fasting plasma glucose and BMI fuel PC and CRC, tobacco chewing in South Asia drives EC burden, and drug use reportedly contributes to substantial LC burden in Central Asian subregions, though these estimates carry uncertainty due to data limitations. This concordance between epidemiological patterns and risk factor distributions provides a rationale for targeted prevention strategies.^21^^,^^22^^,^^23^^,^^24^

Joinpoint analysis identified statistically significant trend reversals beginning in 2019–2021, interrupting previously favorable trajectories in multiple settings. East Asian GC DALY rates exemplify this pattern: APC shifted from −6.94% during 2017–2020 to +2.04% during 2020–2023, with parallel reversals affecting other malignancies. The high-income Asia Pacific exhibited similar temporal disruptions. PC emerges as particularly concerning, with recent APC of +4.19% in East Asia and +3.24% in the high-income Asia Pacific. CRC similarly accelerated, reaching +3.82% annually in East Asia and +2.57% in the high-income Asia Pacific during recent intervals. National granularity reveals especially acute surges: Sri Lanka and Tajikistan recorded the highest APCs across all six cancers, though these values in settings with developing registries may partially reflect data-quality issues rather than solely epidemiological shifts; the Lao People’s Democratic Republic, Bangladesh, Nepal, Seychelles, and Mauritius documented additional notable increases. While this descriptive analysis cannot establish causation, several mechanisms may contribute to these temporal patterns. Possible contributors to the observed reversals include widespread interruptions to cancer screening programs, delays in symptomatic diagnosis, and discontinuities in treatment regimens reported globally during the crisis.^14^ Non-pandemic factors, such as health system reforms, economic shocks, or shifts in risk factor prevalence, may also contribute to these trend shifts.^25^^,^^26^ Future research linking Asian cancer registry data with healthcare utilization and screening coverage records during 2019–2023 is needed to validate these hypotheses and inform post-pandemic recovery strategies.

Decomposition analysis reveals the forces propelling escalating case counts. Population aging and growth dominate the expansion of absolute DALYs across most settings, yet genuine epidemiological deterioration—worsening age-specific rates—substantially amplifies particular malignancies in specific nations. PC exemplifies this pattern in Vietnam (+228.92%), Sri Lanka (+192.50%), and Georgia (+130.75%); an extremely high value in Turkmenistan (+1403.41%) likely reflects data instability CRC demonstrates comparable epidemiological worsening in Vietnam, Sri Lanka, and Mauritius; LC and EC similarly deteriorated in select countries including Nepal and Sri Lanka. Contrasting sharply with these trends, East Asia and the high-income Asia Pacific achieved major favorable epidemiological shifts for GC, while EC improvements extended more broadly to include Central Asia and the Caucasus region. This divergence reflects the maturation of control programs for GC (e.g., *Helicobacter pylori* [*H. pylori*] testing) and LC (e.g., hepatitis B vaccination), as well as reductions in EC risk factors including smoking, in contrast to the expansion of diet- and metabolism-linked malignancies.^4^^,^^27^^,^^28^^,^^29^ These decomposition results inform strategic priorities: East Asia, where demographic forces dominate, requires expanded clinical care capacity while maintaining prevention efforts; South and Southeast Asia, facing both demographic pressure and worsening age-specific rates, require urgent primary prevention alongside capacity building.^30^^,^^31^

Inequality analyses document reduced absolute inequality for most cancer types alongside persisting gradients. Absolute DALY inequality declined substantially for GC, EC, and LC, and modestly for GBTC, with relative inequality declining similarly for most anatomical sites. Burdens nonetheless remain concentrated in higher-SDI countries for CRC, PC, GC, and LC, revealing incomplete redistribution. GBTC notably shifted to pro-poor concentration by 2023, whereas PC was distinctive in showing no meaningful decline in absolute inequality. Prevalence inequality either stabilized or widened for GC, CRC, and PC, which may partly reflect the accumulation of long-term survivors in resource-rich health systems, though this hypothesis requires direct survival analyses to confirm. Addressing these distributional patterns will require context-appropriate strategies targeting screening access, early detection, and survivorship care, while accounting for resource constraints in lower-SDI settings.^31^^,^^32^^,^^33^^,^^34^

Projections extending to 2040 highlight the magnitude of future burden if current trends persist, underscoring the need for large-scale preventive and clinical interventions. Demographic forces are projected to outweigh rate improvements in numerous settings, driving absolute DALY increases even where ASRs decline—a phenomenon anticipated for GC, LC, and EC in East Asia and the high-income Asia Pacific (Figure 6). South and Southeast Asia face particularly concerning prospects: ASRs for PC and CRC are projected to continue climbing while absolute DALYs are projected to increase substantially. South Asian EC DALYs may escalate from 3.44 to 8.86 million, CRC from 2.46 to 4.85 million, LC from 1.41 to 2.71 million, GBTC from 1.11 to 2.35 million, and PC from 0.81 to 1.88 million. Southeast Asia faces substantial but generally smaller-magnitude expansion, while East Asian absolute CRC DALYs are forecast to climb from 6.65 to 7.81 million. These trajectories necessitate “double-duty” strategic frameworks. For infection-related cancers, momentum must be sustained through vaccination and treatment access. To curb the rising tide of lifestyle-related malignancies, policy priorities must shift toward scaling up CRC screening in transitioning economies, implementing *H. pylori*-based GC prevention programs and complementary dietary interventions including sodium reduction, and strengthening efforts to address metabolic risk factors, including obesity and diabetes, despite the well-documented challenges of population-level behavioral change.^35^^,^^36^^,^^37^ Furthermore, establishing resilient diagnostic pathways is essential to ensure that future systemic shocks do not again derail cancer control progress.

From a public health perspective, the divergent trajectories we observed—sustained declines in infection-related cancers in high-SDI settings juxtaposed with the rapid acceleration of CRC and PC coinciding with the pandemic era in many parts of Asia—support a “portfolio” prevention strategy that matches interventions to local risk profiles. For liver cancer, sustaining high coverage of universal hepatitis B vaccination (including timely birth dose) and strengthening perinatal prevention and linkage-to-care for chronic HBV infection remain high-yield approaches, with Taiwan’s long-term experience demonstrating marked reductions in hepatocellular carcinoma among vaccinees.^27^^,^^28^ For GC, population-based *H. pylori* “test-and-treat” programs in high-risk communities have shown promise in reducing incidence, exemplified by the Matsu Islands long-term cohort,^38^ though scaling to larger populations presents feasibility challenges detailed below. For CRC, scaling up organized screening with fecal immunochemical testing (FIT) followed by quality-assured colonoscopy for positive tests—an approach with sustained reductions in advanced-stage disease and CRC mortality in Taiwan—could directly counter the rising CRC burden highlighted by our joinpoint and projection results.^39^ Our attributable-risk findings identify tobacco and alcohol use, dietary factors (high sodium, low whole grains/fiber), and cardiometabolic risks (high BMI and hyperglycaemia) as key drivers across multiple digestive cancers. Upstream fiscal and regulatory measures, together with integrated primary-care NCD services, should prioritize: (i) smoking cessation and alcohol harm reduction (targeting EC, LC, GC, and PC); (ii) sodium reduction (primarily for GC); and (iii) obesity and diabetes prevention (primarily for CRC and PC). Nevertheless, implementation remains challenging across much of Asia: endoscopy/colonoscopy capacity and quality assurance are uneven, participation and follow-up can amplify SDI-linked inequities, antimicrobial resistance complicates *H. pylori* eradication strategies, and recent trend reversals underscore the need for resilient diagnostic pathways and feasible catch-up strategies to minimize avoidable mortality.^14^ For PC, population screening is not recommended; while risk-stratified surveillance in expert centers may enable earlier-stage detection and improved survival among selected high-risk individuals, scaling such programs equitably remains a major barrier.^40^

In summary, the pan-Asian assessment delineates a dual reality: durable progress against infection-related cancers (LC, GC) and smoking-related EC juxtaposed with a rapid, pandemic-era reversal and acceleration of colorectal and pancreatic cancer trends, all unfolding against a backdrop of demographic change. The data identify countries and cancers where epidemiological deterioration has emerged as a significant driver of growth, alongside demographic pressures, spotlight key risk factors—notably tobacco, alcohol, dietary patterns, and cardiometabolic factors—whose control is likely to yield the largest returns, and forecast mounting service needs even where rates decline. Translating these insights into a cohesive dual strategy—sustaining infection control while aggressively targeting metabolic and dietary risks—with resilient diagnostic pathways as an enabling foundation.

### Limitations of the study

Several limitations should be considered when interpreting these findings. First, estimates depend on modeled inputs subject to varying data completeness. In lower-resource settings such as South and Southeast Asia, reliance on covariate-based predictions may introduce uncertainty compared to nations with comprehensive surveillance^41^; similarly, extreme trend estimates in data-sparse nations (e.g., Sri Lanka, Tajikistan) warrant caution regarding potential joinpoint model instability; similarly, extreme decomposition contributions (e.g., Turkmenistan) should be interpreted with awareness of baseline data quality. Second, methodological definitions impose specific constraints: our use of 2023 SDI values creates a fixed cohort that ignores historical socioeconomic shifts, while our definition of “pandemic-era trends” represents a design choice where alternative segmentations might yield different inflection points. Third, the study’s macro-level focus precluded etiologic stratification for liver cancer (e.g., viral vs. metabolic) and analysis of synergistic risk factor interactions or sub-national disparities (e.g., urban-rural), which are not uniformly available in the aggregated dataset. Finally, burden projections assume the continuation of historical patterns. While including the 2019–2023 period captures recent shifts, this approach carries the risk of extrapolating temporary pandemic-era distortions into long-term forecasts without accounting for future policy interventions or therapeutic breakthroughs.

## Resource availability

### Lead contact

Further information and requests for resources should be directed to and will be fulfilled by the lead contact, Jingdong Zhang (jdzhang@cancerhosp-ln-cmu.com).

### Materials availability

This study did not generate new unique reagents.

### Data and code availability

• Data are available in a public, open access repository. Data from the GBD study in 2023 can be accessed using the Global Health Data Exchange (GHDx) query tool (https://vizhub.healthdata.org/gbd-results/) which the Institute for Health Metrics and Evaluation maintains.
• This article does not report original code.
• Any additional information required to reanalyze the data reported in this paper is available from the lead contact upon request.

## Acknowledgments

This work was supported by the Empowerment Program - Scientific Research Fund Project (grant no. KC2023-JX-0288-FQ08) from 10.13039/501100012401Beijing Science and Technology Innovation Medical Development Foundation; the 10.13039/501100012506Scientific research foundation for the introduction of talents, 10.13039/501100020761Liaoning Cancer Hospital & Institute (grant no. Z1702); 10.13039/100012542Liaoning Provincial Science and Technology Joint Plan Project (grant no. 2024JH2/102600192); Liaoning Talent Development Plan-Medical Experts (grant no. YXMJ-LJ-17); and the 10.13039/501100012226Fundamental Research Funds for the Central Universities Cross-disciplinary Medical-Engineering Collaboration Project (10.13039/501100020761Liaoning Cancer Hospital & Institute-10.13039/501100002980Dalian University of Technology, grant no. LD202025).

## Author contributions

Conceptualization, J.Z. and L.Z.; methodology, L.Z. and F.L.; investigation, L.Z. and F.L.; writing – original draft, L.Z.; writing – review and editing, J.Z.; funding acquisition, F.L. and J.Z.; resources, F.L. and J.Z.; supervision, J.Z.

## Declaration of interests

The authors declare no conflicts of interest.

## STAR★Methods

### Key resources table

**Table:** 

REAGENT or RESOURCE	SOURCE	IDENTIFIER
**Deposited data**		

Global Burden of Disease Study 2023	Data from the GBD study in 2023 can be accessed using the Global Health Data Exchange (GHDx) query tool	https://vizhub.healthdata.org/gbd-results/

**Software and algorithms**		

R version 4.3.1	R Core Team	https://www.r-project.org/
Joinpoint Regression Program (Version 5.4.0)	National Cancer Institute	https://surveillance.cancer.gov/joinpoint/
Nordpred (R package, Version 1.1)	Cancer Registry of Norway	https://www.fhi.no/en/cancer/data-and-statistics/nordpred/
ggplot2 (R package, Version 3.4.2)	CRAN	https://ggplot2.tidyverse.org/

### Experimental model and study participant details

This study is a secondary analysis utilizing openly accessible, geographically aggregated data from the Global Burden of Disease (GBD) 2023 database. As such, it did not involve the direct enrollment of human participants, nor did it use experimental models of any kind. All analyzed data were fully anonymized and aggregated at the national or regional level. Because this study used only publicly available, aggregated, and fully anonymized data with no individual-level records, individual patient consent was not required. Patients and the public were not involved in the design, conduct, or reporting of this study. Furthermore, this study follows the Guidelines for Accurate and Transparent Health Estimates Reporting (GATHER) framework where applicable to secondary analyses of global health estimates. This study did not require institutional ethical approval under local regulations because it used only publicly available, anonymized, aggregated data with no individual-level records.

In accordance with the journal's guidelines on sex and gender reporting, all analyses in this study were stratified by biological sex, and sex-specific findings are reported throughout the Results (see also Distribution of absolute burden by age, sex, and region, and Risk factor sections).

#### Study design

We analyzed burden estimates for six major digestive malignancies: esophageal cancer (EC), gastric cancer (GC), liver cancer (LC), pancreatic cancer (PC), gallbladder and biliary tract cancer (GBTC), and colorectal cancer (CRC).^17^ Our analysis covered the period 1990–2023, capturing three decades of epidemiological evolution across all 34 countries and territories constituting the five GBD-defined Asian regions (East, Southeast, South, Central, and High-income Asia Pacific). This study adheres strictly to the GBD regional classification, which differs from other geopolitical definitions (e.g., World Health Organization), and includes the complete set of locations available in the dataset for these regions.^42^ Global estimates provided comparative context. While we assessed incidence, prevalence, mortality, and disability-adjusted life year (DALY) comprehensively, this report primarily emphasizes DALYs to capture the holistic burden of healthy life loss, with secondary attention to other metrics.

Stratification employed multiple dimensions: five GBD-defined Asian regions, individual national entities, standard 5-year age bands extending from 0 to 4 years through ≥95 years, and sex.^42^ Socioeconomic gradients were examined using the sociodemographic index (SDI), a composite metric ranging from 0 to 1 that integrates national income per capita, educational attainment, and total fertility rates as calculated by the GBD study.^43^ We grouped the 34 Asian nations and territories into five SDI-based quintiles (low, low-middle, middle, high-middle, high) using 2023 values. This static classification, which may not fully reflect historical socioeconomic transitions for countries that shifted between quintiles, is discussed in Limitations. Risk factor attribution and burden projections extending to 2040 completed our analytical framework.^44^^,^^45^ All estimates—absolute counts and age-standardized rates—derive directly from GBD database outputs without modification.

Malignancy definitions strictly followed the GBD 2023 protocols, encompassing malignant neoplasms and, where specified by GBD methodology for DALY estimation, neoplasms of *in situ* or uncertain behavior to ensure database consistency. Detailed International Classification of Diseases, Tenth Revision (ICD-10) codes mapping to each of the six cancer causes are provided in Table S3.

### Method details

#### Outcome measures

We analyzed four outcome measures: incidence (new cases occurring each year), prevalence (cases alive at a given time point), mortality (deaths occurring each year), and DALYs, with DALYs serving as the primary metric and the other three providing corroborating evidence. DALYs represent the sum of years of life lost to premature mortality (YLLs) and years lived with disability (YLDs), providing a combined measure of fatal and non-fatal burden. Because YLDs contribute minimally to digestive cancer burden (due to the high case-fatality and relatively short survival with advanced disease), DALYs closely track YLL patterns for these malignancies. We therefore report DALYs as the primary metric without separately reporting YLL and YLD. For each outcome, we extracted absolute counts, age-specific rates, and age-standardized rates per 100,000 population from the GBD database—yielding ASIR, age-standardized prevalence rate (ASPR), age-standardized mortality rate (ASMR), and age-standardized DALY rate (ASDR). Standardization employed the GBD world standard population weights, enabling valid comparisons over time and across locations independent of age structure. Point estimates were used throughout; corresponding 95% uncertainty intervals are available in the source GBD database and are discussed where relevant.

### Quantification and statistical analysis

#### Statistical analysis methods

##### Descriptive assessment of disease burden

We extracted absolute counts and age-standardized rates for all four outcome measures at two time points: 1990 (baseline) and 2023 (endpoint). Total percentage change between 1990 and 2023 was obtained directly from GBD database outputs. We examined age-specific rates and counts to characterize burden distribution across demographic strata. National-level geographic variation in 2023 age-standardized rates was visualized using choropleth maps of Asian countries and territories. For each region, we ranked the six cancer types by their relative contribution to total digestive cancer burden (measured as ASDR).

##### Temporal trend analysis via joinpoint regression

Temporal trend characterization was performed using joinpoint regression for the annual age-standardized rate series (ASDR, ASMR, ASIR, and ASPR) from 1990 to 2023.^6^^,^^46^ Following the standard Joinpoint approach, we fitted a segmented log-linear model to the natural logarithm of each age-standardized rate series:

$$\ln\left(R_{t}\right)=\beta_{0}+\beta_{1}t+\sum_{k=1}^{K}\delta_{k}{\left(t-\tau_{k}\right)}^{+}+\varepsilon_{t}$$

*R*_*t*_ denotes the age-standardized rate in calendar year *t*, *K* is the number of joinpoints, *τ*_*k*_ represents the location of the *k*-th joinpoint, *δ*_*k*_ is the change in slope at the *k*-th joinpoint, (*x*)^+^ = *max*(*x*,0), and *ε*_*t*_ is the error term. Up to 5 joinpoints were allowed for each series. The final model was selected using the permutation test method (Monte Carlo permutation testing with 4,500 permutations; significance level α = 0.05). For segment *j*(*j* = 1, …,*K*+1), the annual percent change (APC) was computed by the Joinpoint software as:

$${\text{APC}}_{j}=100\times \left[\exp\left(b_{j}\right)-1\right]$$

*b*_*j*_ is the slope of segment *j* on the log scale. The average annual percent change (AAPC) over the full study period was calculated as a segment-length-weighted average of the segment-specific slope coefficients on the log scale and then back-transformed to the percent-change scale. We defined the “recent APC” as the APC for the final trend segment ending in 2023; this is a working definition specific to this study. When this final segment began in 2019, 2020, or 2021—a window chosen to capture potential pandemic-related disruptions given uncertainty about the precise timing of COVID-19's impact on cancer burden estimates—it was interpreted as reflecting the pandemic-era trend.

##### Socioeconomic inequality analysis

Socioeconomic gradients were quantified using two inequality metrics comparing 1990 and 2023 distributions across the SDI spectrum: the slope index of inequality (SII) for absolute inequality, and the concentration index (CIX) for relative inequality.^43^ The SII was calculated via weighted linear regression of age-standardized rates on the midpoints of the cumulative population distribution ranked by SDI; positive values indicate higher burden in high-SDI populations (pro-rich), while negative values indicate the reverse (pro-poor). SII values carry the same units as the underlying rate (per 100,000 population) and represent the estimated rate difference between hypothetical populations at the extremes of the SDI distribution. The CIX was derived from the area under the concentration curve, quantifying relative inequality ranging from −1 (pro-poor) to +1 (pro-rich).

##### Decomposition analysis of burden changes

Decomposition analysis was used to quantify the change in absolute DALY counts (and secondary counts for incidence, prevalence, and mortality) between 1990 and 2023 using the three-factor decomposition method proposed by Cheng et al.^47^ Total DALY counts in a given year were expressed as the product of total population size, age structure, and age-specific DALY rates. The difference between 1990 and 2023 was partitioned into contributions from population growth, population aging (change in age structure), and epidemiological change (change in age-specific DALY rates). Interaction terms among these three components were allocated symmetrically according to the Cheng formulation.^47^ Relative contributions were expressed as the attributed change divided by the DALY count in 1990 × 100%. Negative contributions indicate that a component (most commonly epidemiological change through declining age-specific rates, but potentially also population decline or shifts in age structure) acted to reduce rather than increase the total DALY count. Relative contributions with an absolute magnitude greater than 100% are mathematically valid when a single component's effect exceeds the 1990 baseline DALY count—for example, when strong reductions in age-specific rates exceeded the effect of demographic growth, resulting in a net burden decrease.

##### Risk factor attribution

We used the GBD comparative risk assessment (CRA) framework to estimate the proportion of ASDR and ASMR attributable to modifiable exposures. In the GBD hierarchy, risk factors are organized into broad behavioral, environmental and occupational, and metabolic domains, with dietary risks constituting a Level 2 subgroup within behavioral risks.^45^ In the present study, all attributable-risk estimates were extracted directly from the GBD 2023 database at the original individual risk-factor level. For descriptive presentation purposes, we grouped the digestive cancer-related risks into three presentation groups: non-dietary behavioral risks (smoking, alcohol use, drug use, tobacco chewing, and low physical activity), metabolic risks (high fasting plasma glucose and high body-mass index [BMI]), and dietary risks (diet low in milk, calcium, fiber, whole grains, and vegetables; diet high in sodium, processed meat, and red meat). We displayed dietary risks separately because they represent a coherent nutritional subset within the GBD framework and because this distinction improves readability when comparing prevention targets across cancers and regions. These presentation groups were not used in model estimation, do not imply mutually exclusive biological pathways, and did not alter any GBD-derived attributable fractions; mediation and overlap between risks were handled within the parent GBD CRA framework.^45^

##### Projections of future burden

We projected future burden from 2024 through 2040 using the nordpred R package, which implements an age-period-cohort model with a power-5 link function.^44^ By utilizing a fitting period of 1994–2023, we explicitly incorporated recent pandemic-era shifts into the baseline assumptions. Projections generate forecasts for both absolute counts and age-standardized rates assuming the continuation of the latent trends observed over this calibration period, including the recent trend shifts observed during 2019–2023, whose durability as long-term projections carries inherent uncertainty. However, the model does not account for future unforeseen disruptions such as new pandemics or major therapeutic breakthroughs.

##### Statistical software

Statistical analyses were primarily conducted using R software (version 4.3.1, R Foundation for Statistical Computing, Vienna, Austria). Joinpoint regression analyses were performed using the Joinpoint Regression Program,^46^ Version 5.4.0 (Statistical Research and Applications Branch, National Cancer Institute, Bethesda, MD, USA). Future burden projections to 2040 were generated using the nordpred package (version 1.1) in R, which applies an age-period-cohort model with a power-link function to limit exponential growth assumptions.^48^ Socioeconomic inequality metrics were computed using standard algorithms adapted for weighted data, and visualizations were produced using the ggplot2 package.

## Supplemental information

Document S1. Figures S1–S75 and Table S3

Table S1. AAPC in age-standardized rates for six major digestive system cancers across 34 Asian countries and territories, 1990–2023, related to Figure 2

Table S2. APC in age-standardized rates for six major digestive system cancers across 34 Asian countries and territories, 1990–2023, related to Figure 2
