# Supplementary material for: Regional and national burden of six major digestive cancers in Asia, 1990–2023: An analysis of the GBD Study 2023
Source: iScience. 2026 Apr 15;29(5):115728. doi: 10.1016/j.isci.2026.115728 (PMC13187620; doi:10.1016/j.isci.2026.115728)
Supplement: Document S1. Figures S1–S75 and Table S3 [file mmc1.pdf]

iScience, Volume *29*

Supplemental information

Regional and national burden of six major digestive cancers in Asia, 1990–2023: An analysis of the GBD Study 2023

Liqun Zhang, Fang Li, and Jingdong Zhang

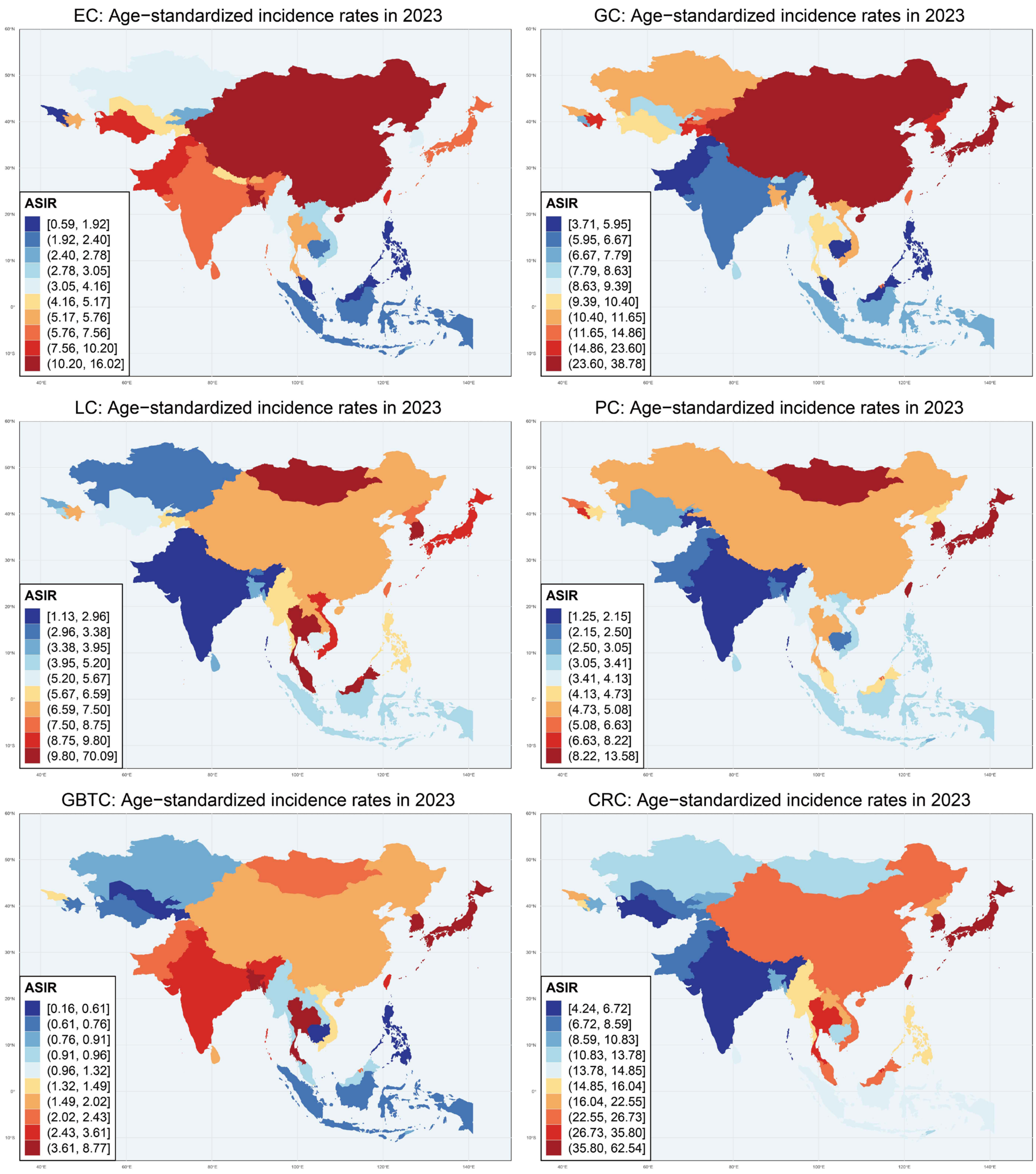

Figure S1: Geographical variation in ASIR for 6 major digestive system cancers by country and territory, 2023. Rates are shown per 100,000 population. (**A**) Esophageal cancer (EC). (**B**) Gastric cancer (GC). (**C**) Liver cancer (LC). (**D**) Pancreatic cancer (PC). (**E**) Gallbladder and biliary tract cancer (GBTC). (**F**) Colorectal cancer (CRC). ASIR, age-standardized incidence rates.

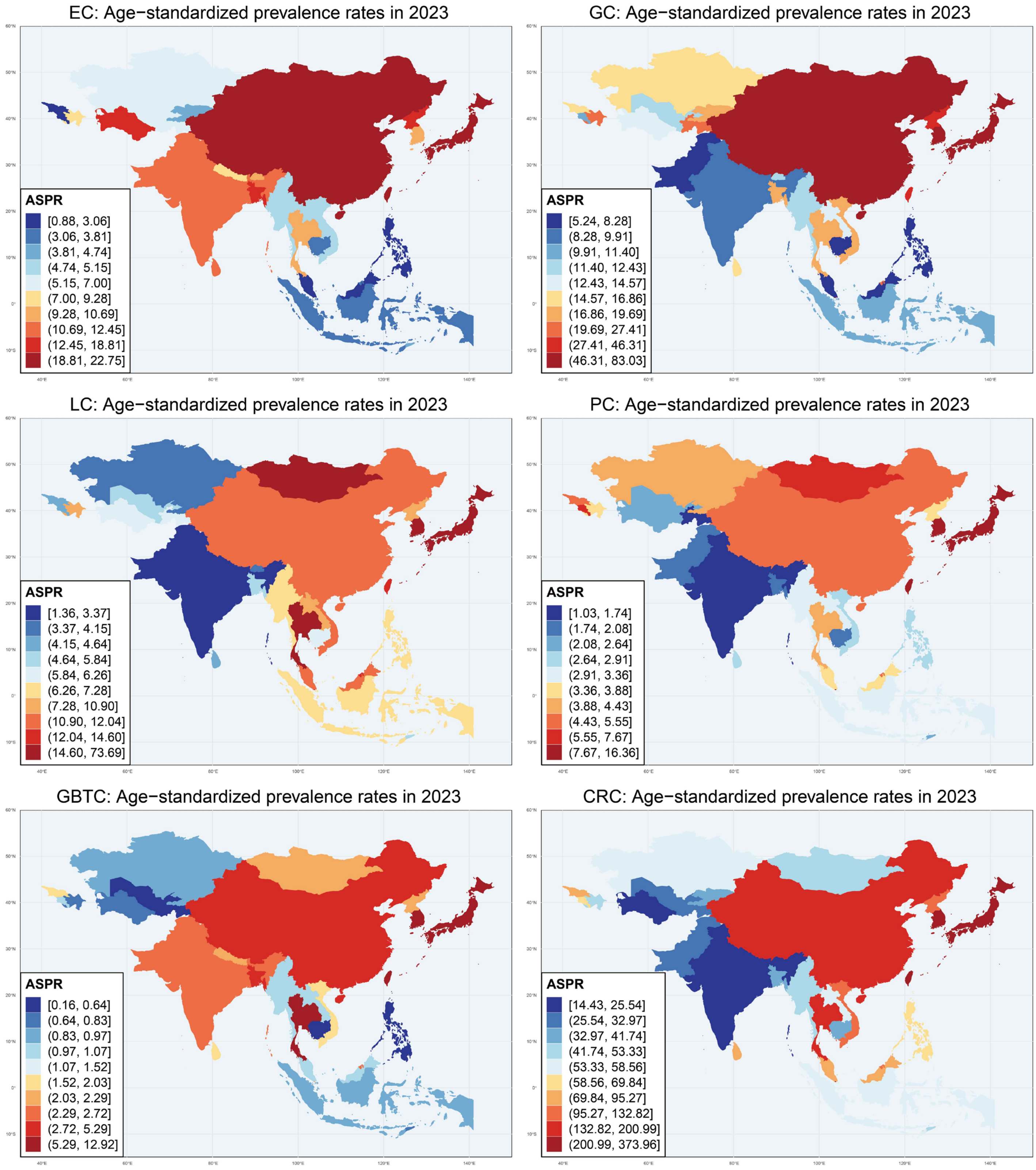

Figure S2: Geographical variation in ASPR for 6 major digestive system cancers by country and territory, 2023. Rates are shown per 100,000 population. (**A**) Esophageal cancer (EC). (**B**) Gastric cancer (GC). (**C**) Liver cancer (LC). (**D**) Pancreatic cancer (PC). (**E**) Gallbladder and biliary tract cancer (GBTC). (**F**) Colorectal cancer (CRC). ASPR, age-standardized prevalence rates.

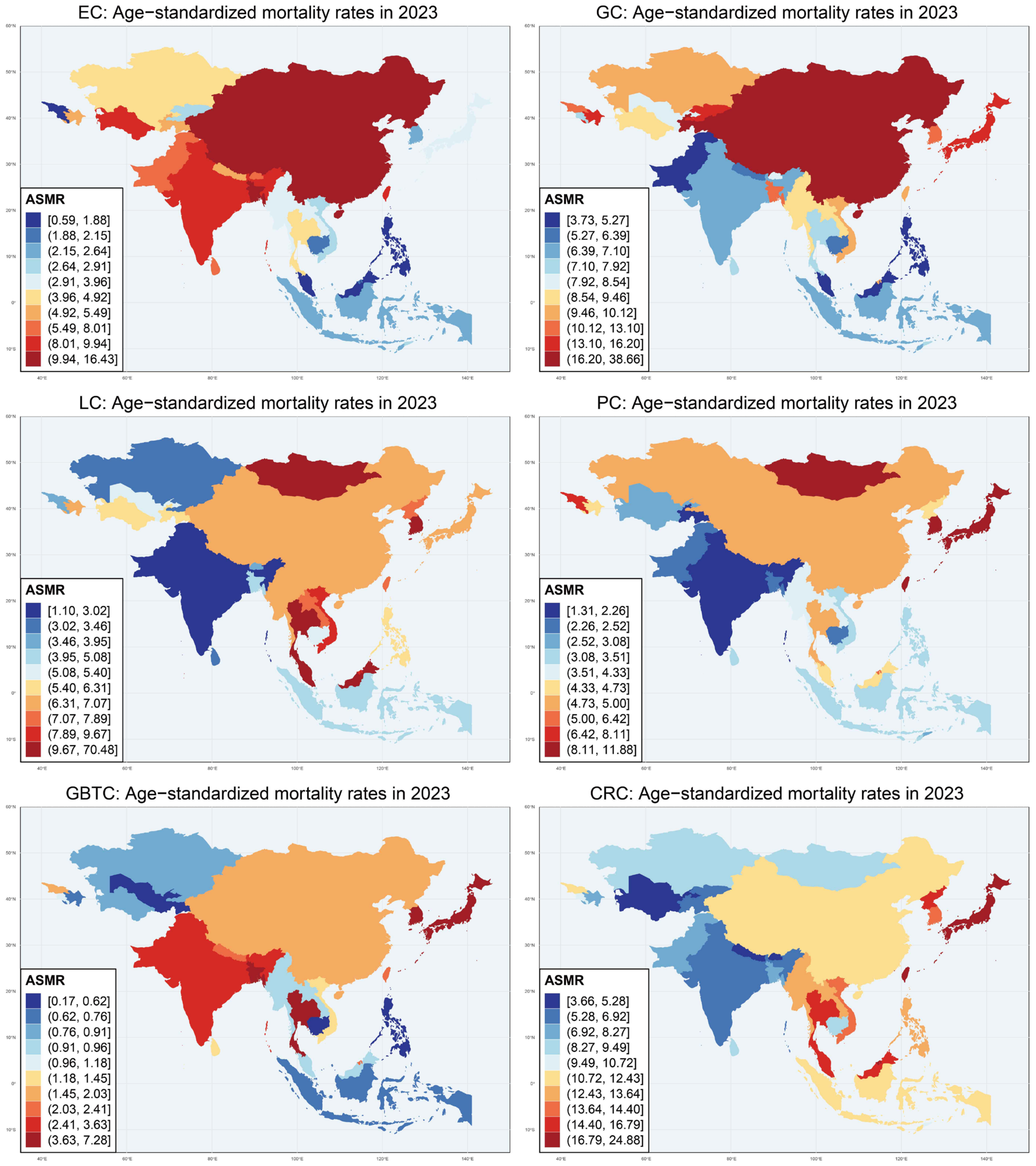

Figure S3: Geographical variation in ASMR for 6 major digestive system cancers by country and territory, 2023. Rates are shown per 100,000 population. (**A**) Esophageal cancer (EC). (**B**) Gastric cancer (GC). (**C**) Liver cancer (LC). (**D**) Pancreatic cancer (PC). (**E**) Gallbladder and biliary tract cancer (GBTC). (**F**) Colorectal cancer (CRC). ASMR, age-standardized mortality rates.

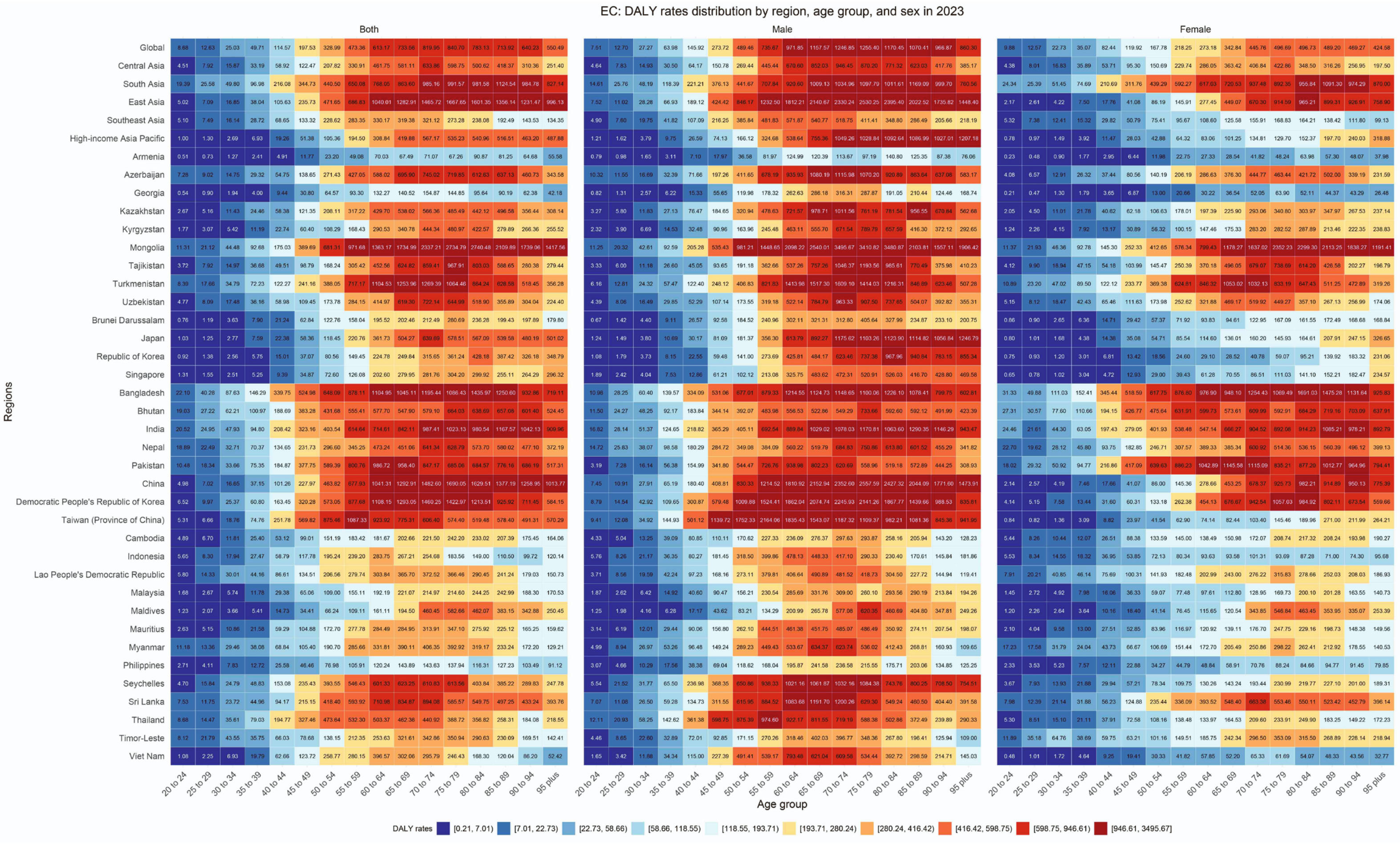

Figure S4: Distribution of esophageal cancer (EC) disability-adjusted life year (DALY) rates by age group, geographic region, and sex, 2023.

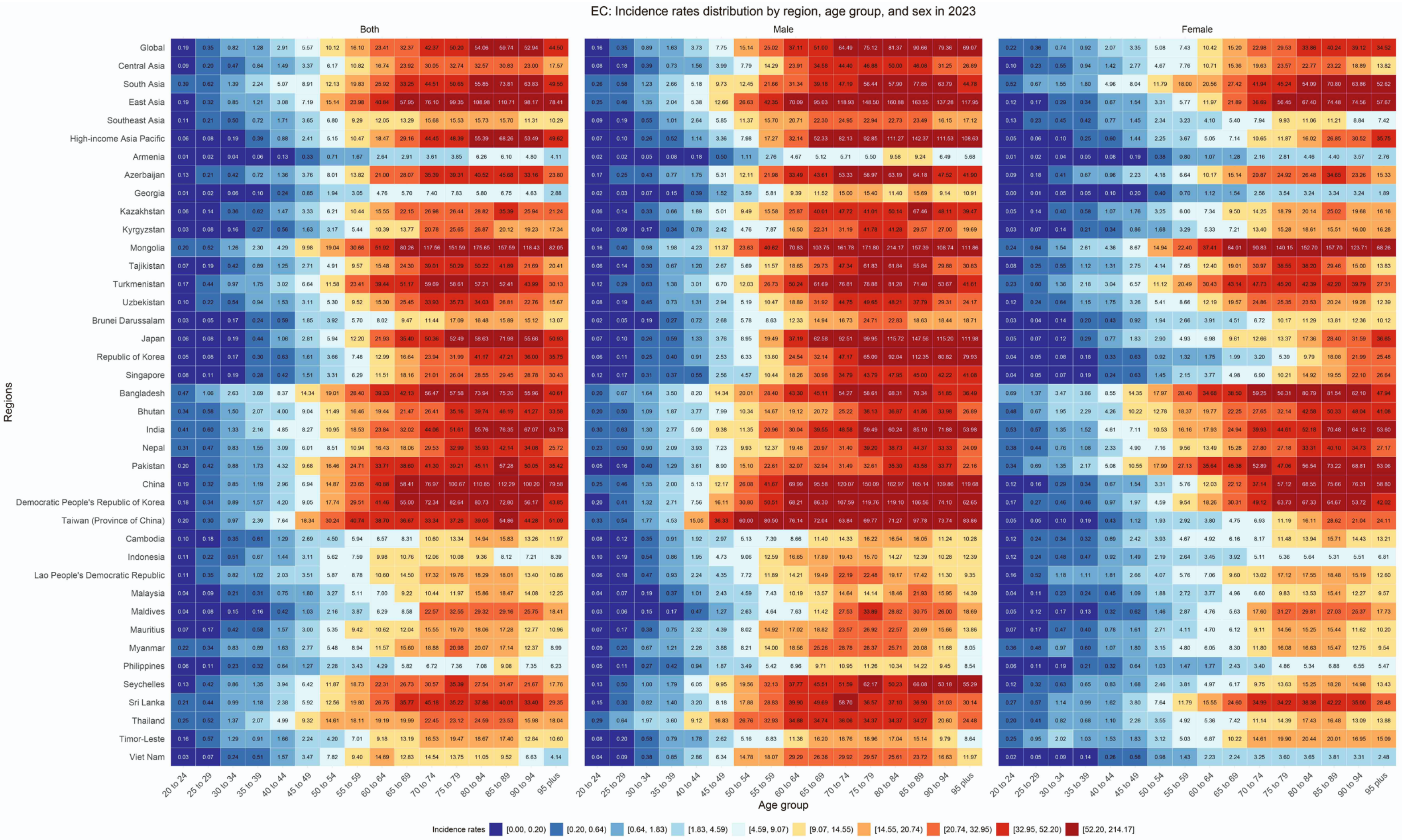

Figure S5: Distribution of esophageal cancer (EC) incidence rates by age group, geographic region, and sex, 2023.

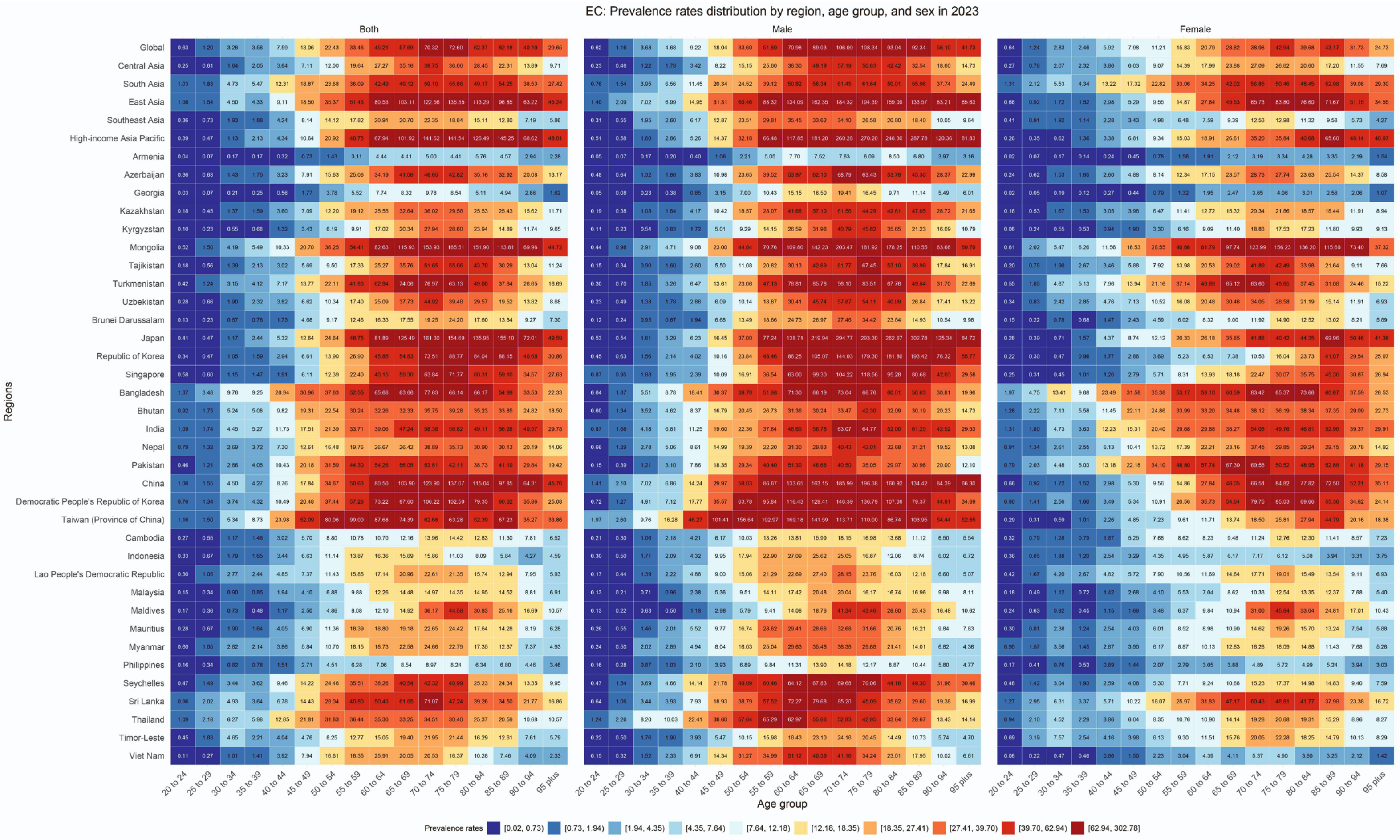

Figure S6: Distribution of esophageal cancer (EC) prevalence rates by age group, geographic region, and sex, 2023.

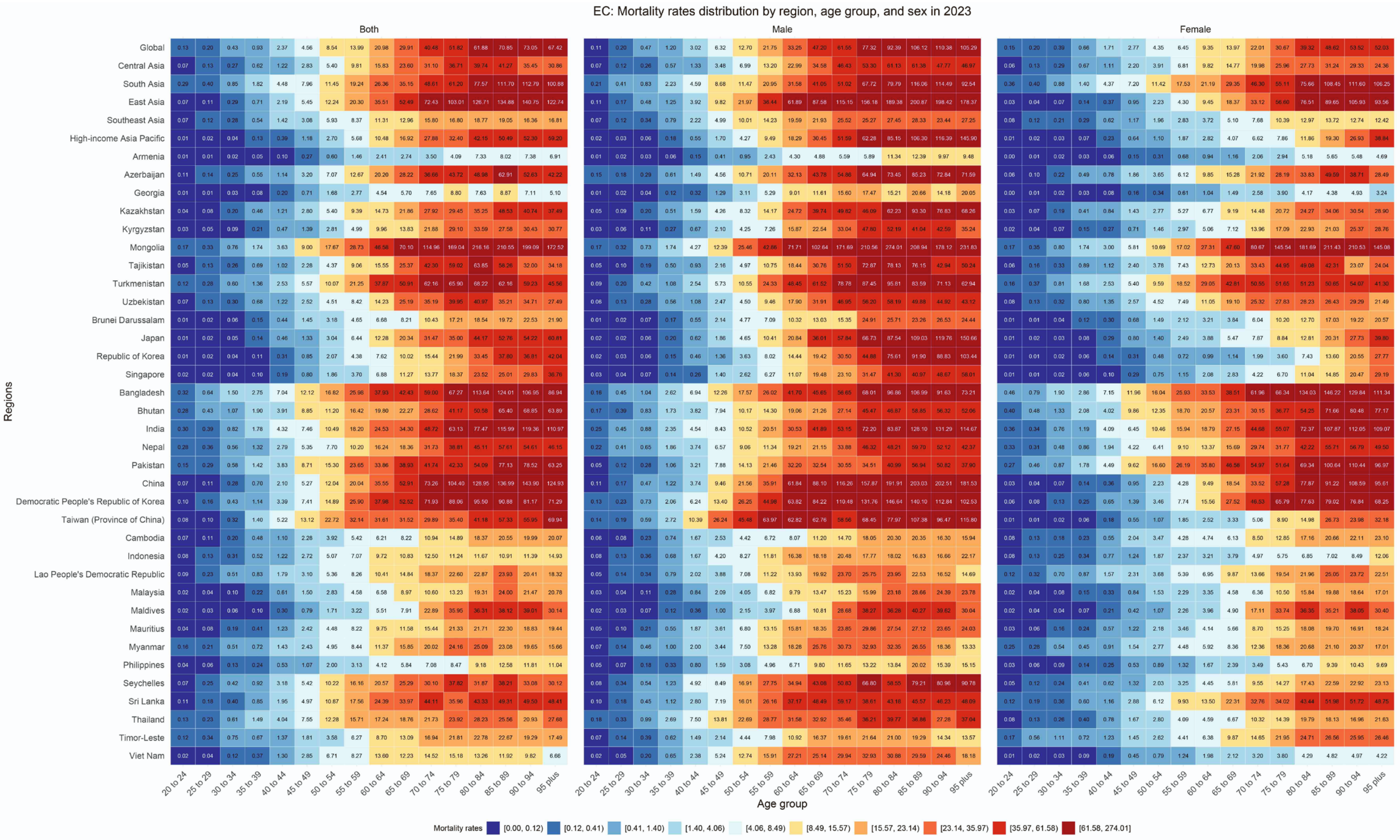

Figure S7: Distribution of esophageal cancer (EC) mortality rates by age group, geographic region, and sex, 2023.

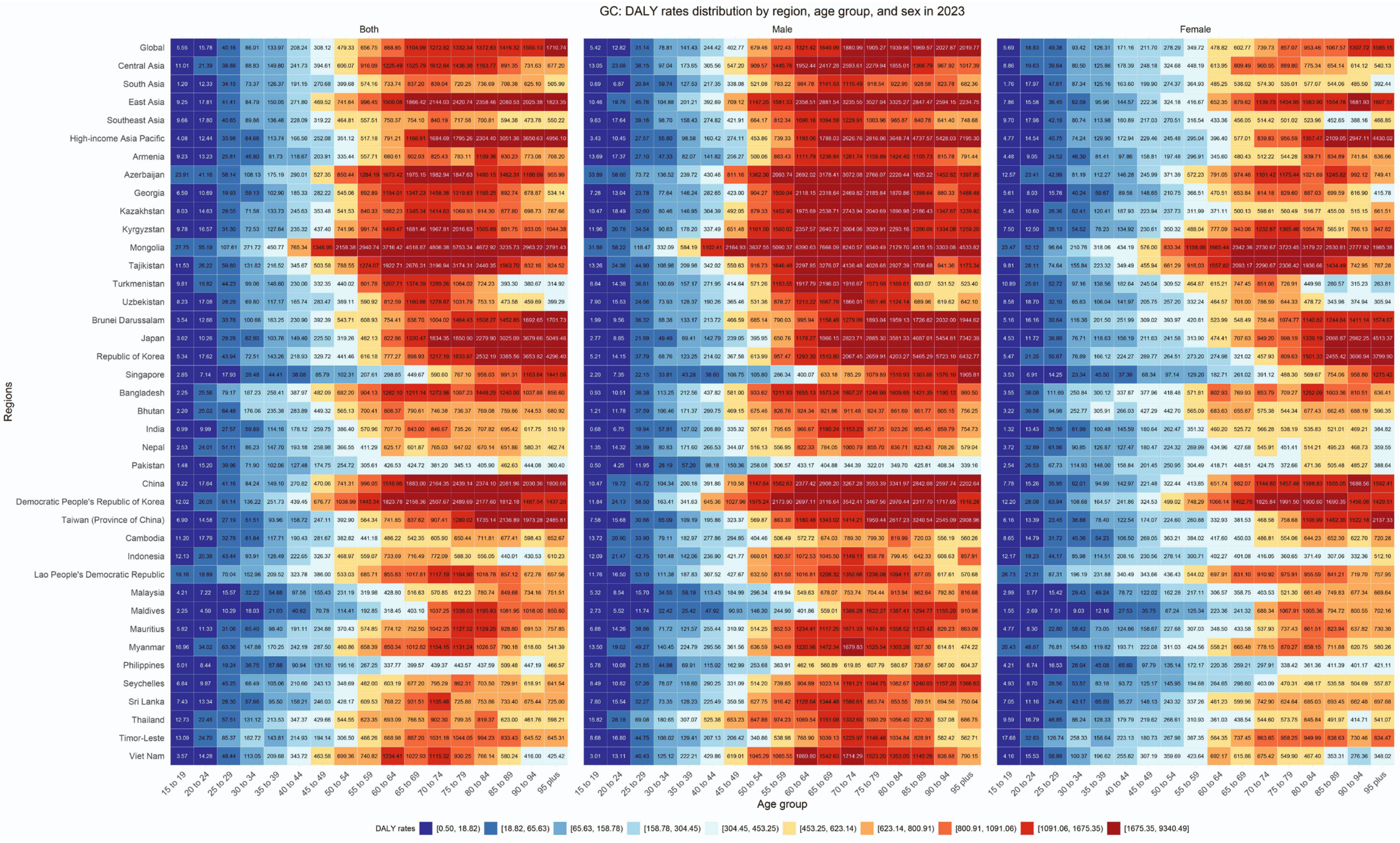

Figure S8: Distribution of gastric cancer (GC) disability-adjusted life year (DALY) rates by age group, geographic region, and sex, 2023.

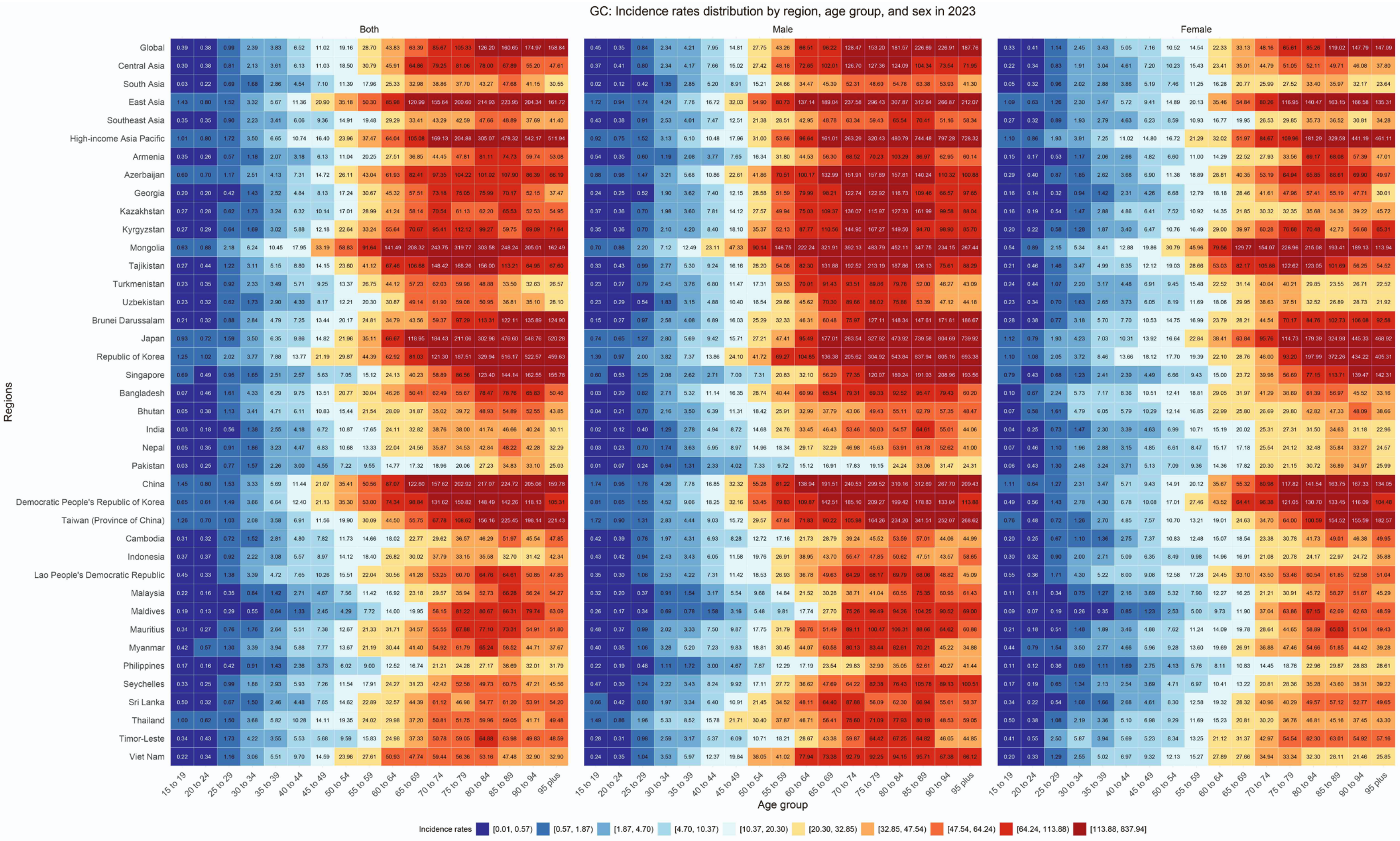

Figure S9: Distribution of gastric cancer (GC) incidence rates by age group, geographic region, and sex, 2023.

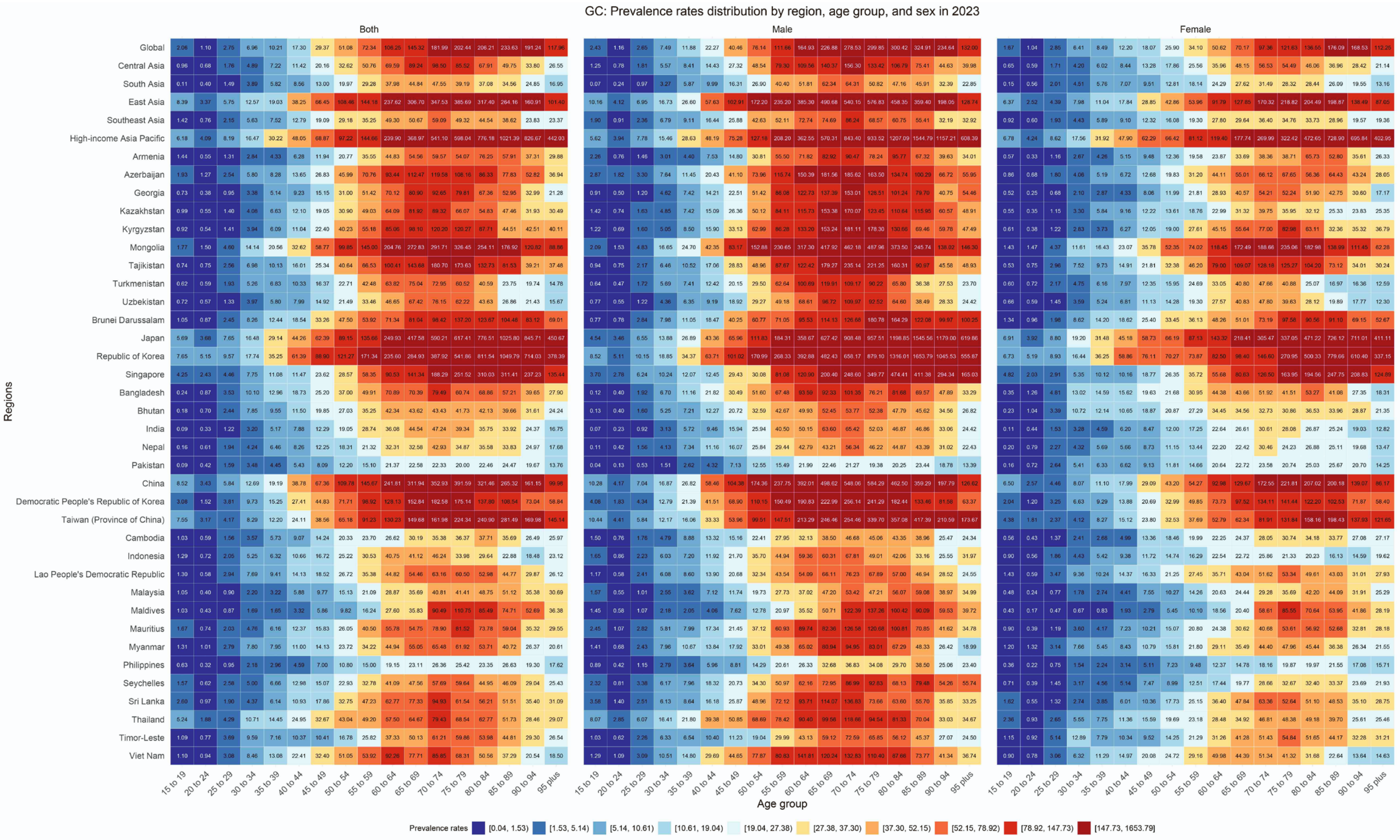

Figure S10: Distribution of gastric cancer (GC) prevalence rates by age group, geographic region, and sex, 2023.

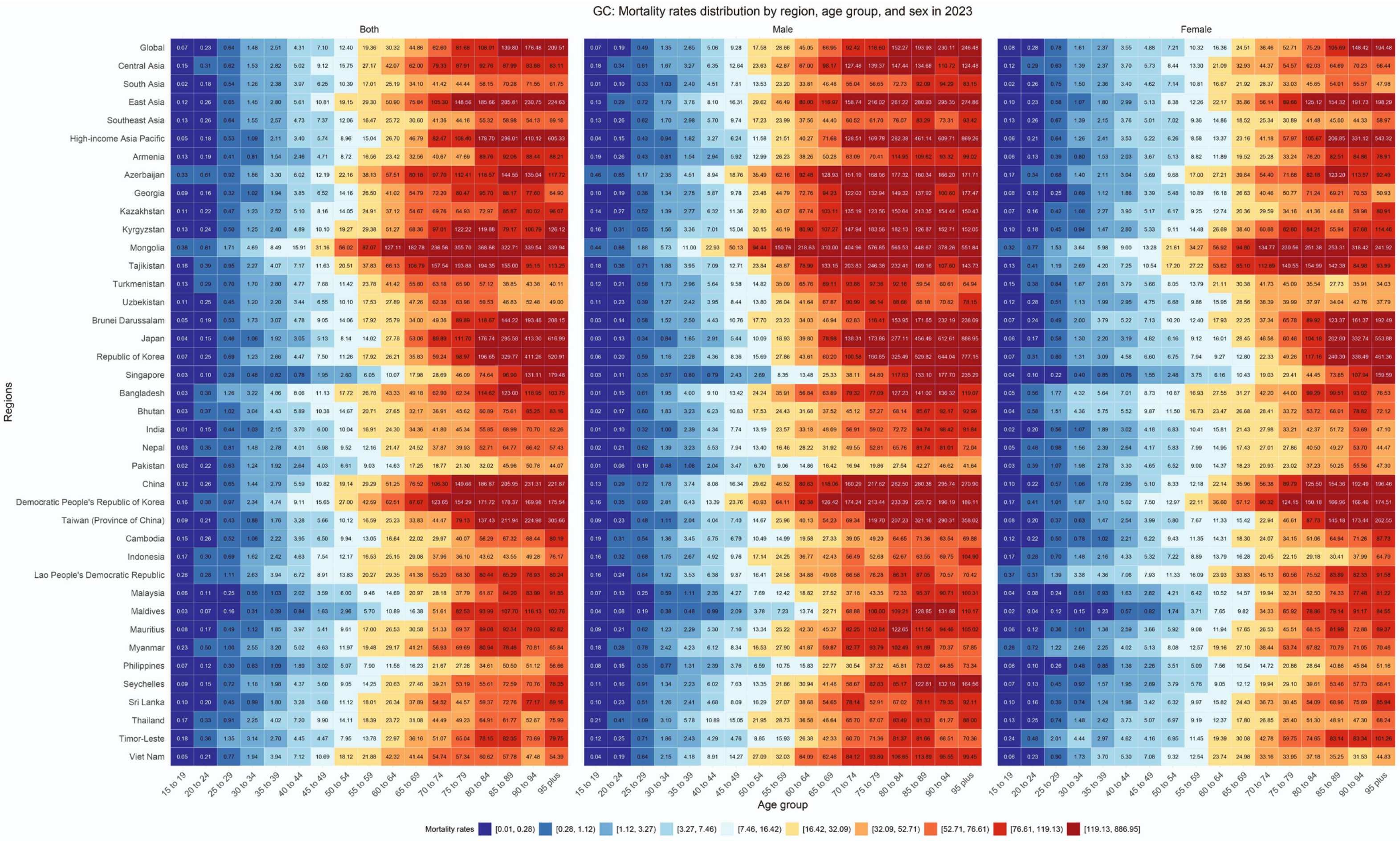

Figure S11: Distribution of gastric cancer (GC) mortality rates by age group, geographic region, and sex, 2023.

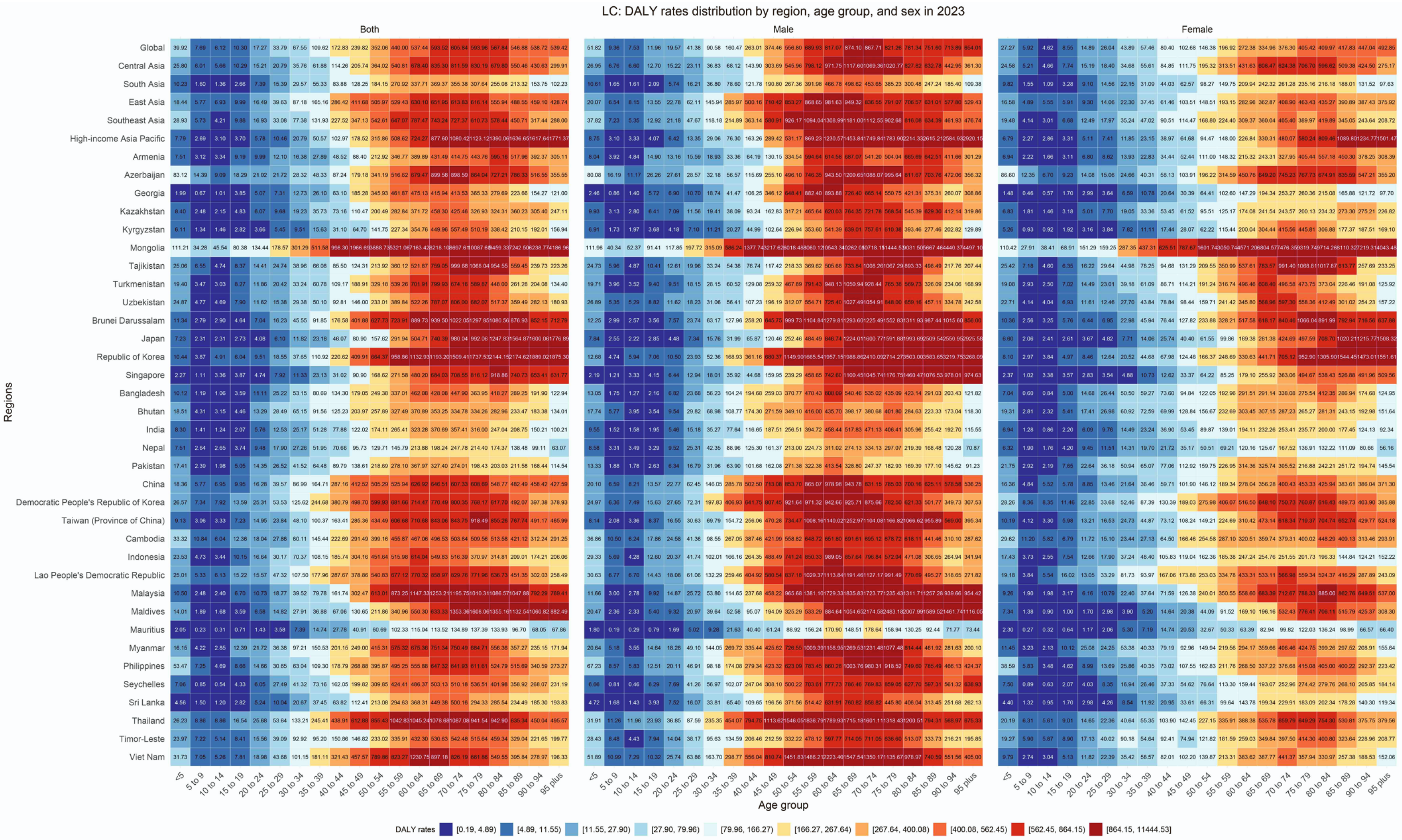

Figure S12: Distribution of liver cancer (LC) disability-adjusted life year (DALY) rates by age group, geographic region, and sex, 2023.

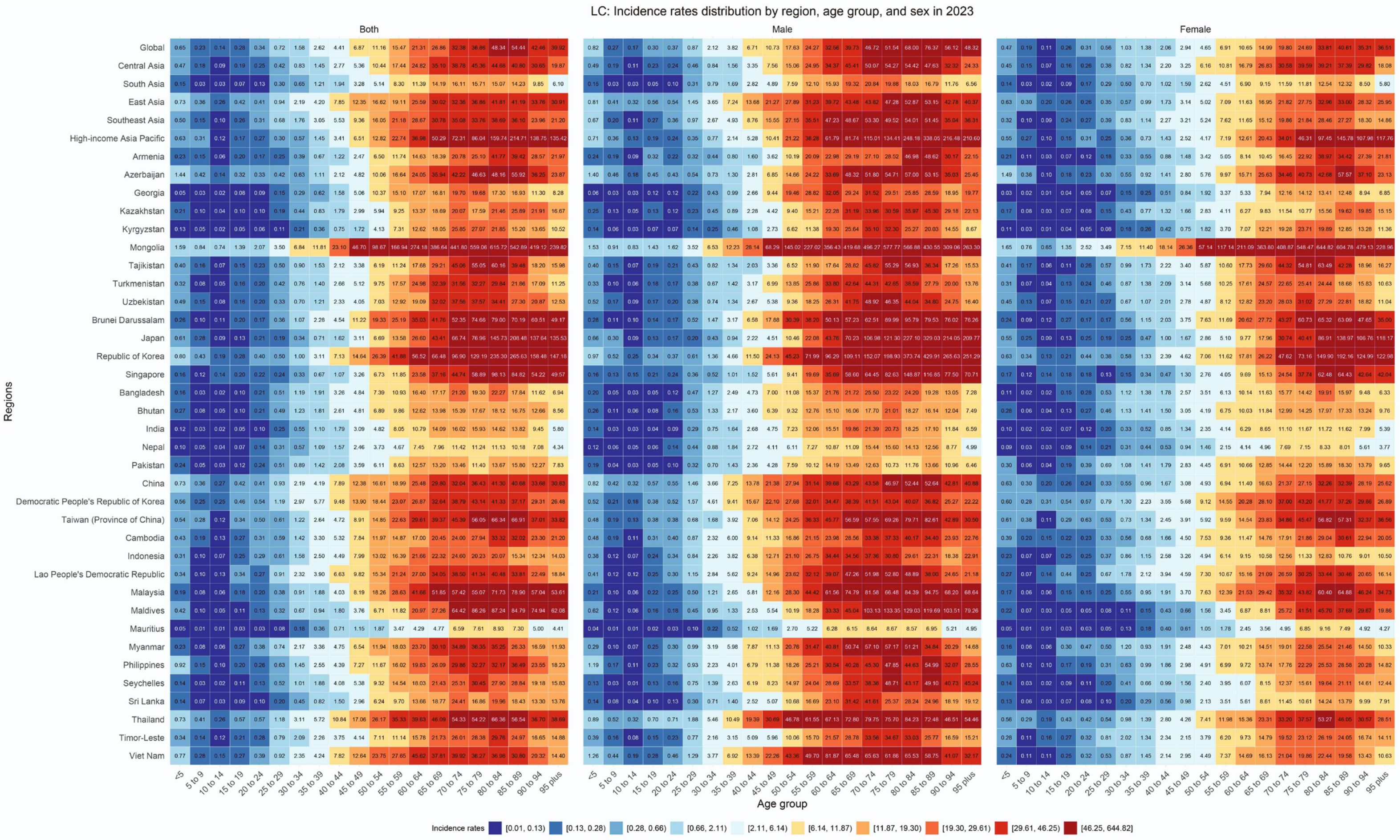

Figure S13: Distribution of liver cancer (LC) incidence rates by age group, geographic region, and sex, 2023.

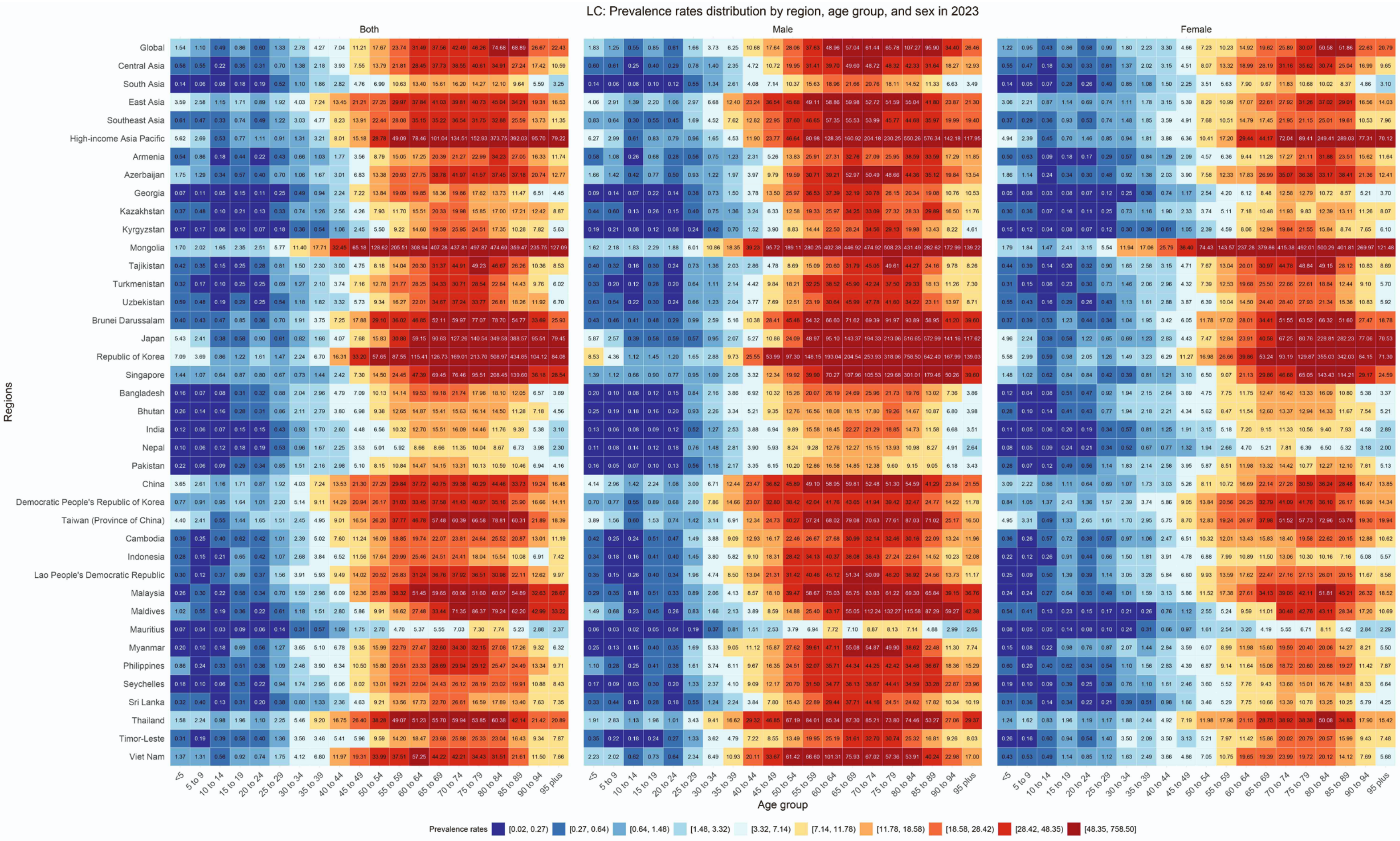

Figure S14: Distribution of liver cancer (LC) prevalence rates by age group, geographic region, and sex, 2023.

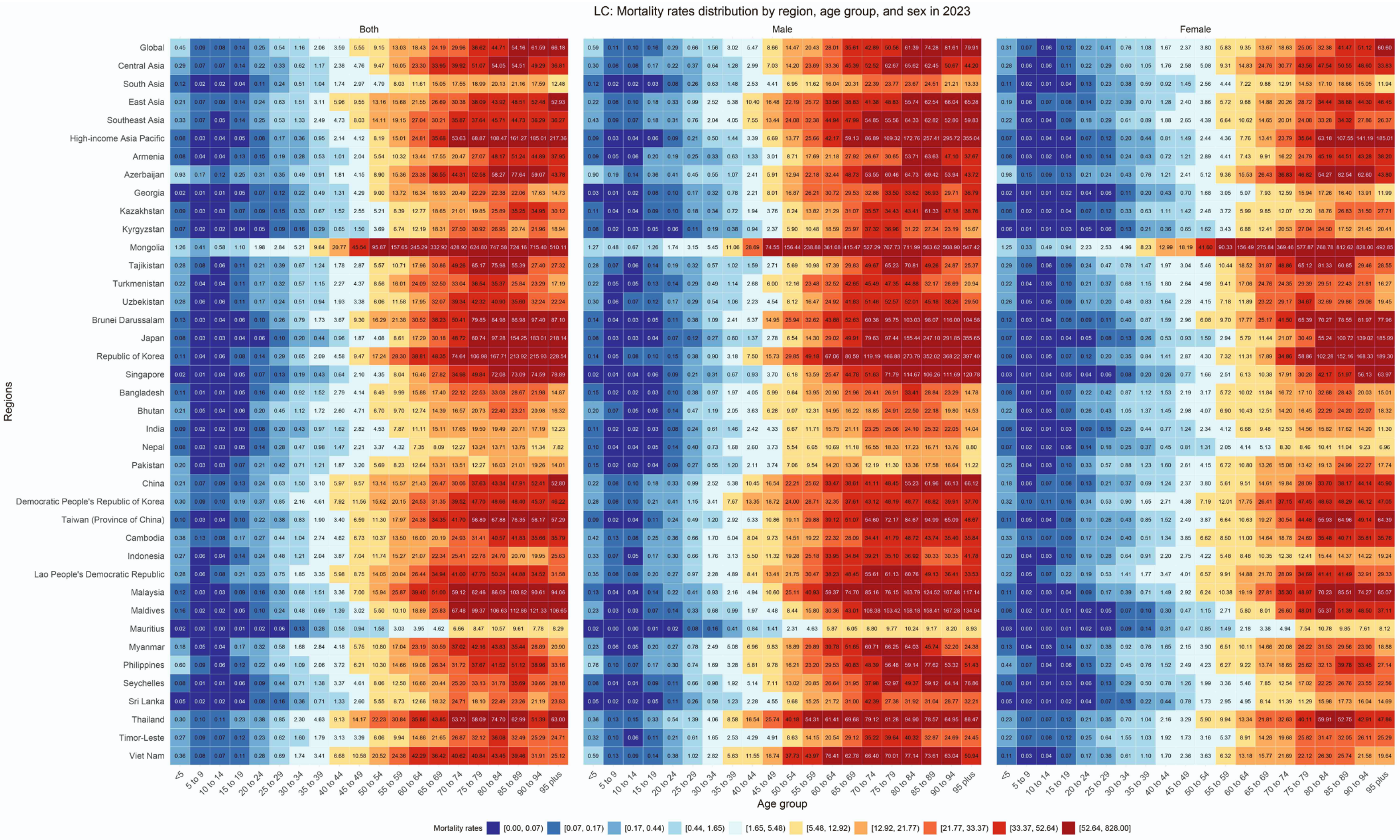

Figure S15: Distribution of liver cancer (LC) mortality rates by age group, geographic region, and sex, 2023.

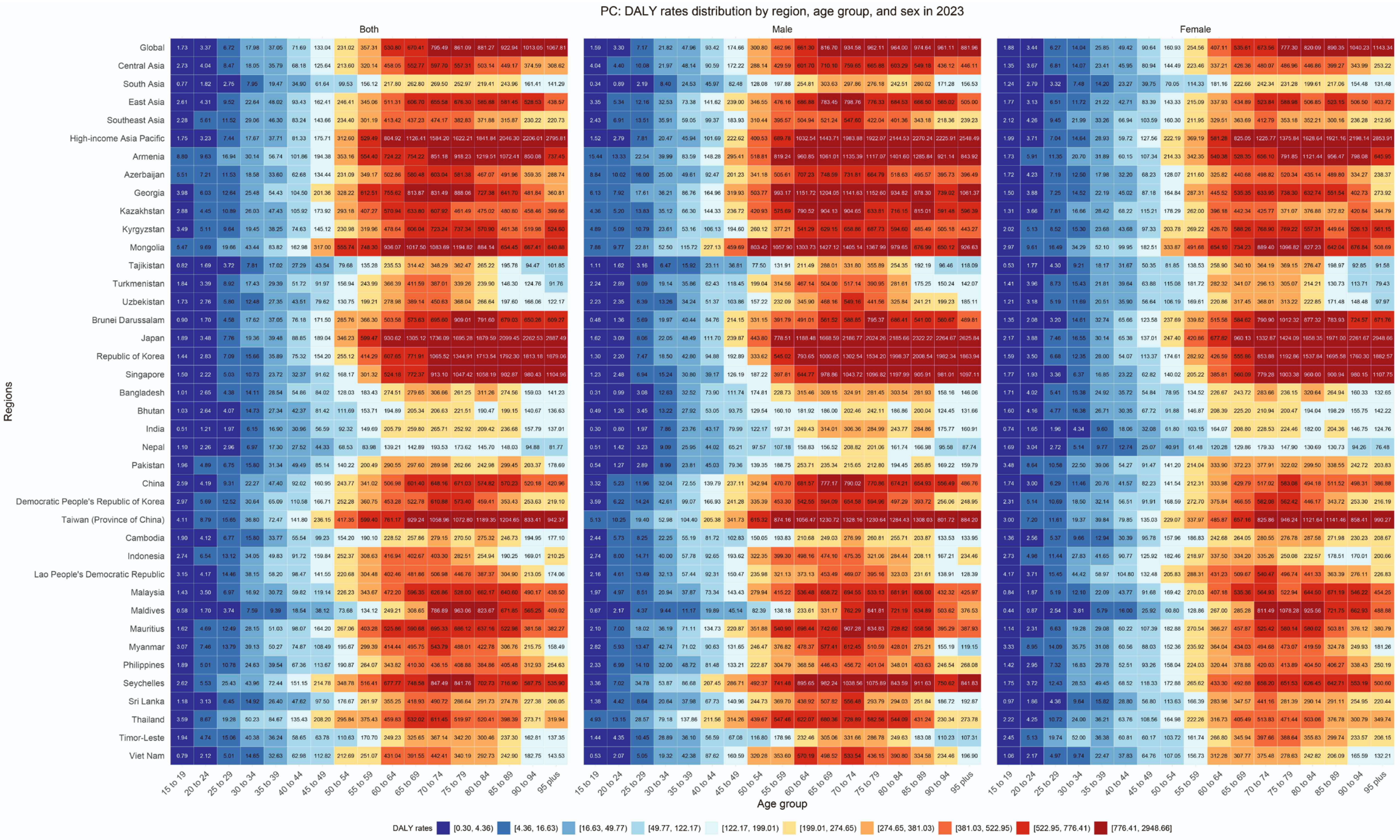

Figure S16: Distribution of pancreatic cancer (PC) disability-adjusted life year (DALY) rates by age group, geographic region, and sex, 2023.

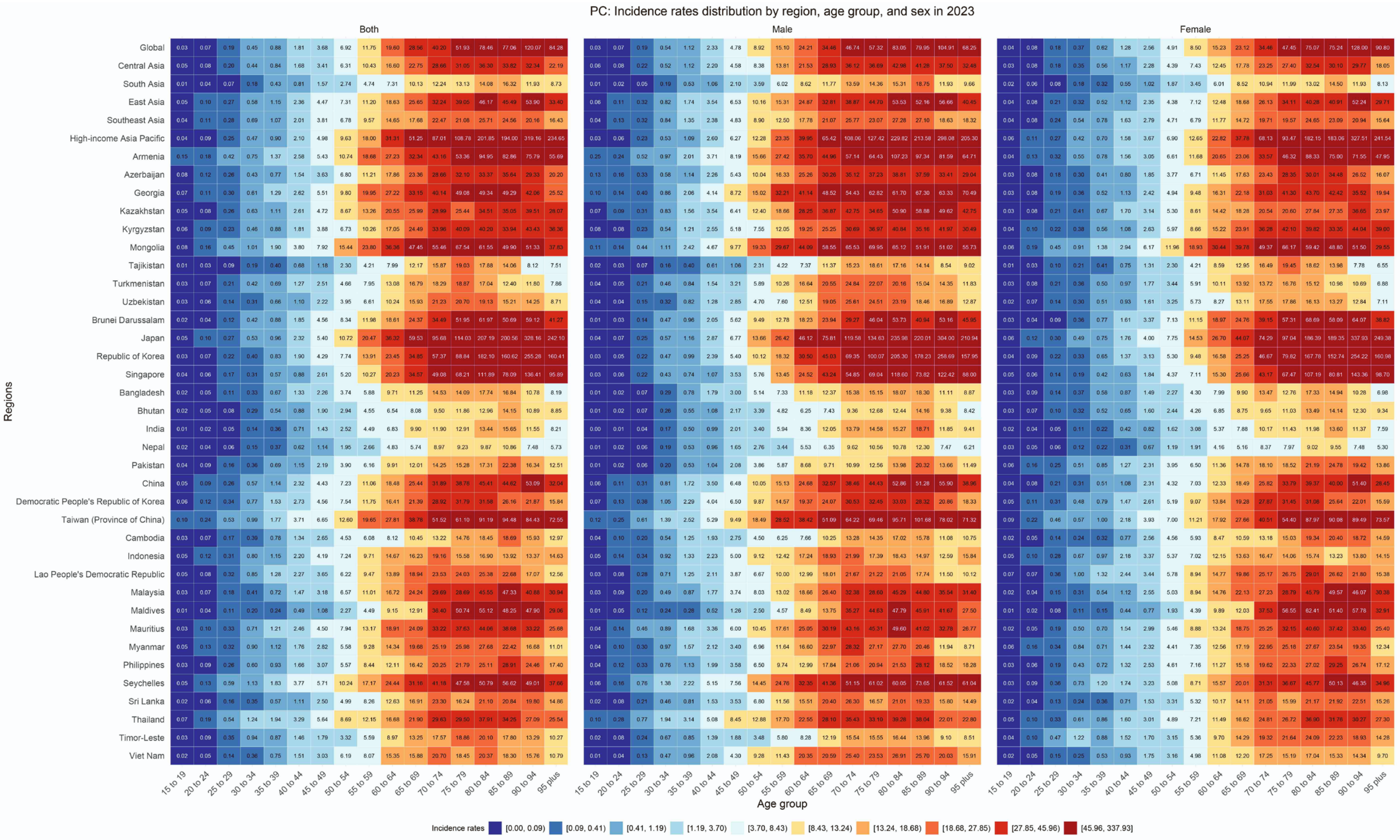

Figure S17: Distribution of pancreatic cancer (PC) incidence rates by age group, geographic region, and sex, 2023.

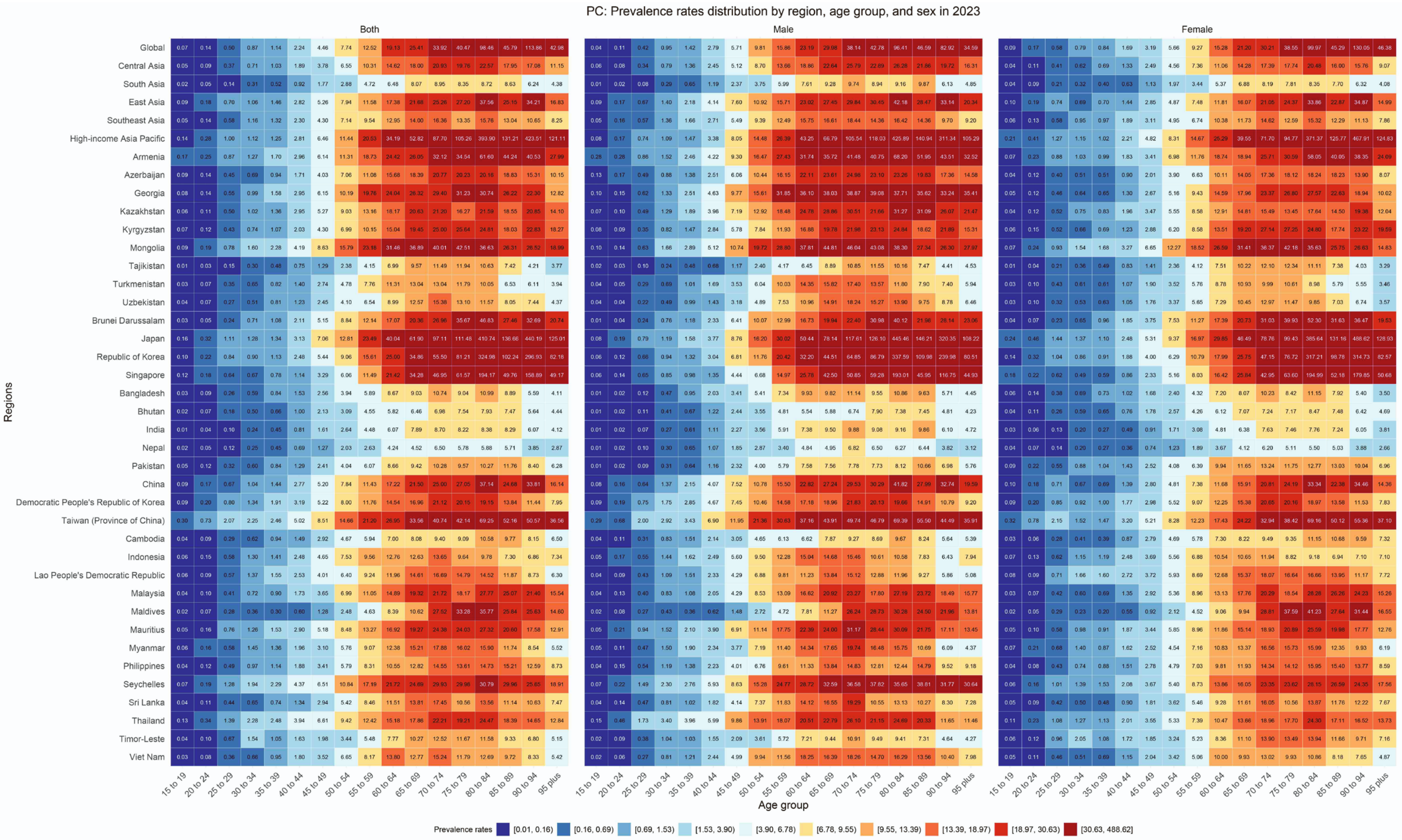

Figure S18: Distribution of pancreatic cancer (PC) prevalence rates by age group, geographic region, and sex, 2023.

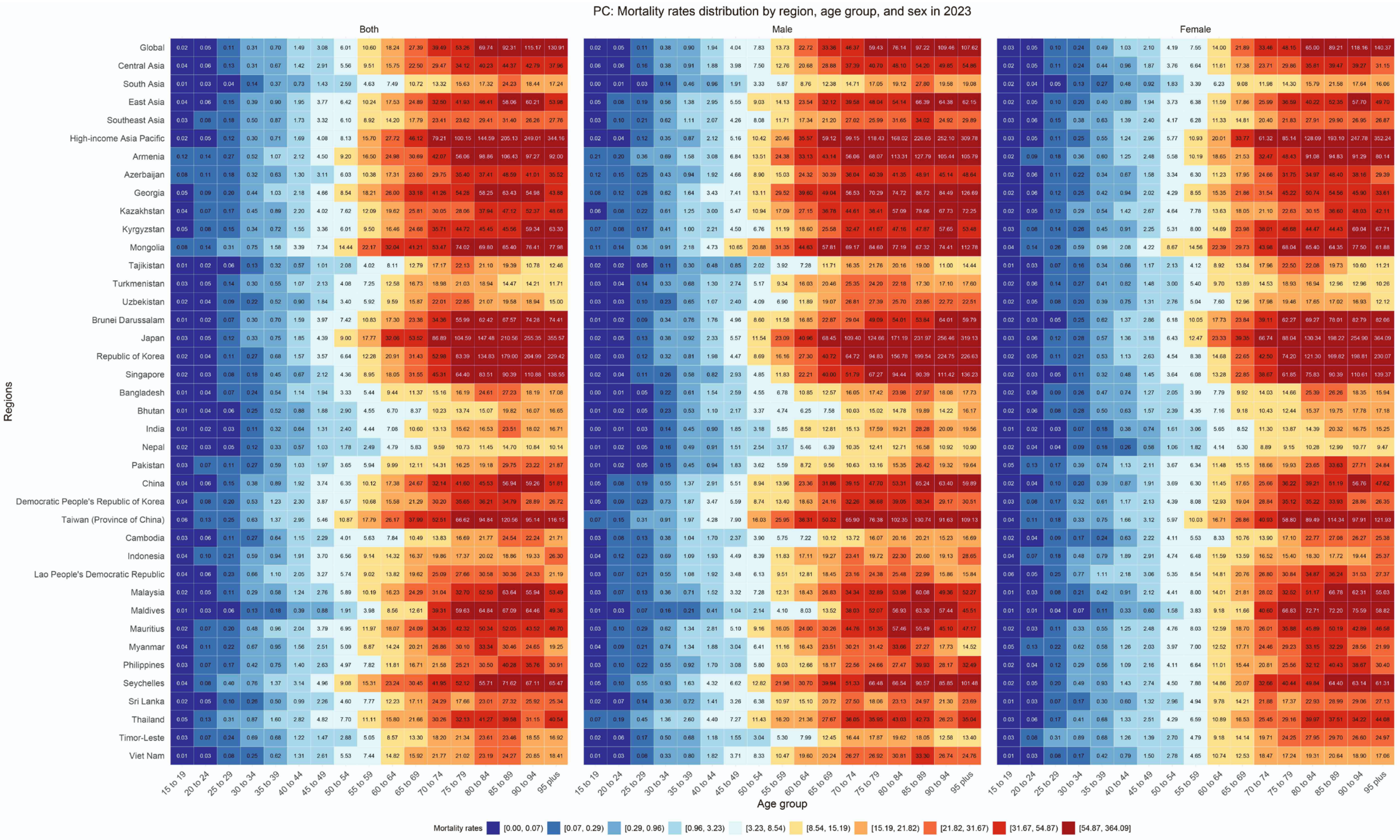

Figure S19: Distribution of pancreatic cancer (PC) mortality rates by age group, geographic region, and sex, 2023.

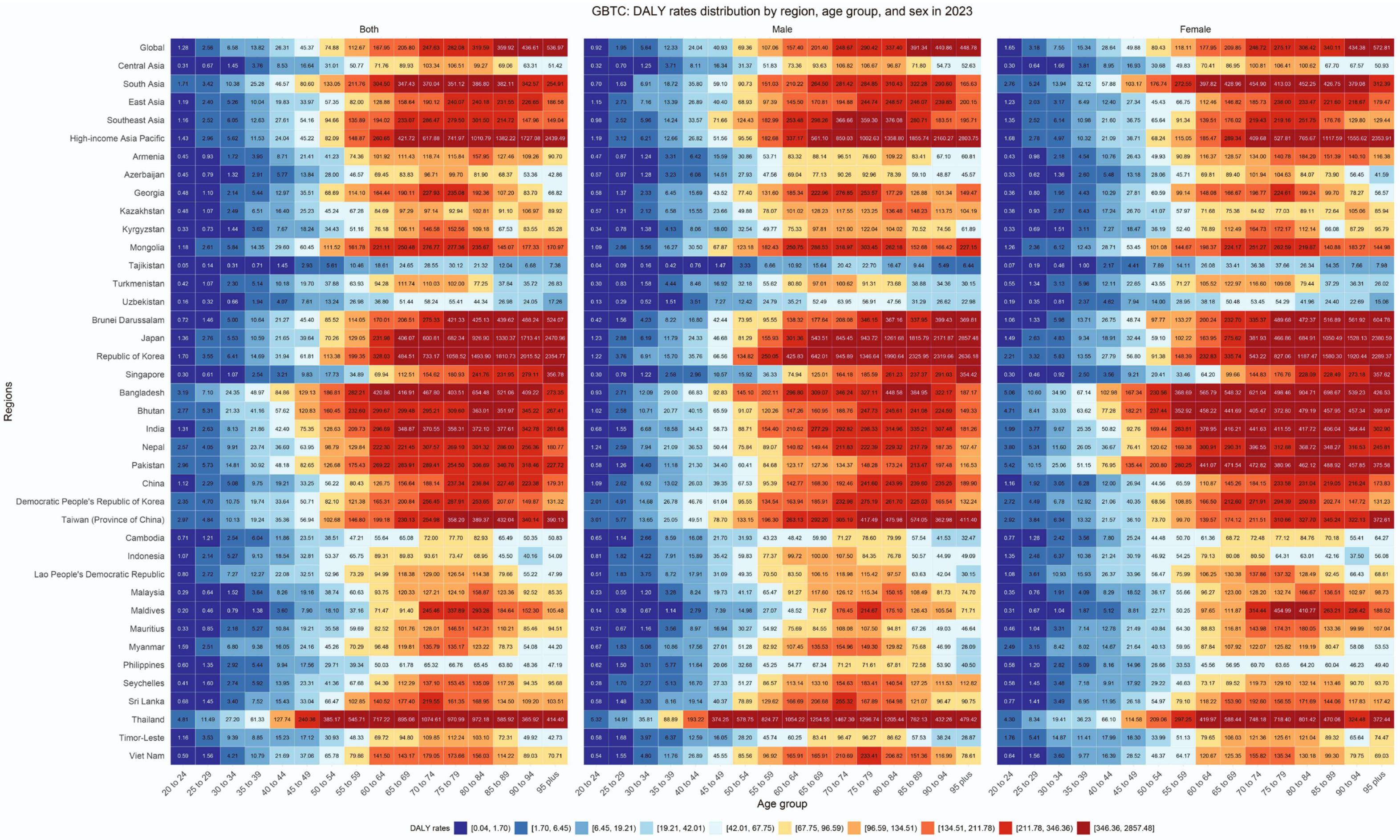

Figure S20: Distribution of gallbladder and biliary tract cancer (GBTC) disability-adjusted life year (DALY) rates by age group, geographic region, and sex, 2023.

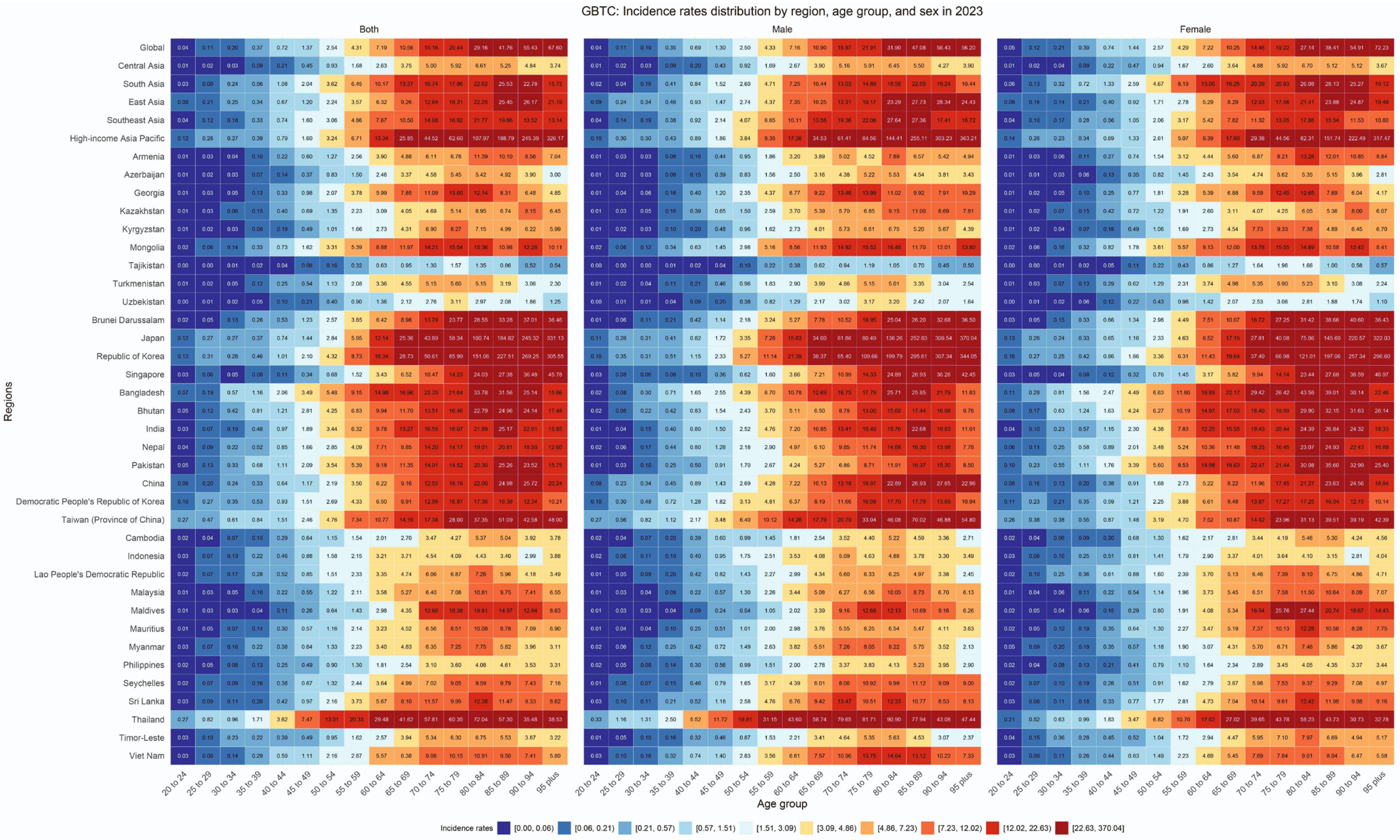

Figure S21: Distribution of gallbladder and biliary tract cancer (GBTC) incidence rates by age group, geographic region, and sex, 2023.

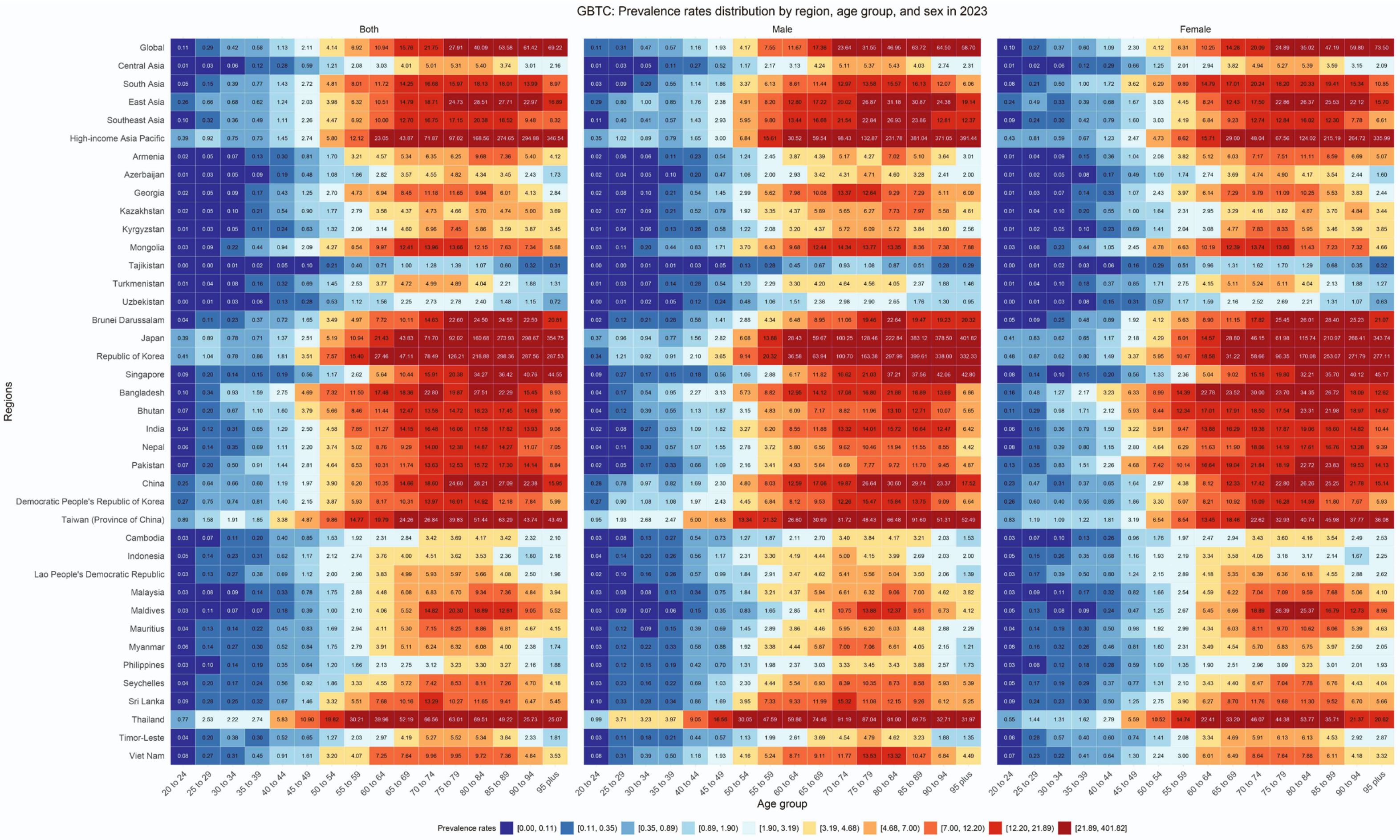

Figure S22: Distribution of gallbladder and biliary tract cancer (GBTC) prevalence rates by age group, geographic region, and sex, 2023.

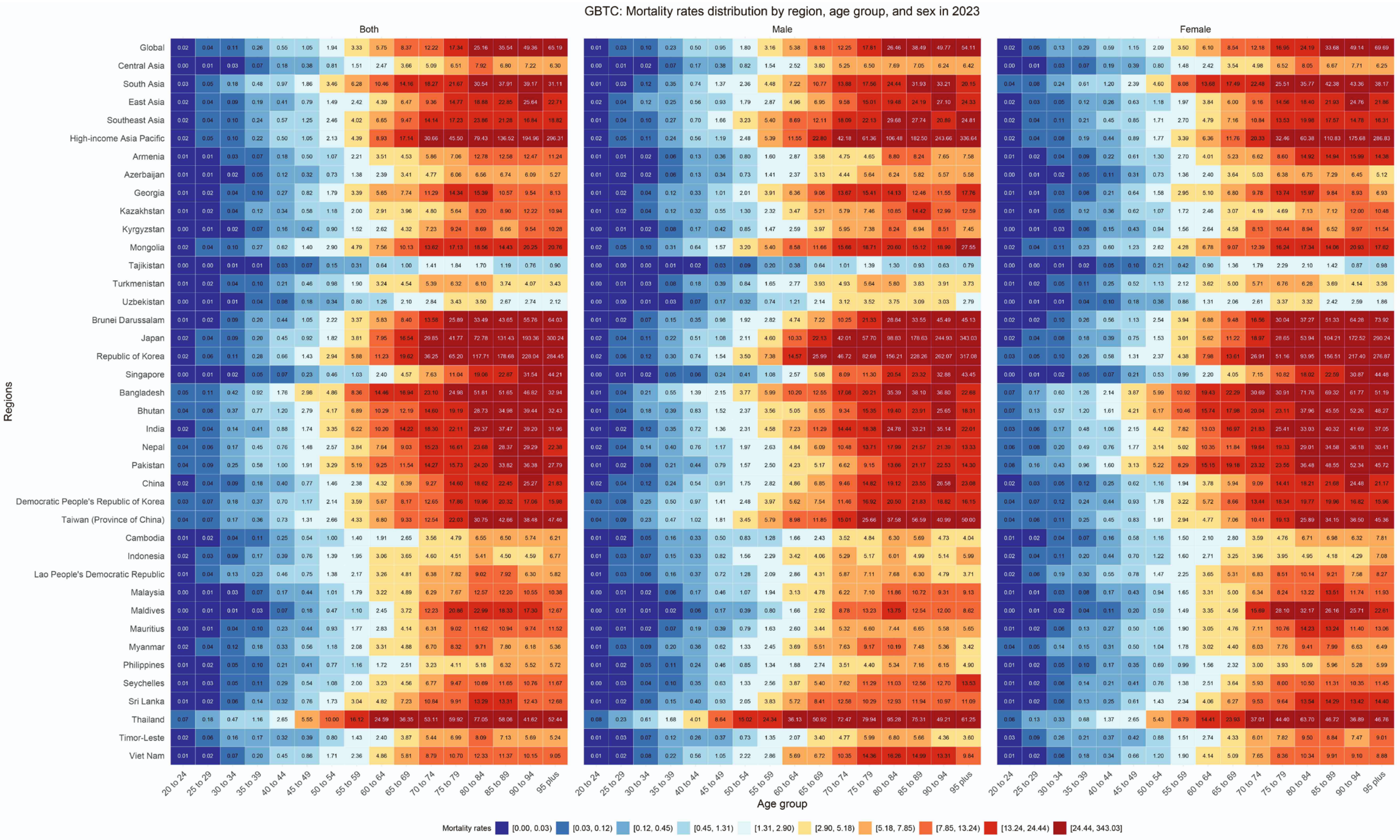

Figure S23: Distribution of gallbladder and biliary tract cancer (GBTC) mortality rates by age group, geographic region, and sex, 2023.

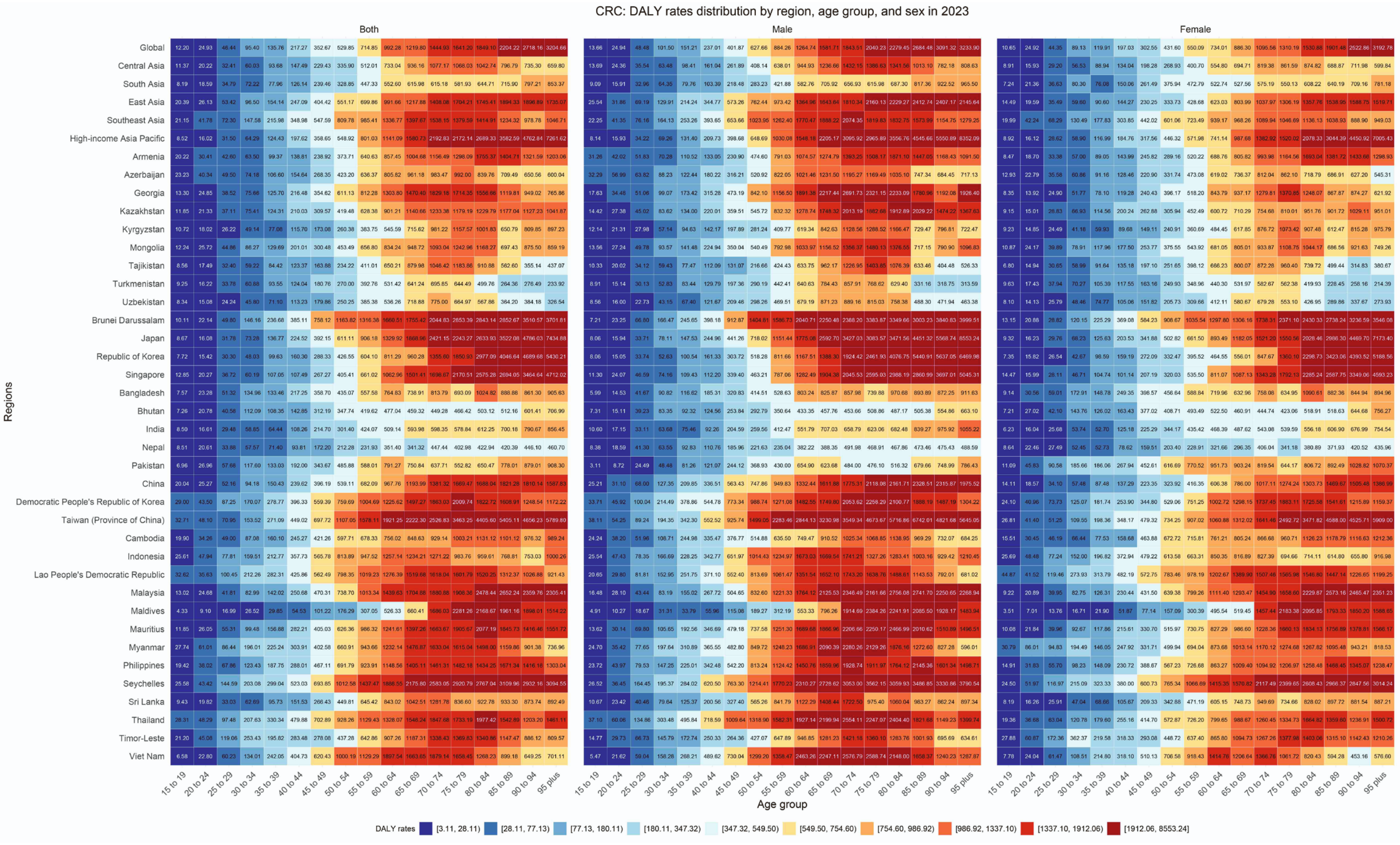

Figure S24: Distribution of colorectal cancer (CRC) disability-adjusted life year (DALY) rates by age group, geographic region, and sex, 2023.

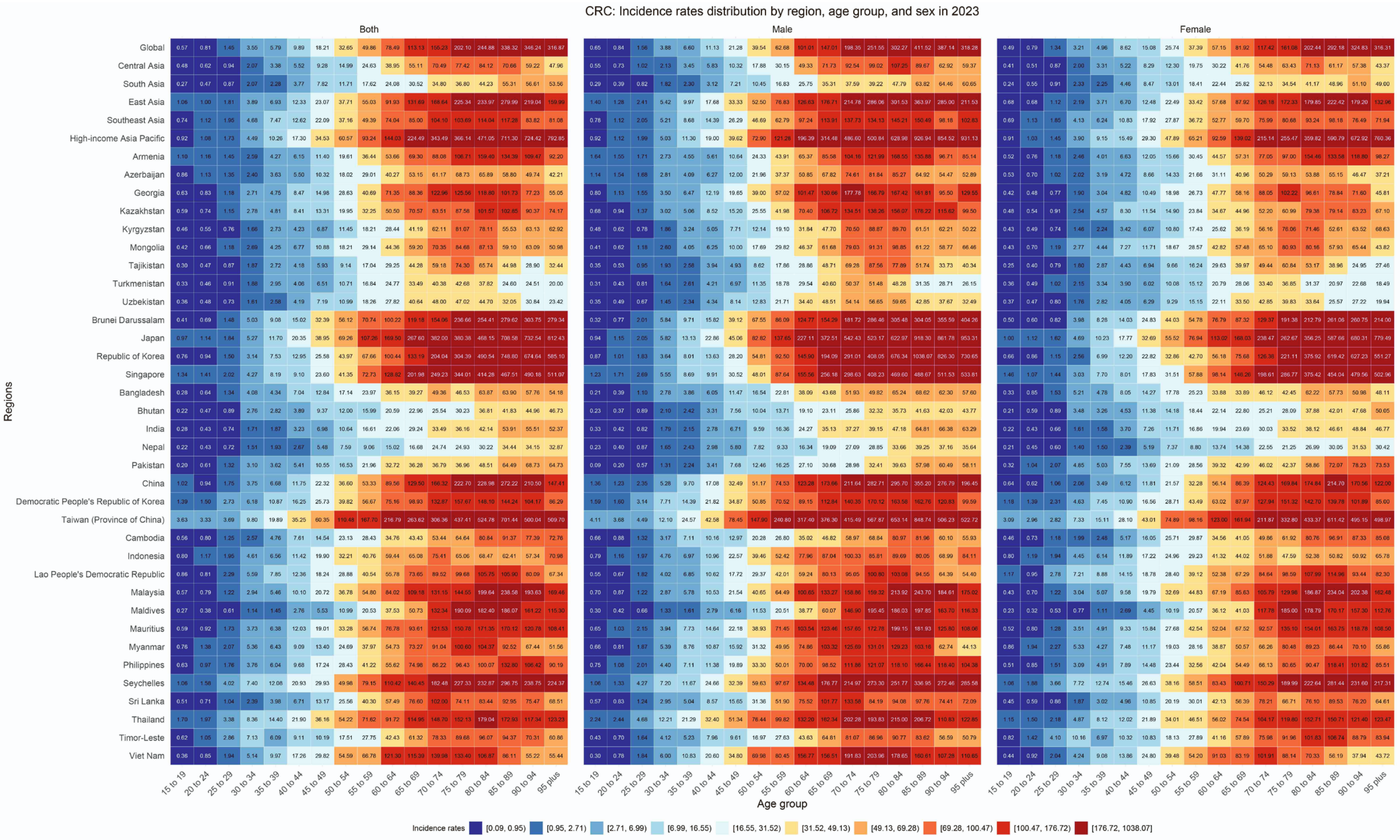

Figure S25: Distribution of colorectal cancer (CRC) incidence rates by age group, geographic region, and sex, 2023.

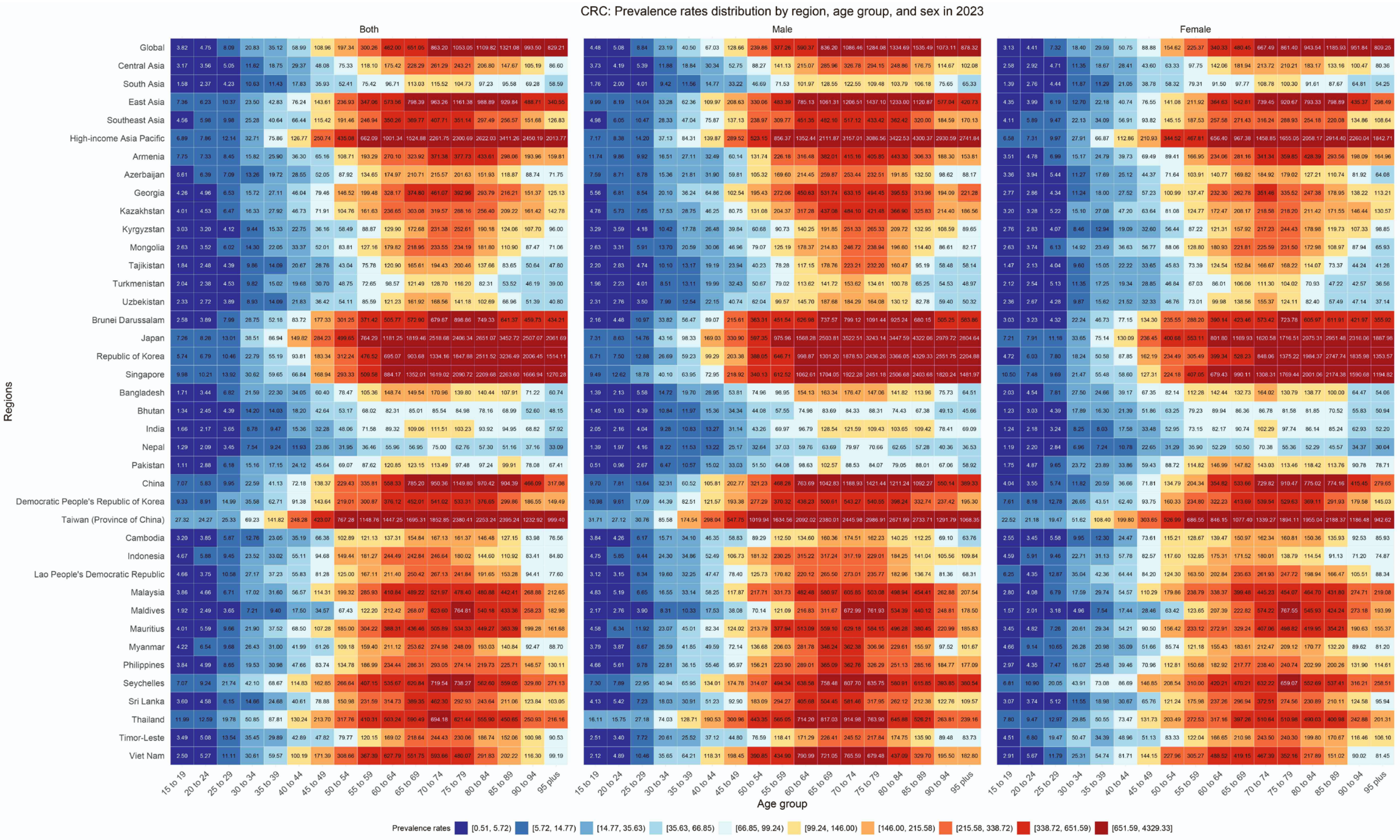

Figure S26: Distribution of colorectal cancer (CRC) prevalence rates by age group, geographic region, and sex, 2023.

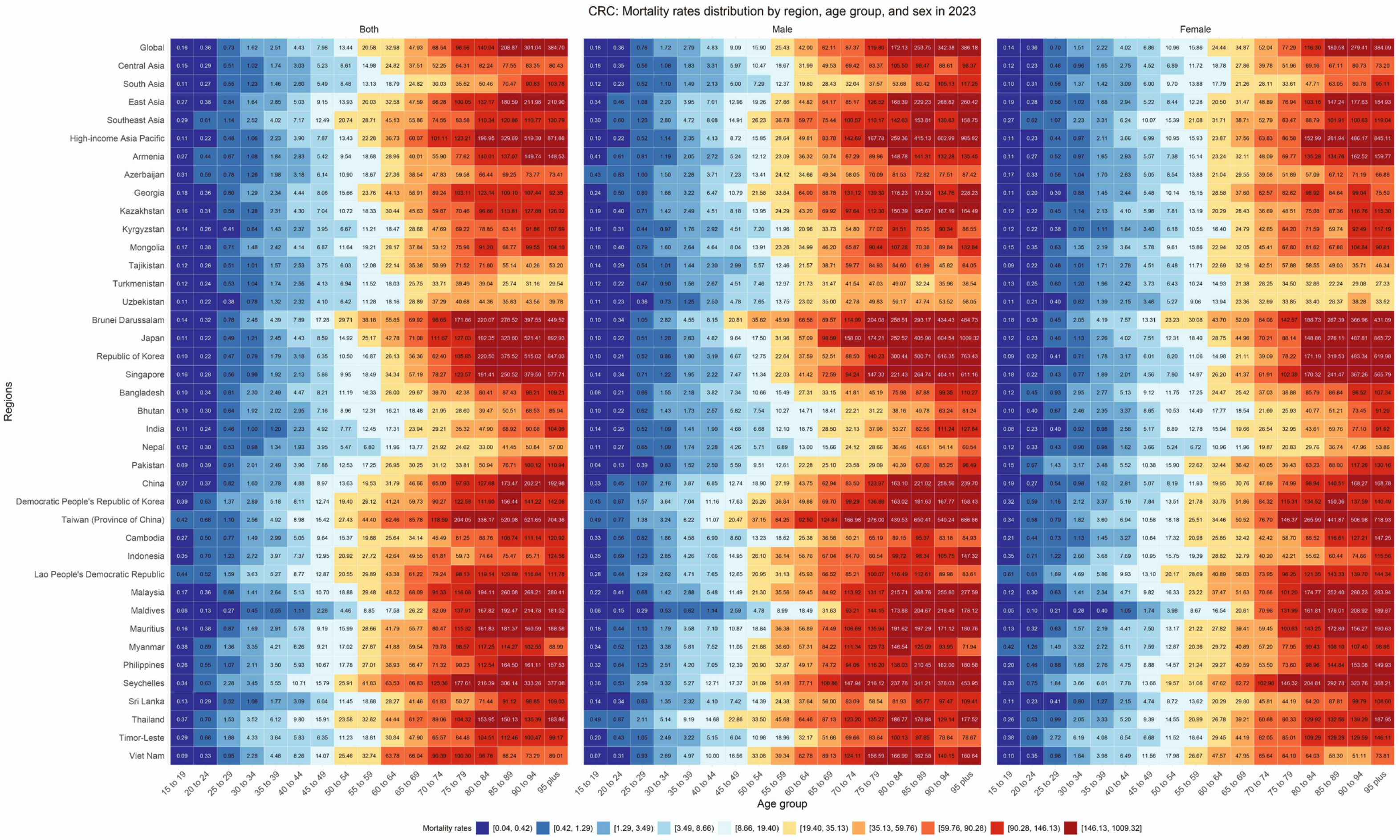

Figure S27: Distribution of colorectal cancer (CRC) mortality rates by age group, geographic region, and sex, 2023.

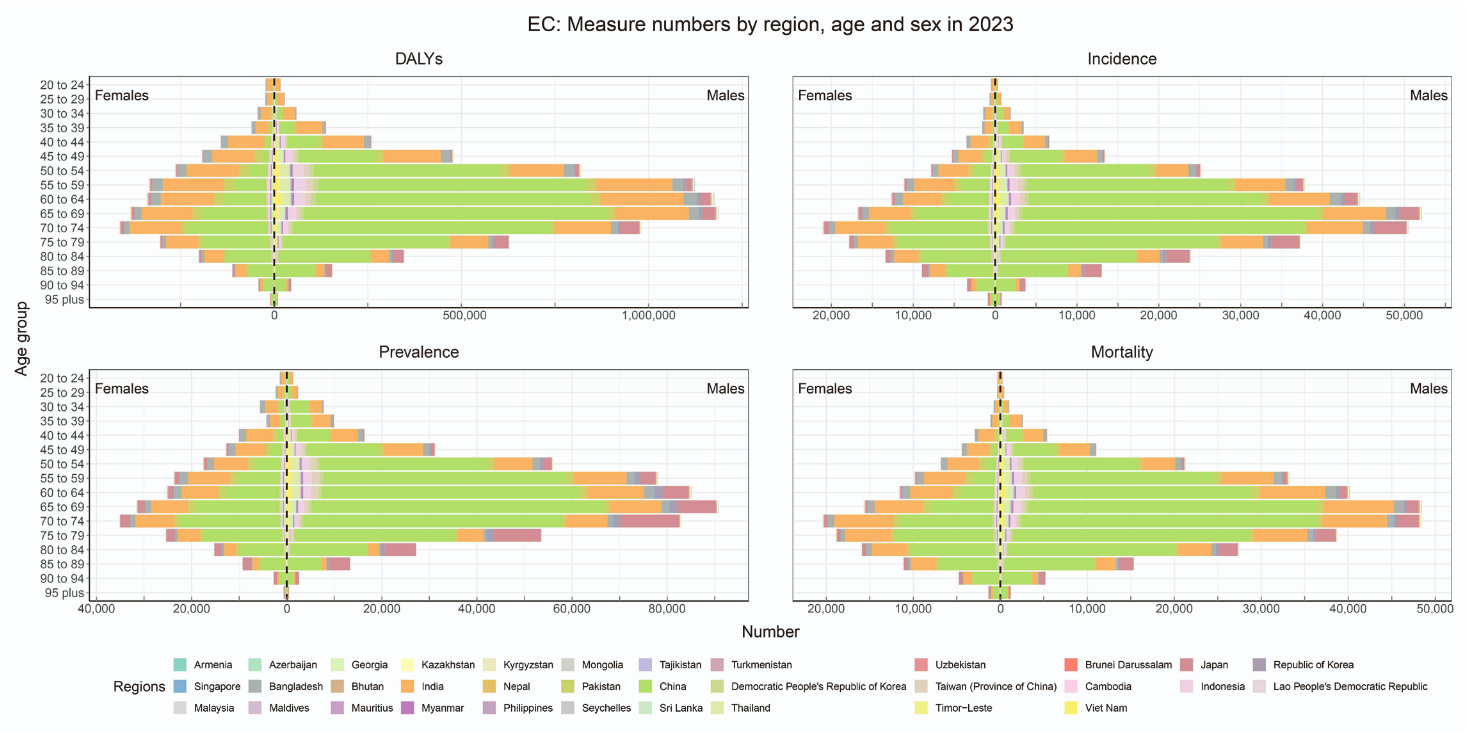

Figure S28: Absolute numbers of Disability-Adjusted Life Years (DALYs), incident cases, prevalent cases, and deaths for esophageal cancer (EC) by regions, sex, and ages, 2023.

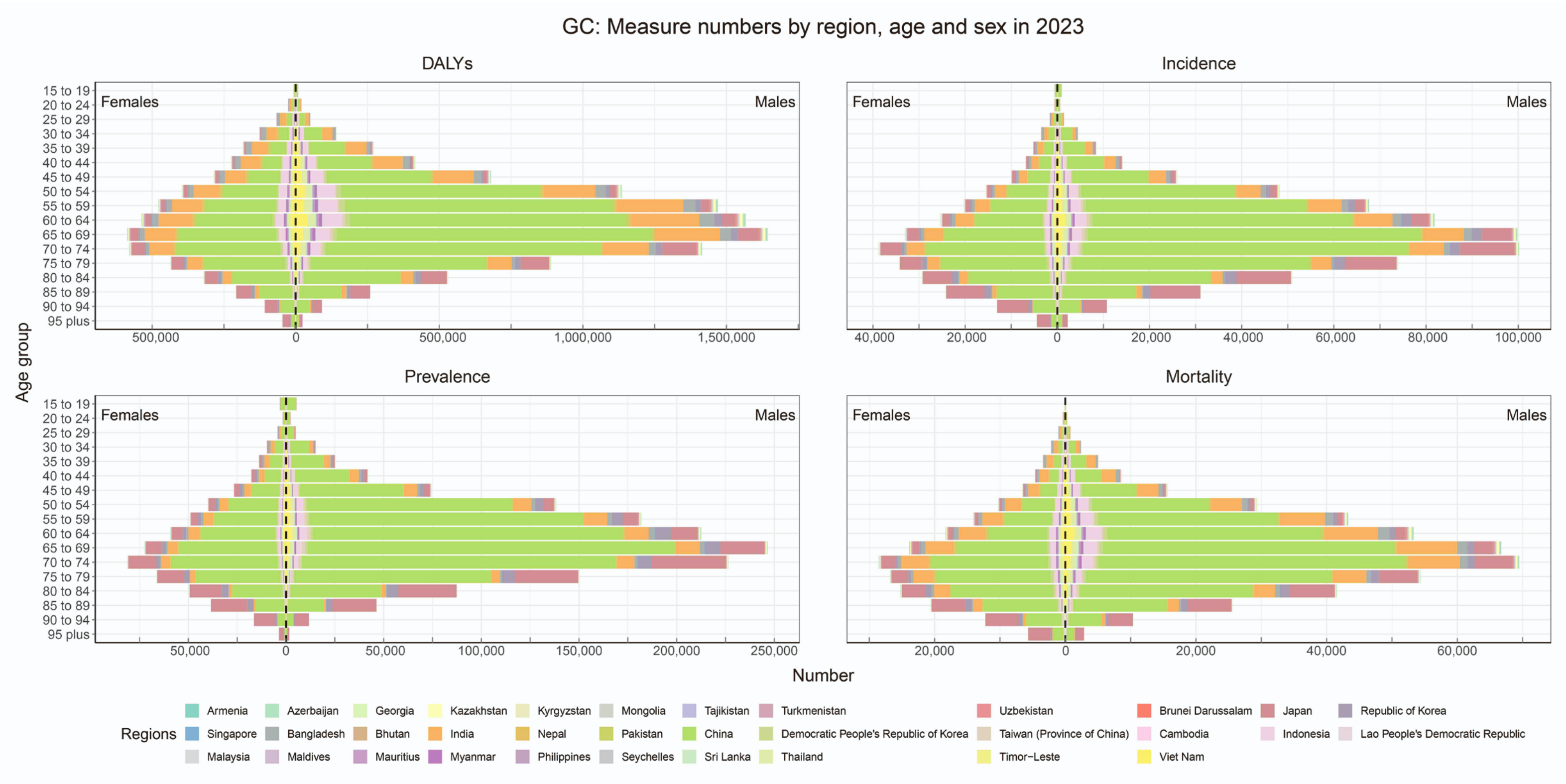

Figure S29: Absolute numbers of Disability-Adjusted Life Years (DALYs), incident cases, prevalent cases, and deaths for gastric cancer (GC) by regions, sex, and ages, 2023.

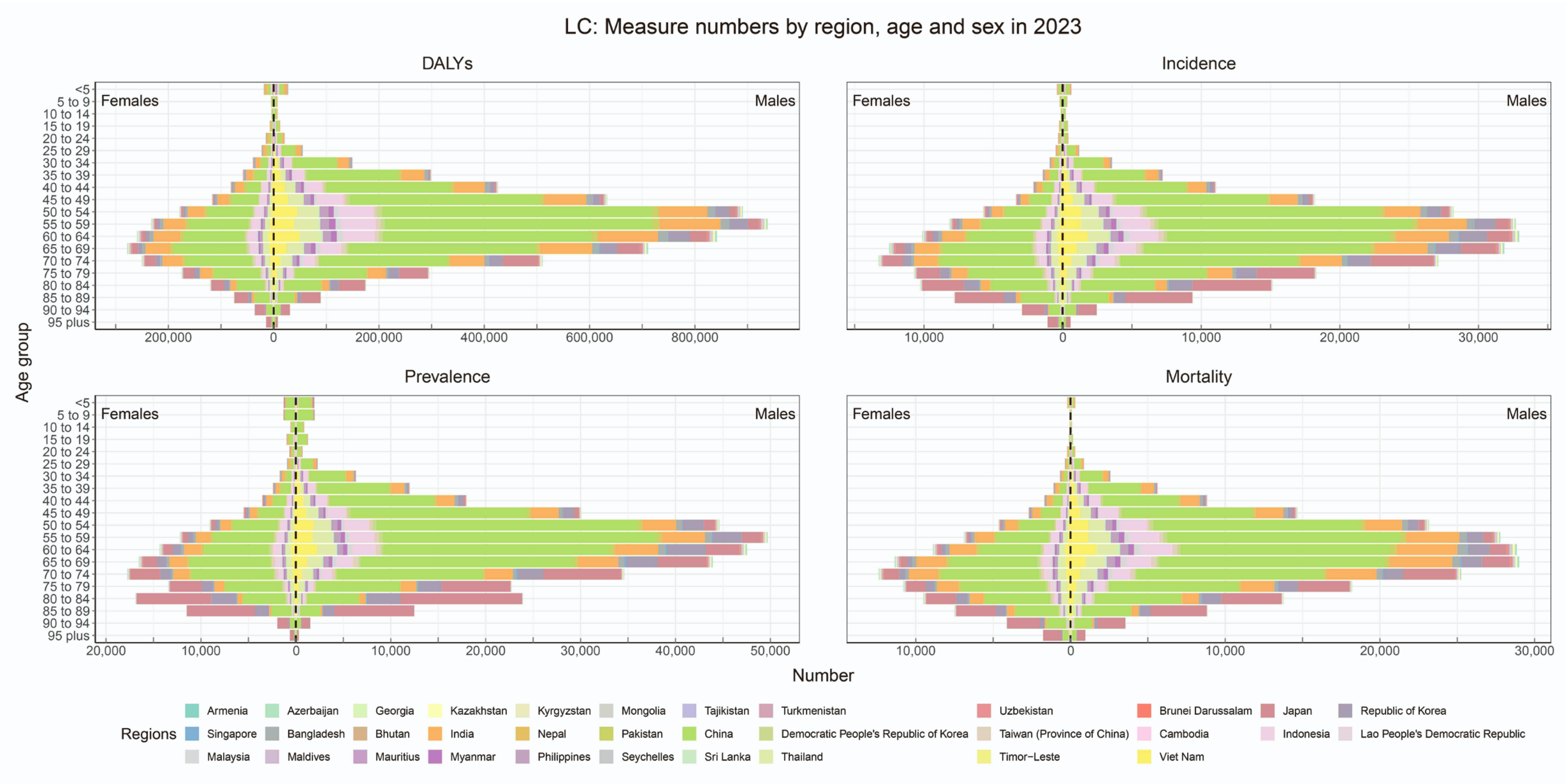

Figure S30: Absolute numbers of Disability-Adjusted Life Years (DALYs), incident cases, prevalent cases, and deaths for liver cancer (LC) by regions, sex, and ages, 2023.

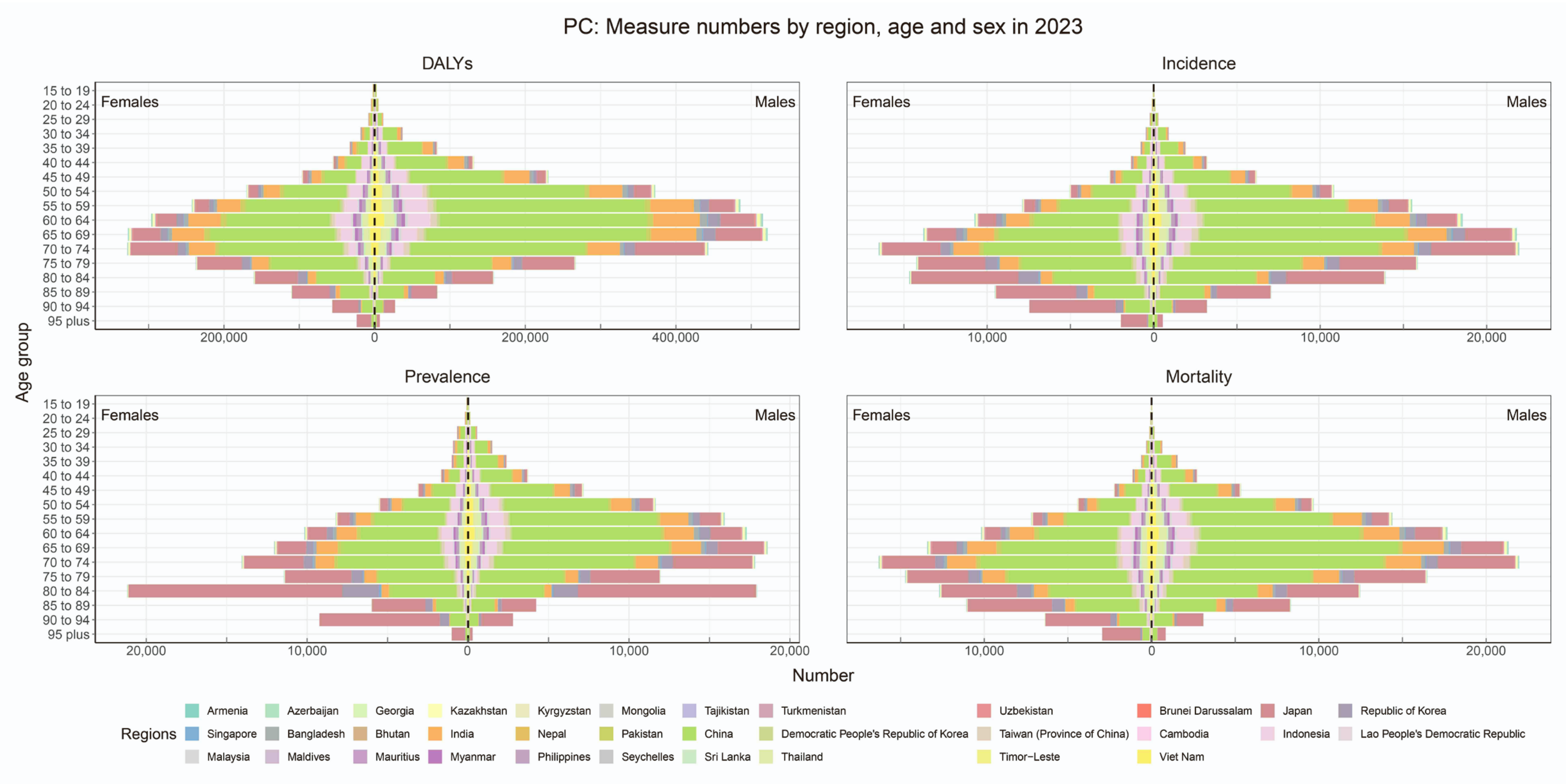

Figure S31: Absolute numbers of Disability-Adjusted Life Years (DALYs), incident cases, prevalent cases, and deaths for pancreatic cancer (PC) by regions, sex, and ages, 2023.

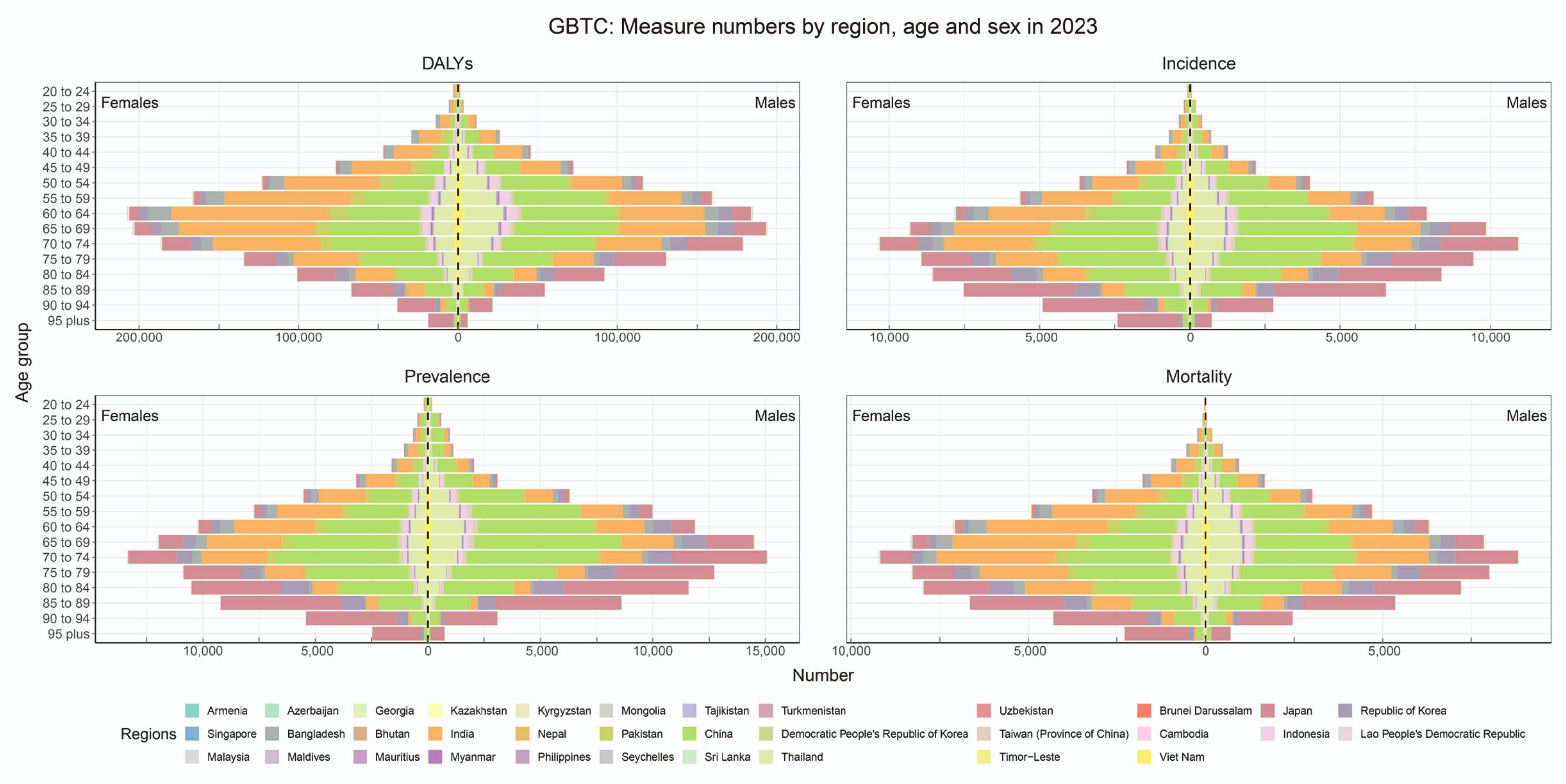

Figure S32: Absolute numbers of Disability-Adjusted Life Years (DALYs), incident cases, prevalent cases, and deaths for gallbladder and biliary tract cancer (GBTC) by regions, sex, and ages, 2023.

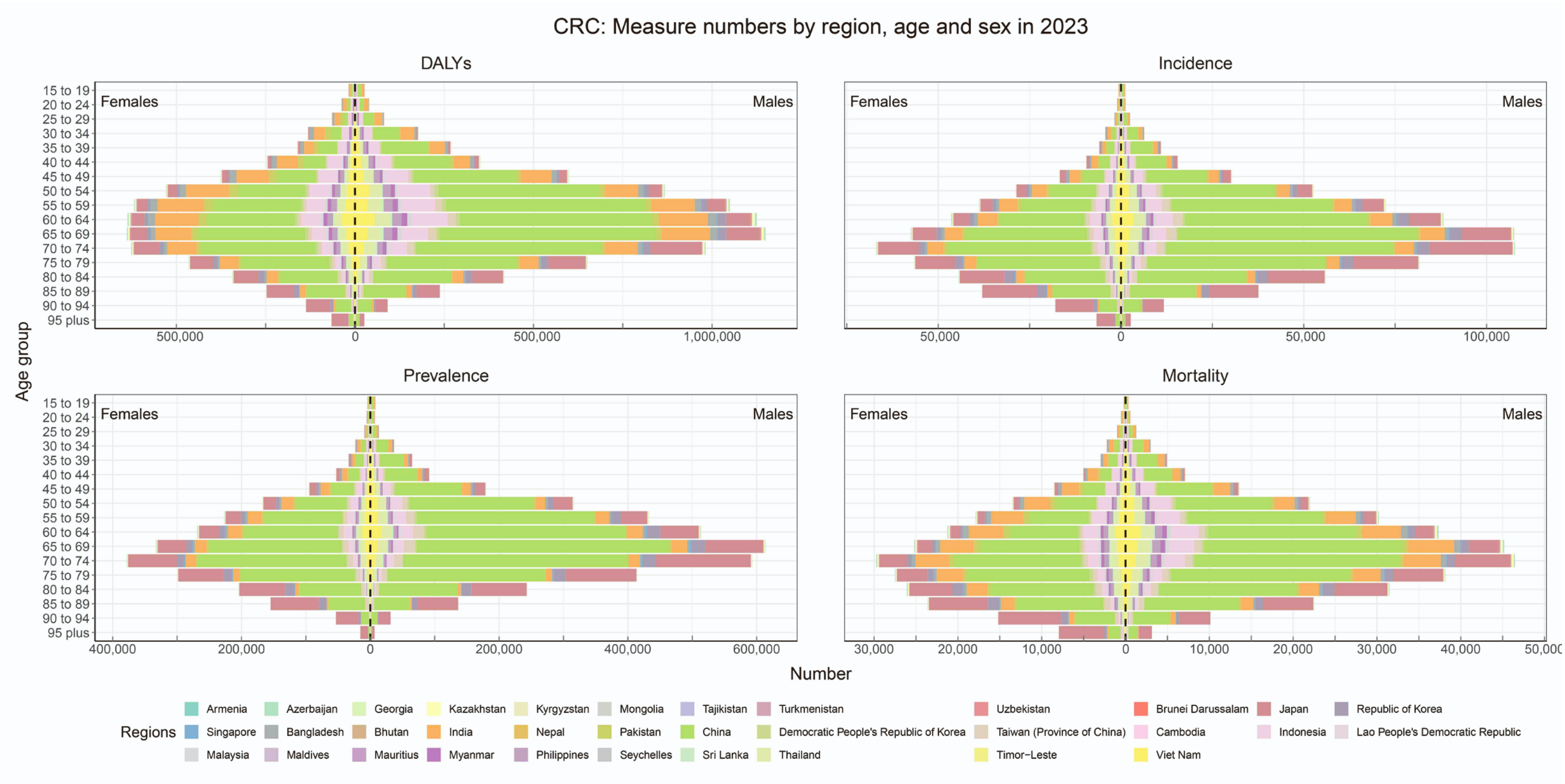

Figure S33: Absolute numbers of Disability-Adjusted Life Years (DALYs), incident cases, prevalent cases, and deaths for colorectal cancer (CRC) by regions, sex, and ages, 2023.

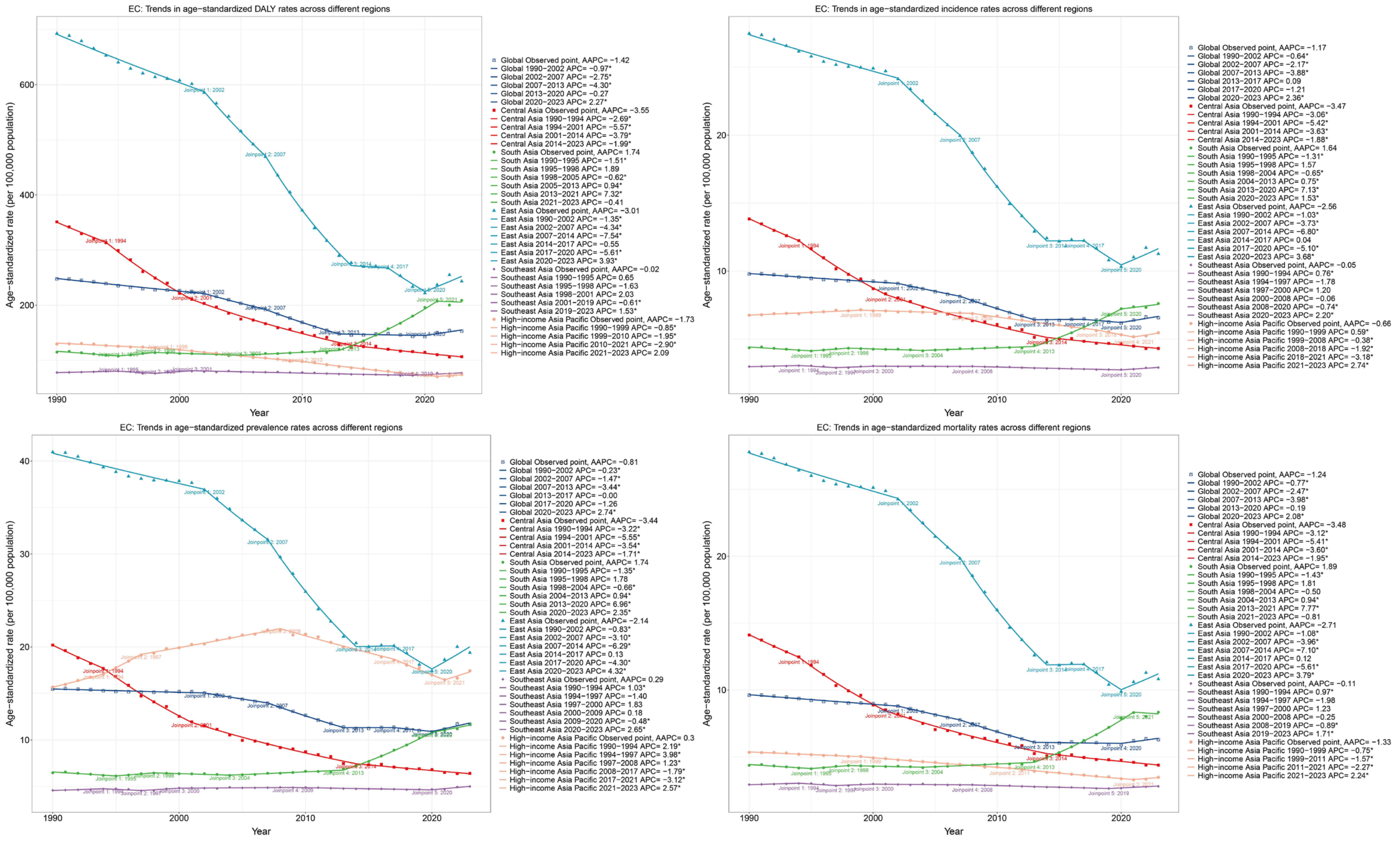

Figure S34: Trends in age-standardized esophageal cancer (EC) rates (DALYs, incidence, prevalence, mortality) by Asian regions, 1990-2023. The results from Joinpoint analysis including annual percent change (APC) and average annual percent change (AAPC). DALYs, disability-adjusted life years.

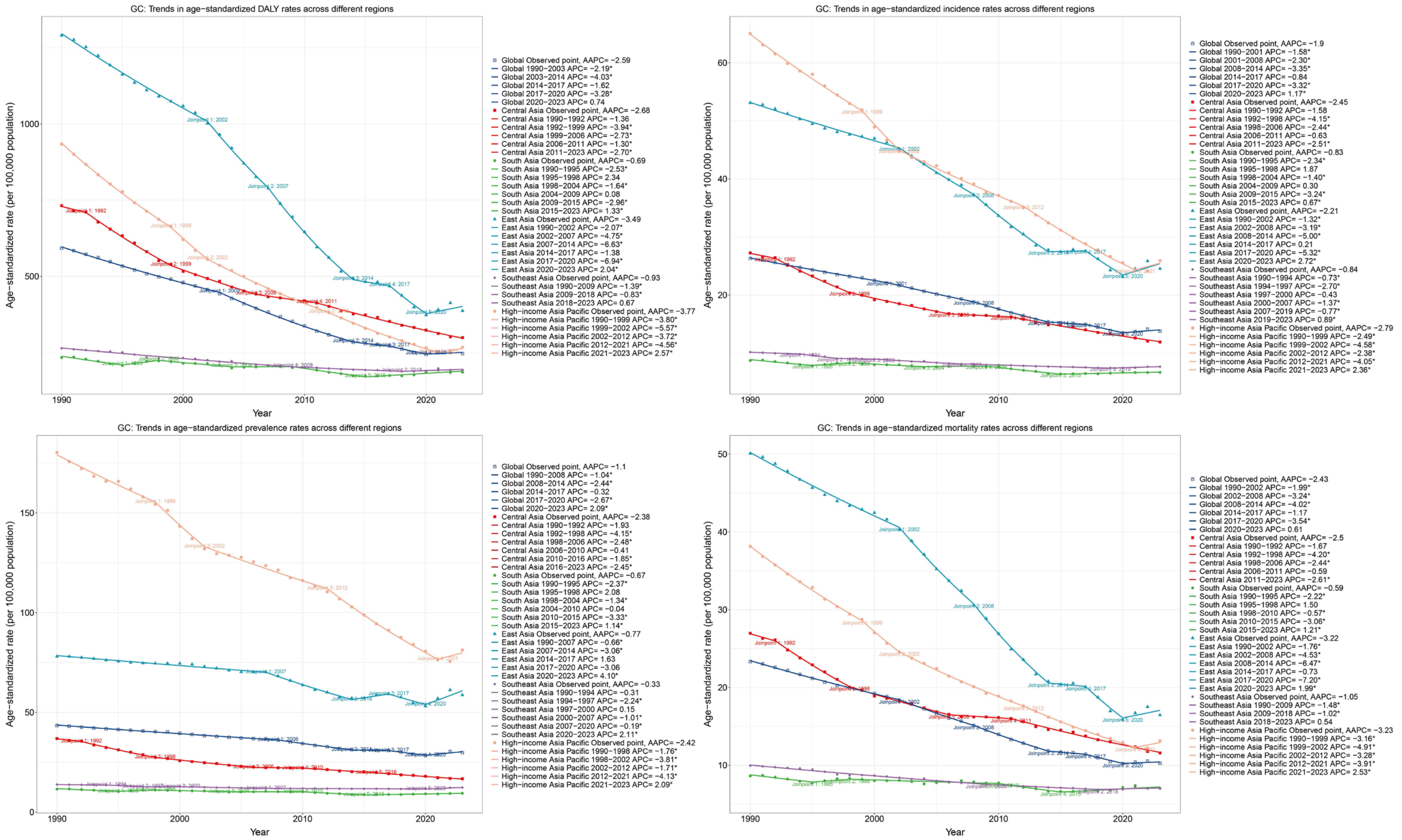

Figure S35: Trends in age-standardized gastric cancer (GC) rates (DALYs, incidence, prevalence, mortality) by Asian regions, 1990-2023. The results from Joinpoint analysis including annual percent change (APC) and average annual percent change (AAPC). DALYs, disability-adjusted life years.

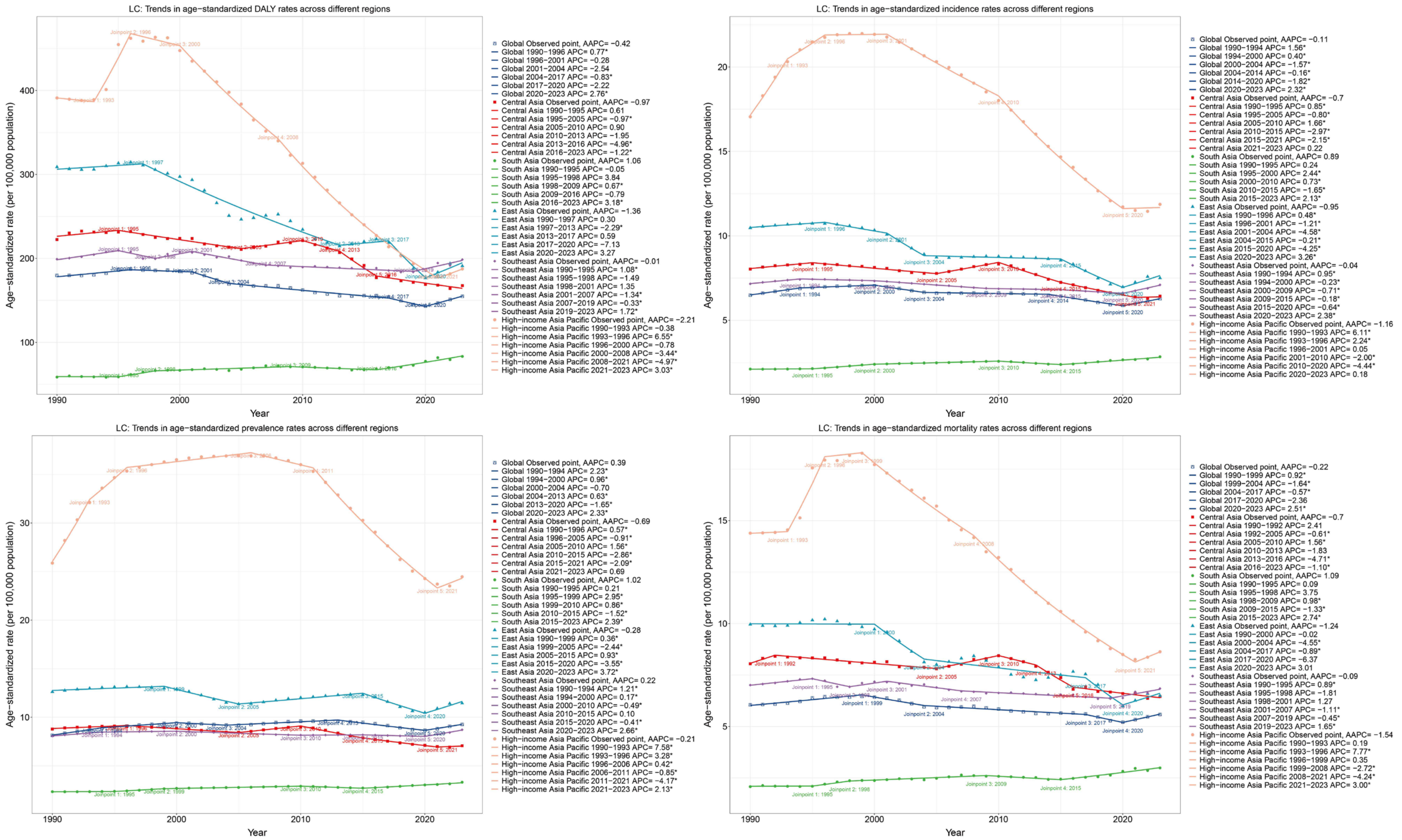

Figure S36: Trends in age-standardized liver cancer (LC) rates (DALYs, incidence, prevalence, mortality) by Asian regions, 1990-2023. The results from Joinpoint analysis including annual percent change (APC) and average annual percent change (AAPC). DALYs, disability-adjusted life years.

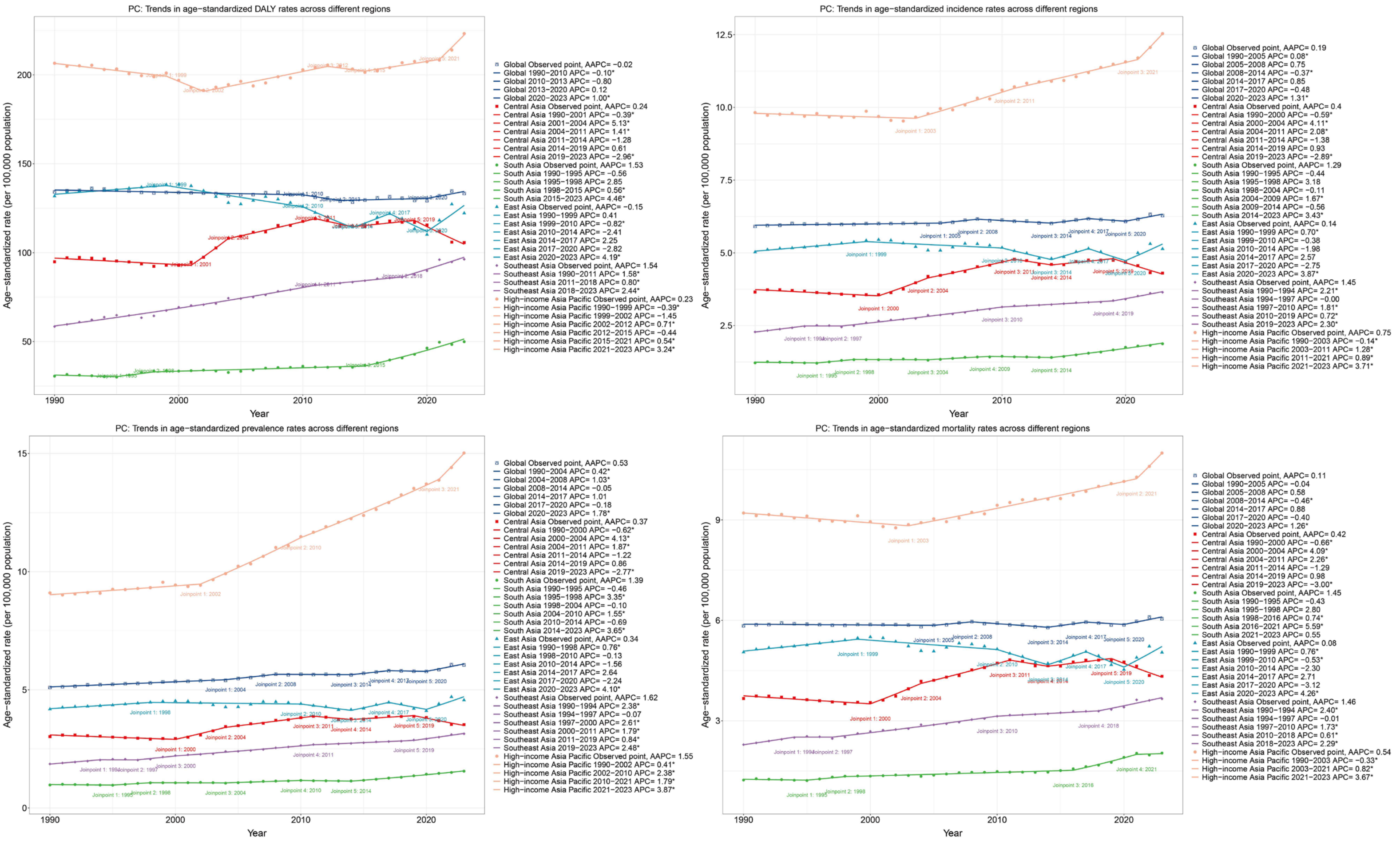

Figure S37: Trends in age-standardized pancreatic cancer (PC) rates (DALYs, incidence, prevalence, mortality) by Asian regions, 1990-2023. The results from Joinpoint analysis including annual percent change (APC) and average annual percent change (AAPC). DALYs, disability-adjusted life years.

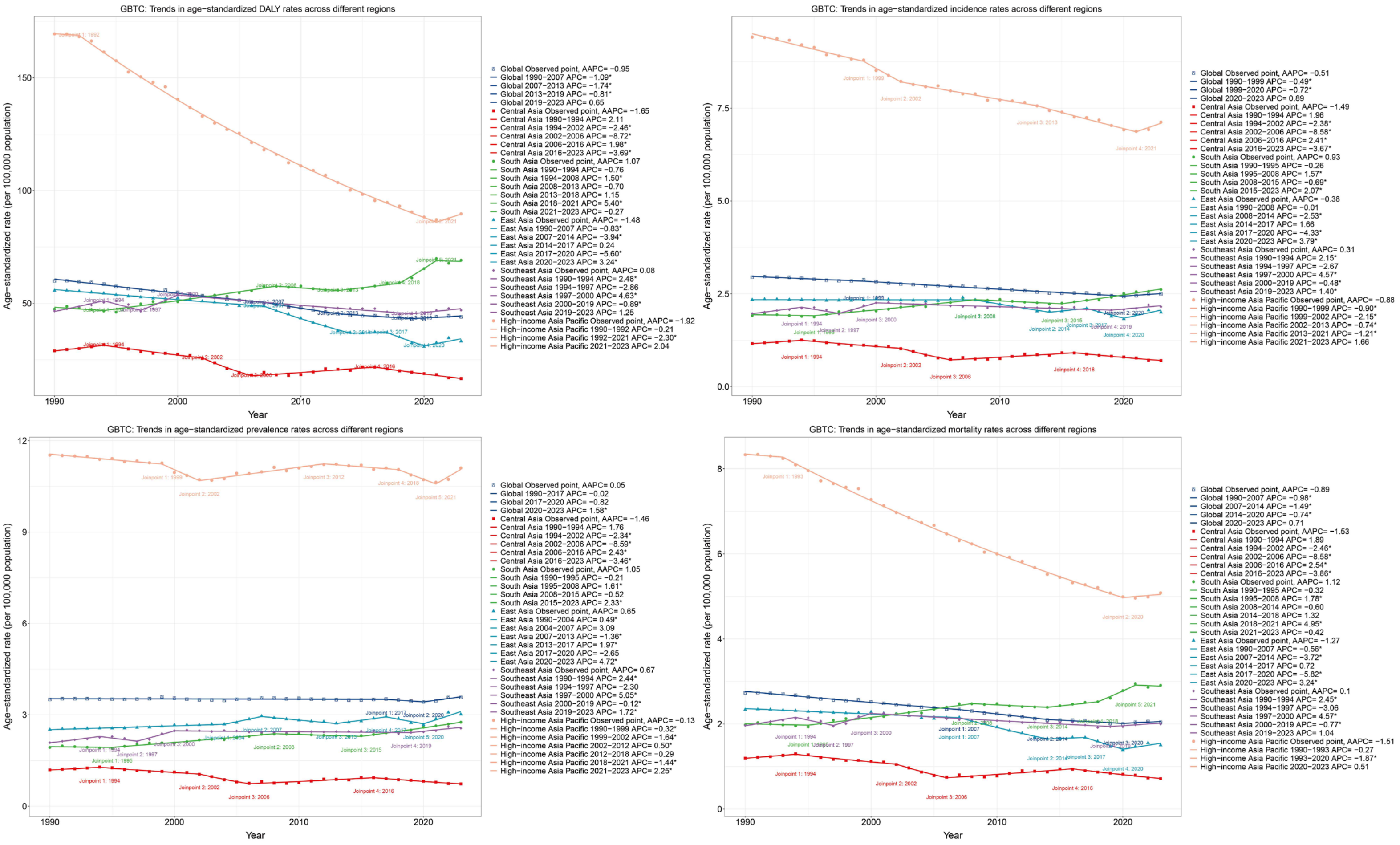

Figure S38: Trends in age-standardized gallbladder and biliary tract cancer (GBTC) rates (DALYs, incidence, prevalence, mortality) by Asian regions, 1990-2023. The results from Joinpoint analysis including annual percent change (APC) and average annual percent change (AAPC). DALYs, disability-adjusted life years.

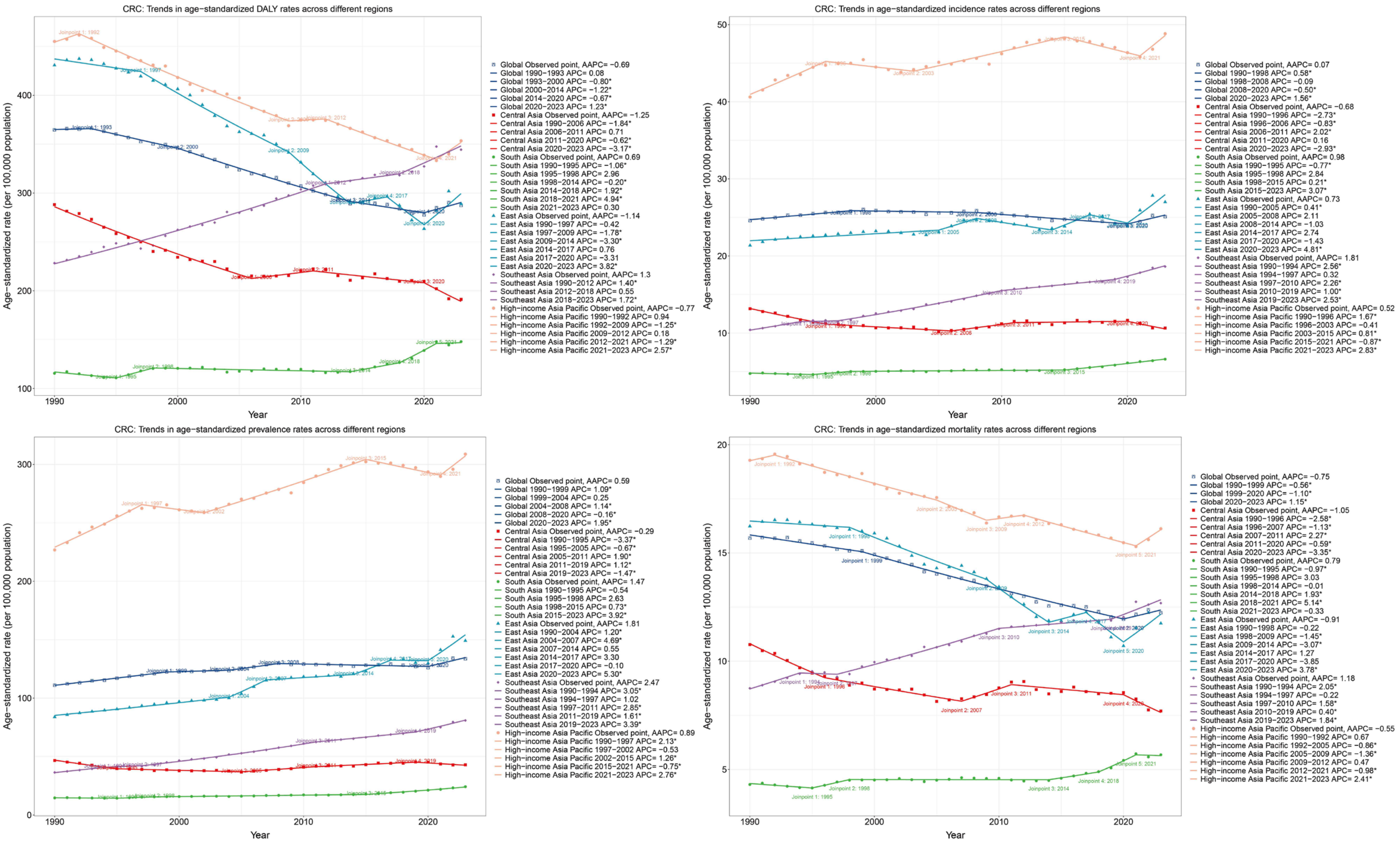

Figure S39. Trends in age-standardized colorectal cancer (CRC) rates (DALYs, incidence, prevalence, mortality) by Asian regions, 1990-2023. The results from Joinpoint analysis including annual percent change (APC) and average annual percent change (AAPC). DALYs, disability-adjusted life years.

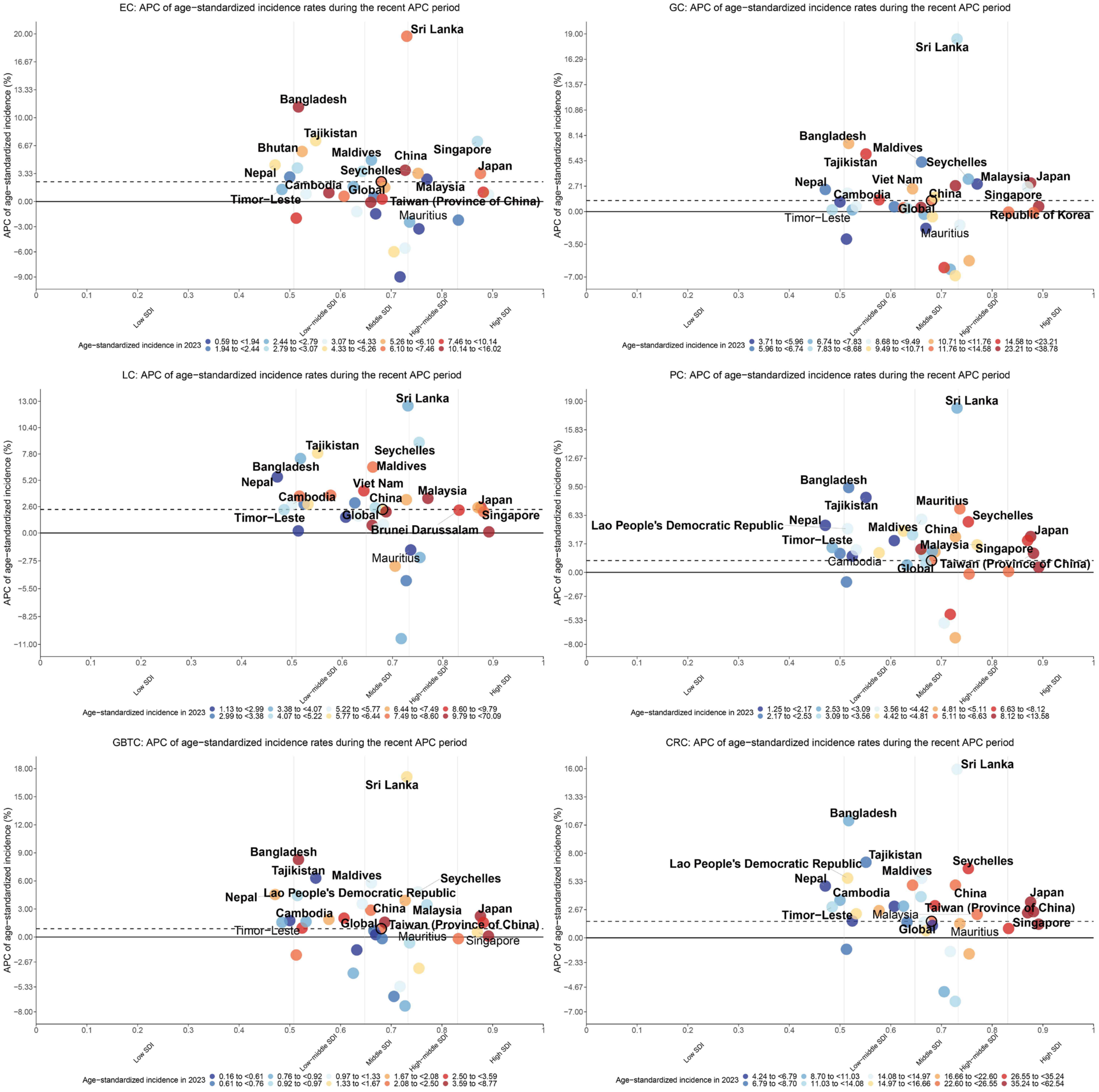

Figure S40: Final segment annual percent change (APC) in age-standardized incidence rates for six major digestive system cancers across Asian countries and territories, highlighting trends during the COVID-19 pandemic and leading countries by Socio-Demographic Index (SDI) quintile. Scatter plots display the APC from the final trend segment (ending 2023) identified by joinpoint regression (y-axis) against the SDI quintile (x-axis) for Asian countries and territories. The color of each point indicates the region's age-standardized incidence rate in 2023 (per 100,000 population). The horizontal dashed line indicates the Global APC value for the corresponding final trend segment. Labels identify the three countries or territories with the highest APC within each SDI quintile. Labels are bolded if the final trend segment began in 2019, 2020, or 2021, indicating a trend change during the COVID-19 pandemic period. EC, esophageal cancer; GC, gastric cancer; LC, liver cancer; PC, pancreatic cancer; GBTC, gallbladder and biliary tract cancer; CRC, colorectal cancer.

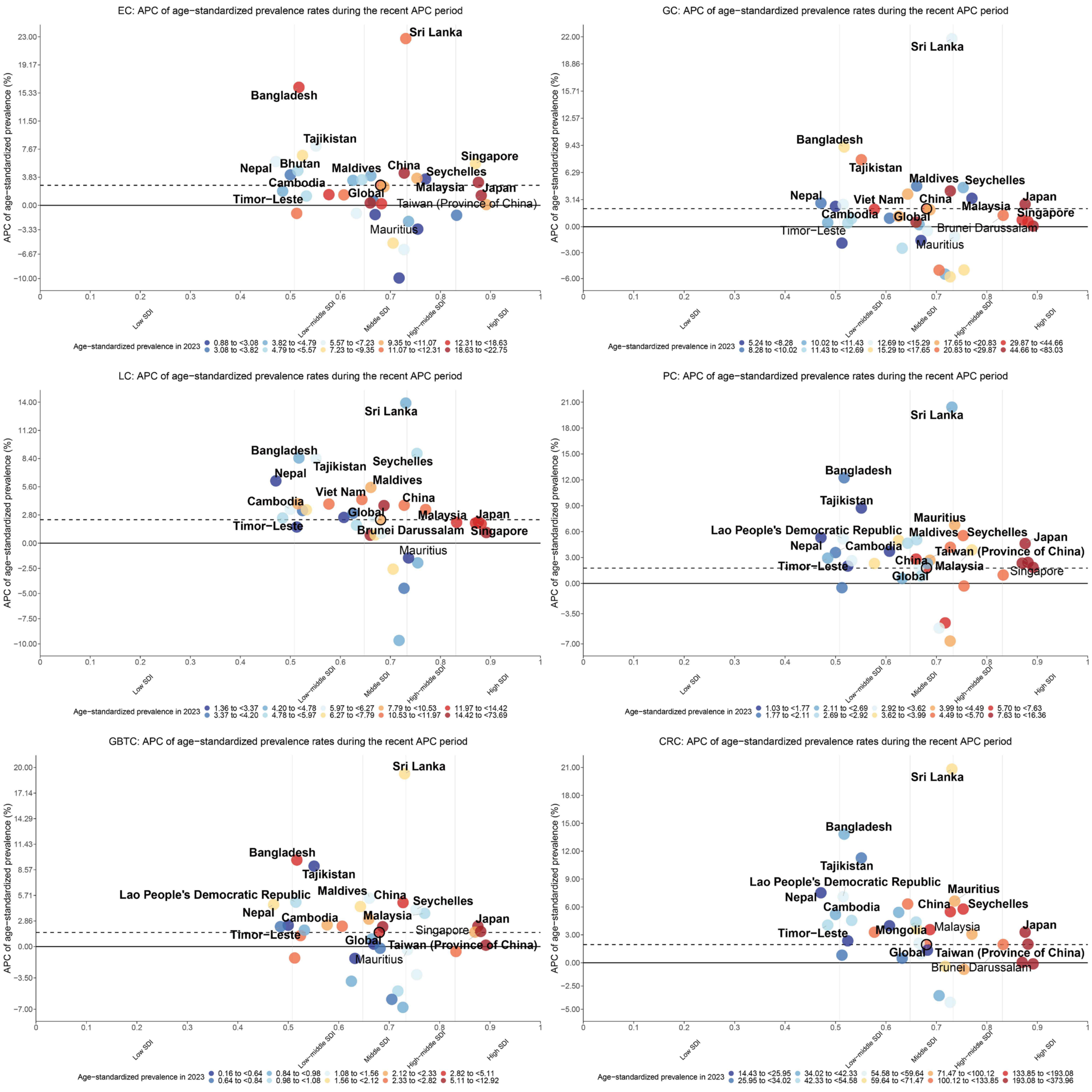

Figure S41: Final segment annual percent change (APC) in age-standardized prevalence rates for six major digestive system cancers across Asian countries and territories, highlighting trends during the COVID-19 pandemic and leading countries by Socio-Demographic Index (SDI) quintile. Scatter plots display the APC from the final trend segment (ending 2023) identified by joinpoint regression (y-axis) against the SDI quintile (x-axis) for Asian countries and territories. The color of each point indicates the region's age-standardized prevalence rate in 2023 (per 100,000 population). The horizontal dashed line indicates the Global APC value for the corresponding final trend segment. Labels identify the three countries or territories with the highest APC within each SDI quintile. Labels are bolded if the final trend segment began in 2019, 2020, or 2021, indicating a trend change during the COVID-19 pandemic period. EC, esophageal cancer; GC, gastric cancer; LC, liver cancer; PC, pancreatic cancer; GBTC, gallbladder and biliary tract cancer; CRC, colorectal cancer.

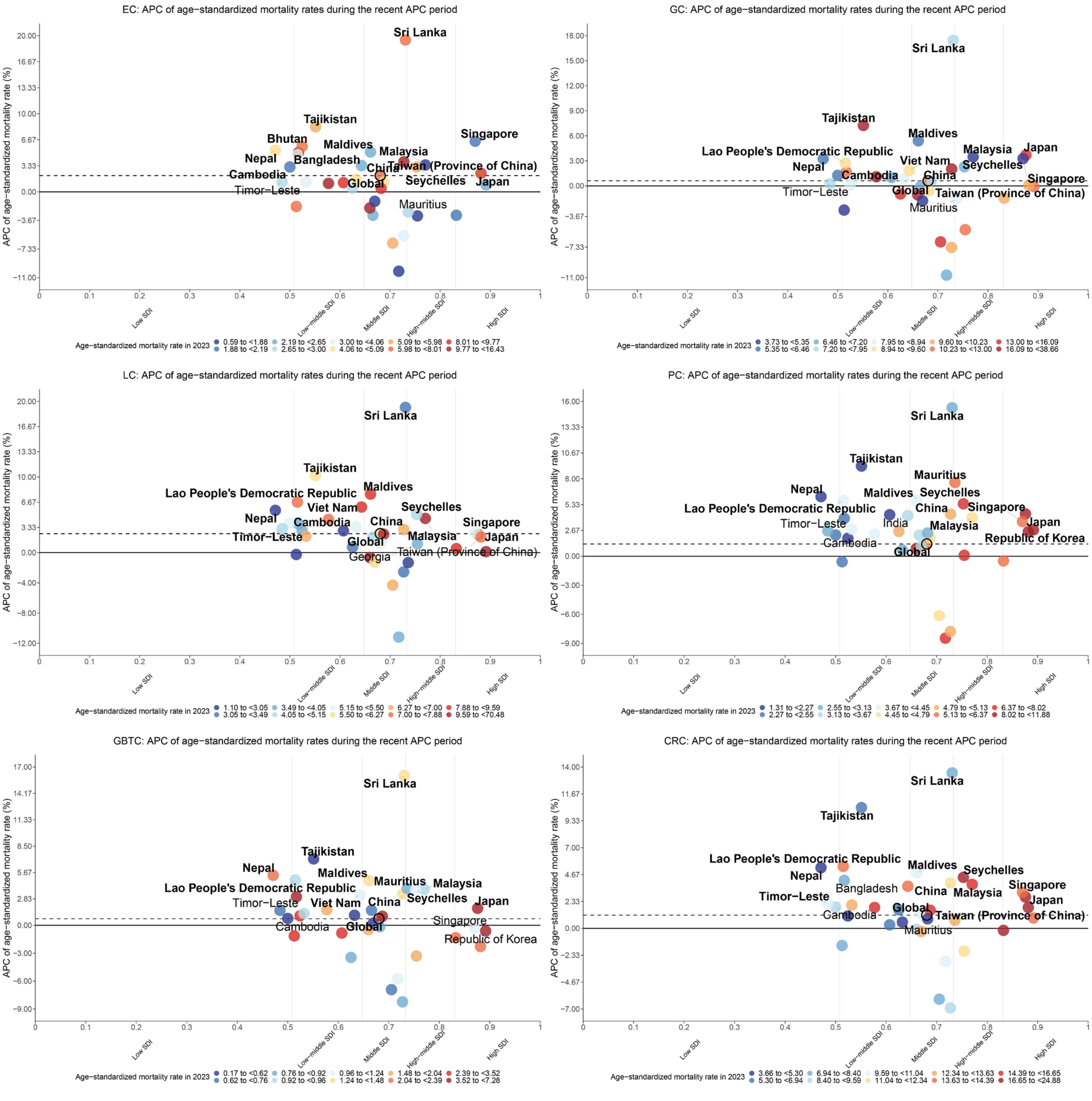

Figure S42: Final segment annual percent change (APC) in age-standardized mortality rates for six major digestive system cancers across Asian countries and territories, highlighting trends during the COVID-19 pandemic and leading countries by Socio-Demographic Index (SDI) quintile. Scatter plots display the APC from the final trend segment (ending 2023) identified by joinpoint regression (y-axis) against the SDI quintile (x-axis) for Asian countries and territories. The color of each point indicates the region's age-standardized mortality rate in 2023 (per 100,000 population). The horizontal dashed line indicates the Global APC value for the corresponding final trend segment. Labels identify the three countries or territories with the highest APC within each SDI quintile. Labels are bolded if the final trend segment began in 2019, 2020, or 2021, indicating a trend change during the COVID-19 pandemic period. EC, esophageal cancer; GC, gastric cancer; LC, liver cancer; PC, pancreatic cancer; GBTC, gallbladder and biliary tract cancer; CRC, colorectal cancer.

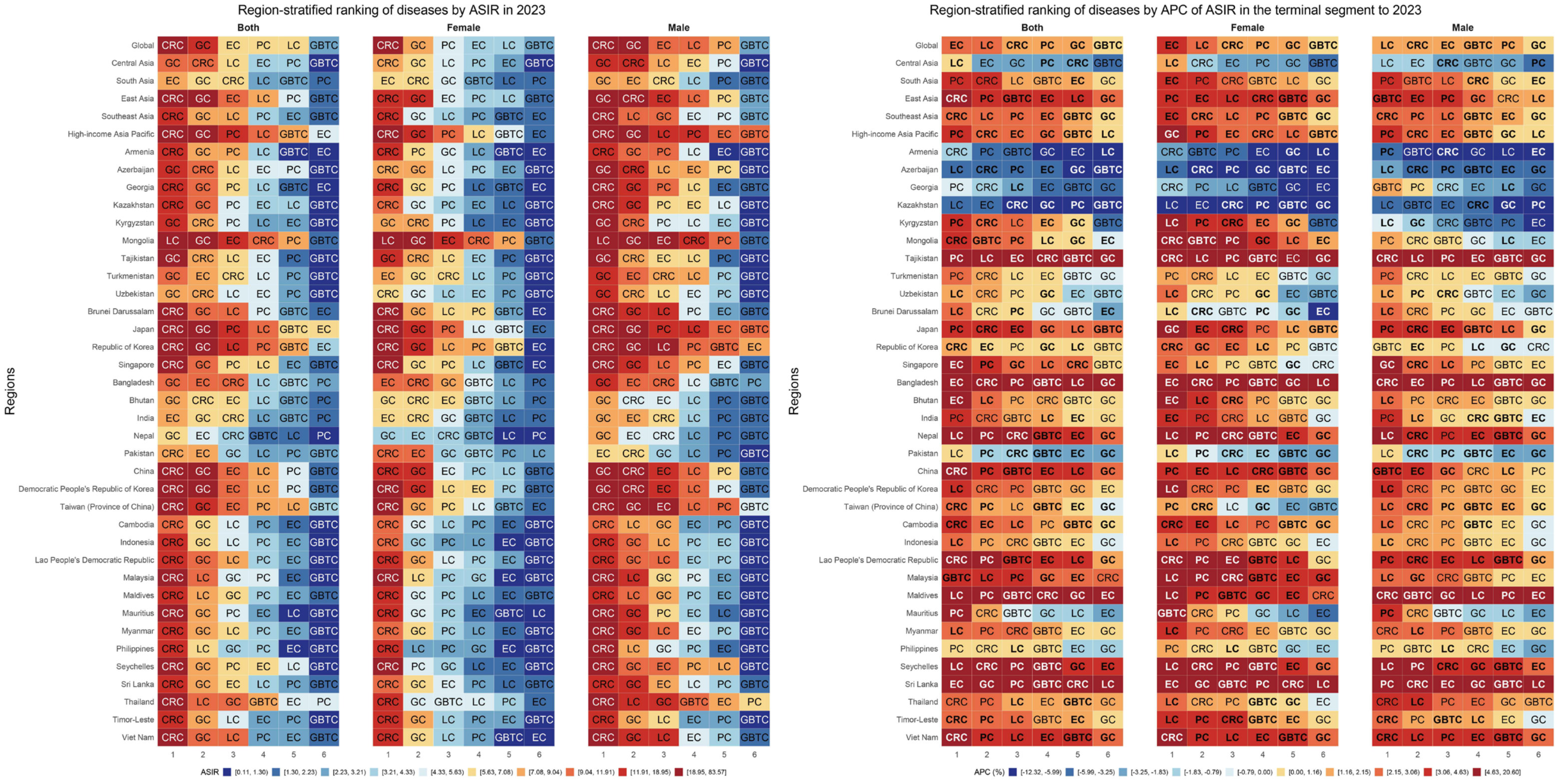

Figure S43: Comparative ranking of six major digestive system cancers based on 2023 incidence rates and recent trends (final segment APC), across geographic regions by sex. Cell color corresponds to the magnitude of the ranked metric, as indicated by the color scale for each panel. Cancer abbreviations within cells are bolded if the final trend segment began in 2019, 2020, or 2021, indicating a trend change during the COVID-19 pandemic period. ASIR, age-standardized incidence rate; APC, annual percent change; EC, esophageal cancer; GC, gastric cancer; LC, liver cancer; PC, pancreatic cancer; GBTC, gallbladder and biliary tract cancer; CRC, colorectal cancer.

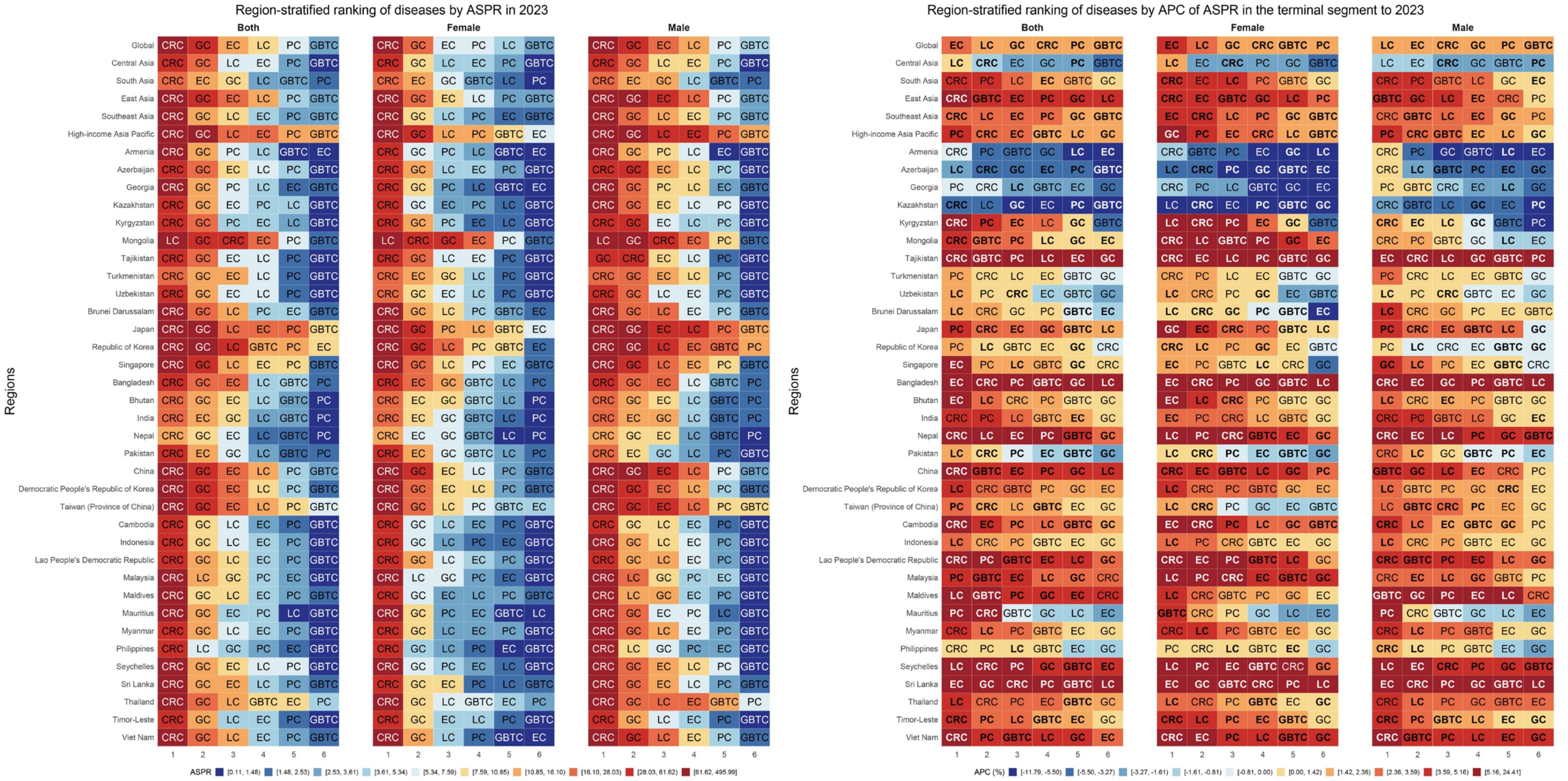

Figure S44: Comparative ranking of six major digestive system cancers based on 2023 prevalence rates and recent trends (final segment APC), across geographic regions by sex. Cell color corresponds to the magnitude of the ranked metric, as indicated by the color scale for each panel. Cancer abbreviations within cells are bolded if the final trend segment began in 2019, 2020, or 2021, indicating a trend change during the COVID-19 pandemic period. ASPR, age-standardized prevalence rate; APC, annual percent change; EC, esophageal cancer; GC, gastric cancer; LC, liver cancer; PC, pancreatic cancer; GBTC, gallbladder and biliary tract cancer; CRC, colorectal cancer.

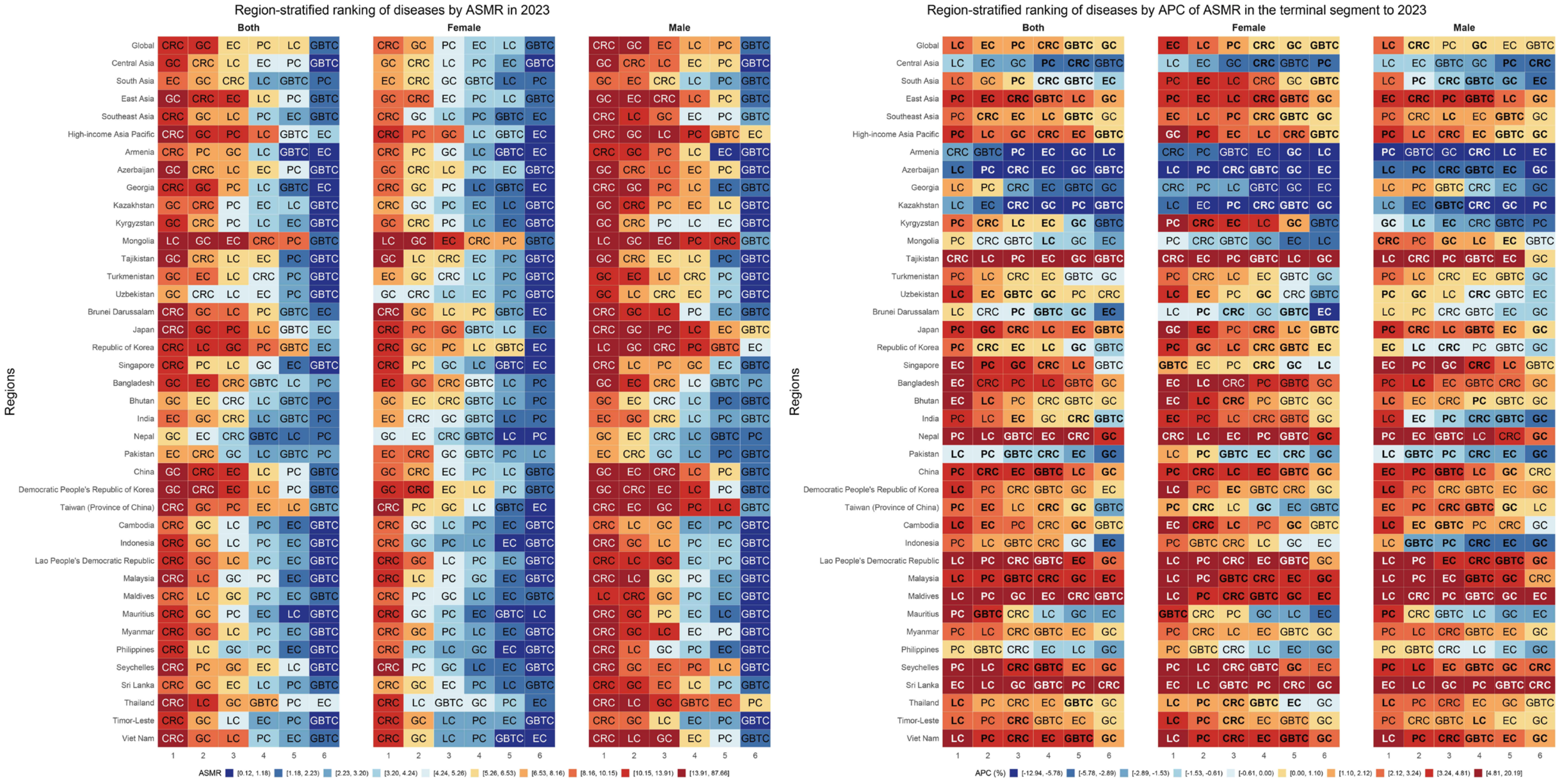

Figure S45: Comparative ranking of six major digestive system cancers based on 2023 mortality rates and recent trends (final segment APC), across geographic regions by sex. Cell color corresponds to the magnitude of the ranked metric, as indicated by the color scale for each panel. Cancer abbreviations within cells are bolded if the final trend segment began in 2019, 2020, or 2021, indicating a trend change during the COVID-19 pandemic period. ASMR, age-standardized mortality rate; APC, annual percent change; EC, esophageal cancer; GC, gastric cancer; LC, liver cancer; PC, pancreatic cancer; GBTC, gallbladder and biliary tract cancer; CRC, colorectal cancer.

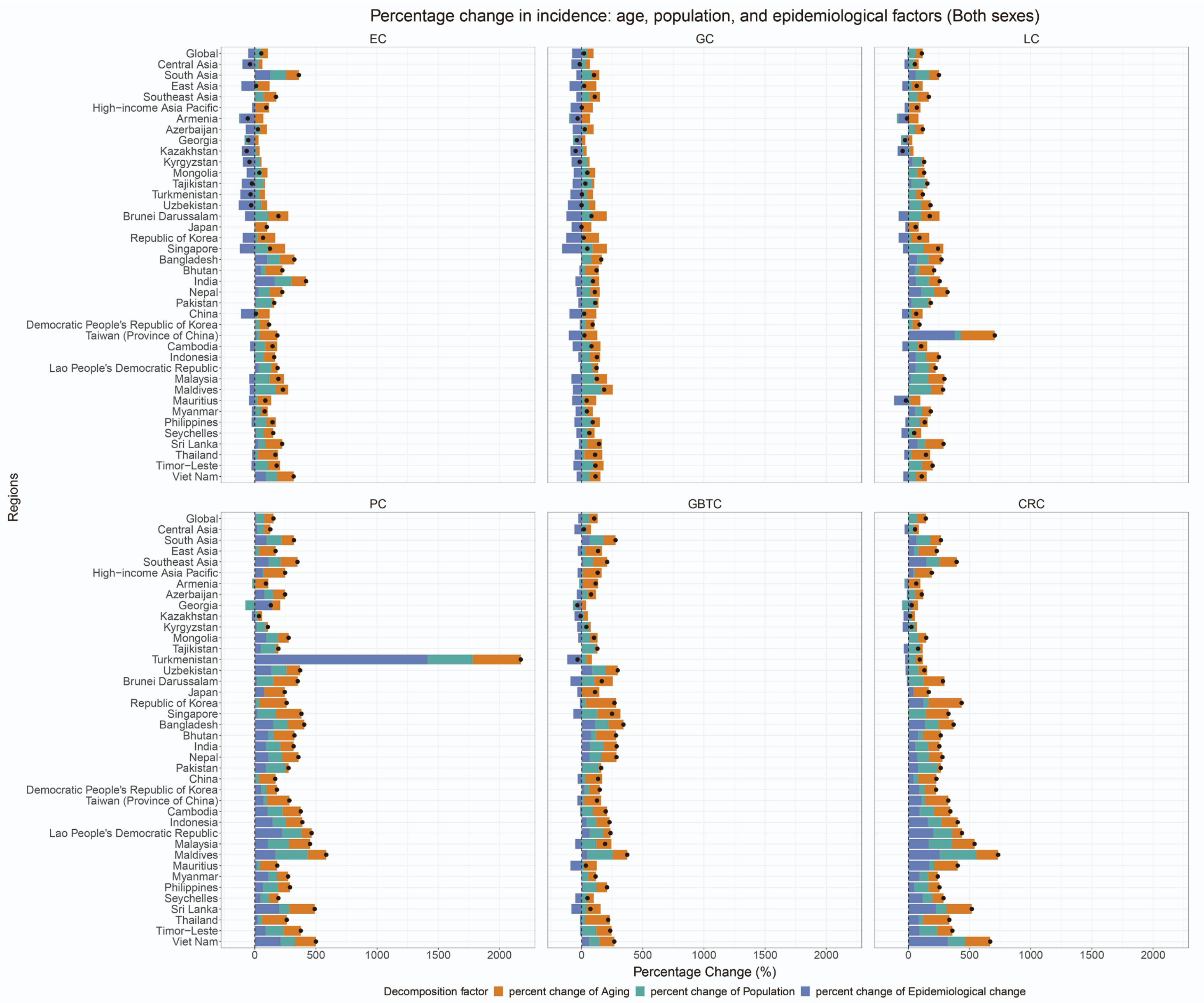

Figure S46: Decomposition analysis of the percentage change in absolute incidence numbers between 1990 and 2023 for six major digestive system cancers across Asian regions. The total percentage change (indicated by points) is attributed to changes in population aging, population growth, and epidemiological factors (changes in age-specific incidence rates), represented by the colored bars. EC, esophageal cancer; GC, gastric cancer; LC, liver cancer; PC, pancreatic cancer; GBTC, gallbladder and biliary tract cancer; CRC, colorectal cancer.

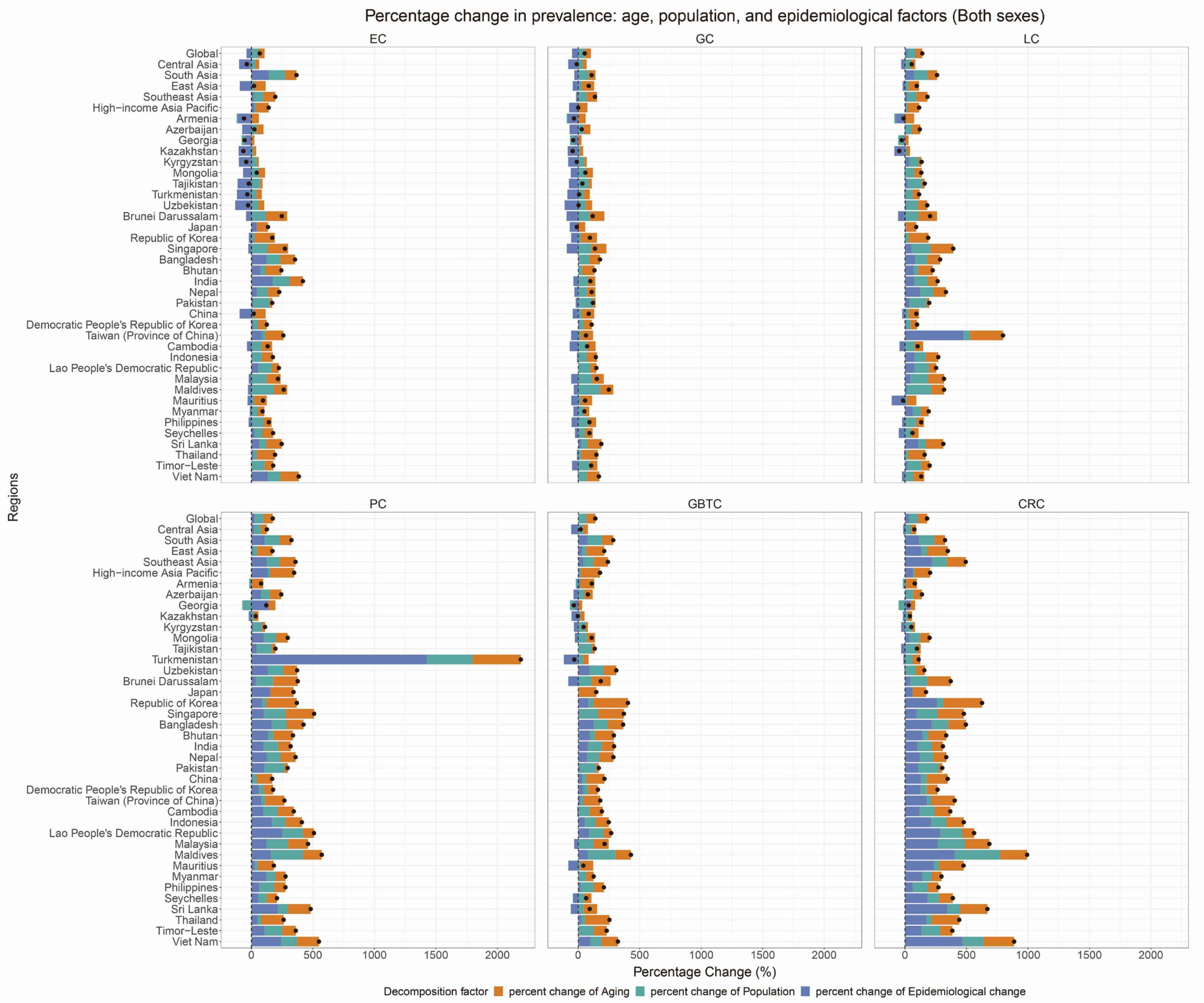

Figure S47: Decomposition analysis of the percentage change in absolute prevalence numbers between 1990 and 2023 for six major digestive system cancers across Asian regions. The total percentage change (indicated by points) is attributed to changes in population aging, population growth, and epidemiological factors (changes in age-specific prevalence rates), represented by the colored bars. EC, esophageal cancer; GC, gastric cancer; LC, liver cancer; PC, pancreatic cancer; GBTC, gallbladder and biliary tract cancer; CRC, colorectal cancer.

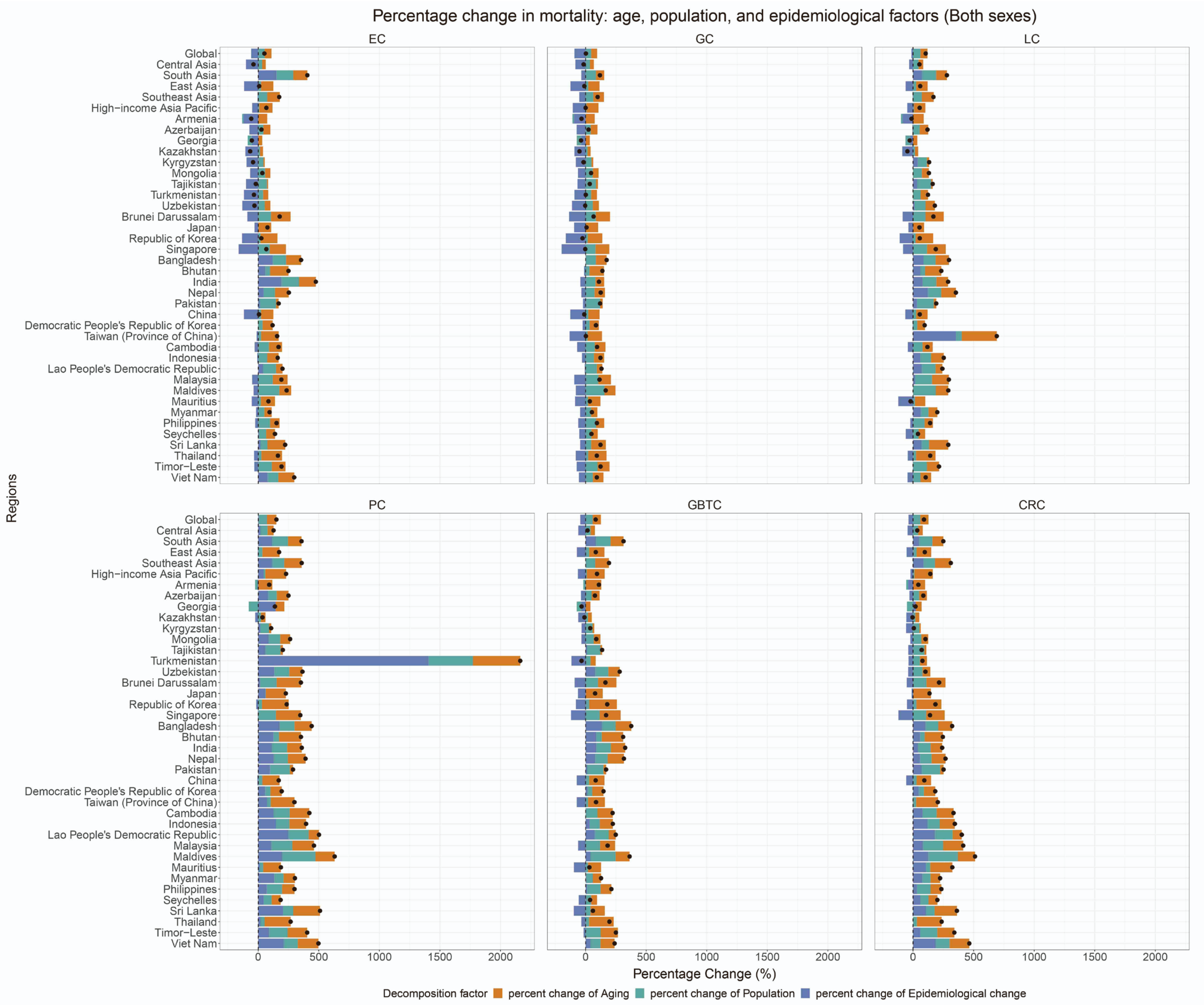

Figure S48: Decomposition analysis of the percentage change in absolute mortality numbers between 1990 and 2023 for six major digestive system cancers across Asian regions. The total percentage change (indicated by points) is attributed to changes in population aging, population growth, and epidemiological factors (changes in age-specific mortality rates), represented by the colored bars. EC, esophageal cancer; GC, gastric cancer; LC, liver cancer; PC, pancreatic cancer; GBTC, gallbladder and biliary tract cancer; CRC, colorectal cancer.

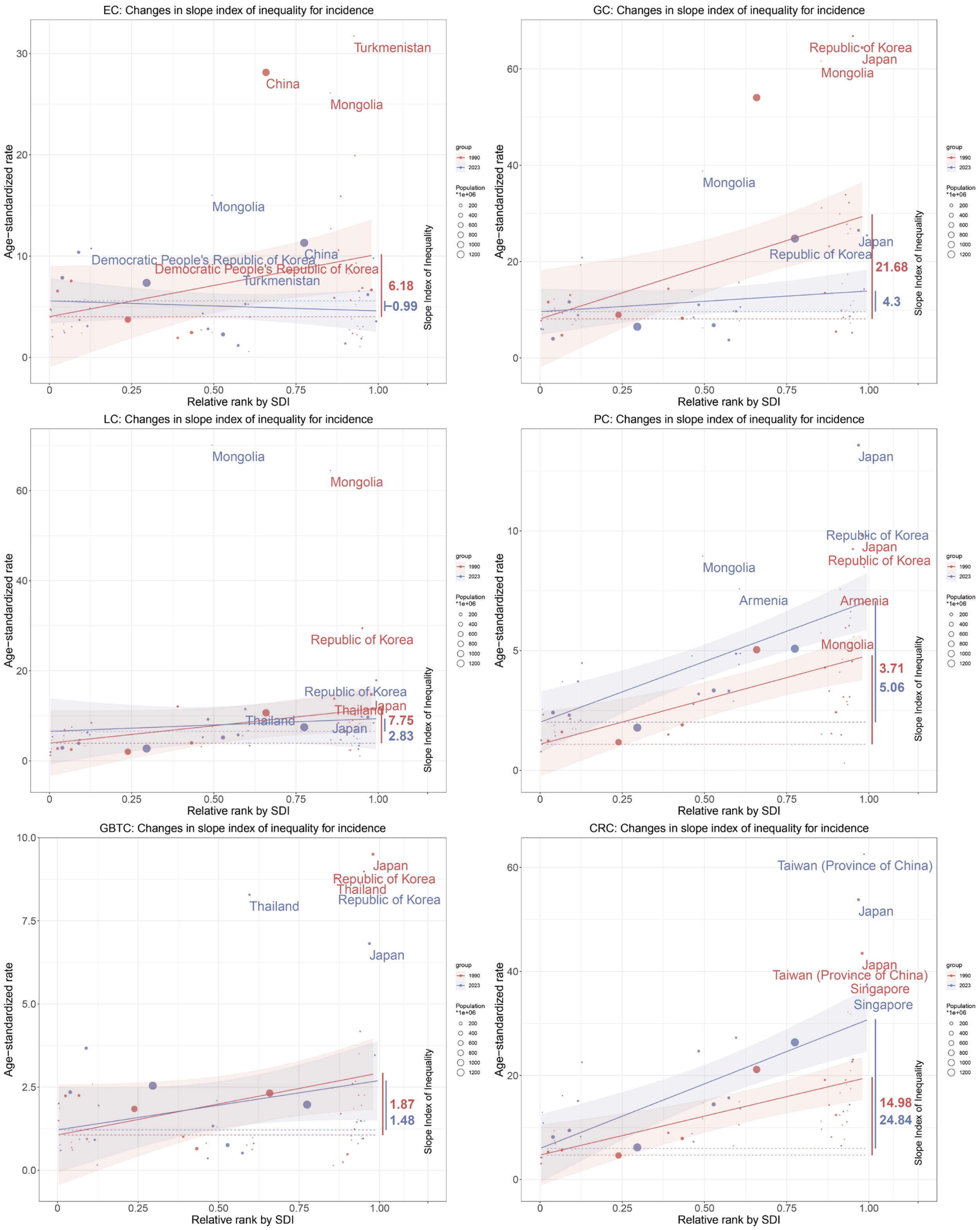

Figure S49: Changes in Absolute Inequality (Slope Index of Inequality) for age-standardized incidence rates of six major digestive system cancers across the SDI spectrum, 1990-2023. Scatter plots display age-standardized rates (per 100,000 population) in 1990 (red points/line) and 2023 (blue points/line) against the relative rank of countries/territories by SDI. Labels indicate the three countries or territories with the highest age-standardized rates in 1990 and 2023, respectively. EC, esophageal cancer; GC, gastric cancer; LC, liver cancer; PC, pancreatic cancer; GBTC, gallbladder and biliary tract cancer; CRC, colorectal cancer.

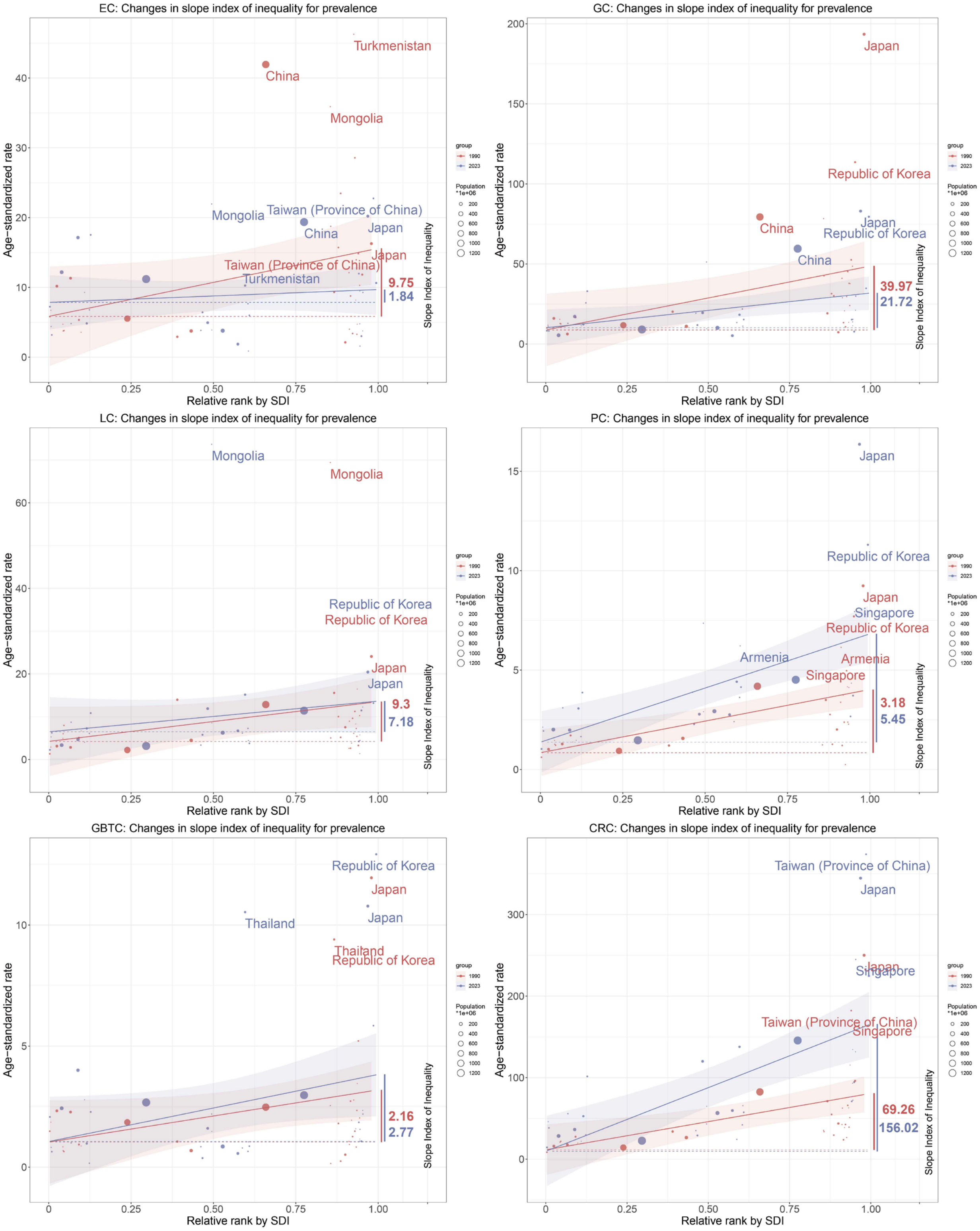

Figure S50: Changes in Absolute Inequality (Slope Index of Inequality) for age-standardized prevalence rates of six major digestive system cancers across the SDI spectrum, 1990-2023. Scatter plots display age-standardized rates (per 100,000 population) in 1990 (red points/line) and 2023 (blue points/line) against the relative rank of countries/territories by SDI. Labels indicate the three countries or territories with the highest age-standardized rates in 1990 and 2023, respectively. EC, esophageal cancer; GC, gastric cancer; LC, liver cancer; PC, pancreatic cancer; GBTC, gallbladder and biliary tract cancer; CRC, colorectal cancer.

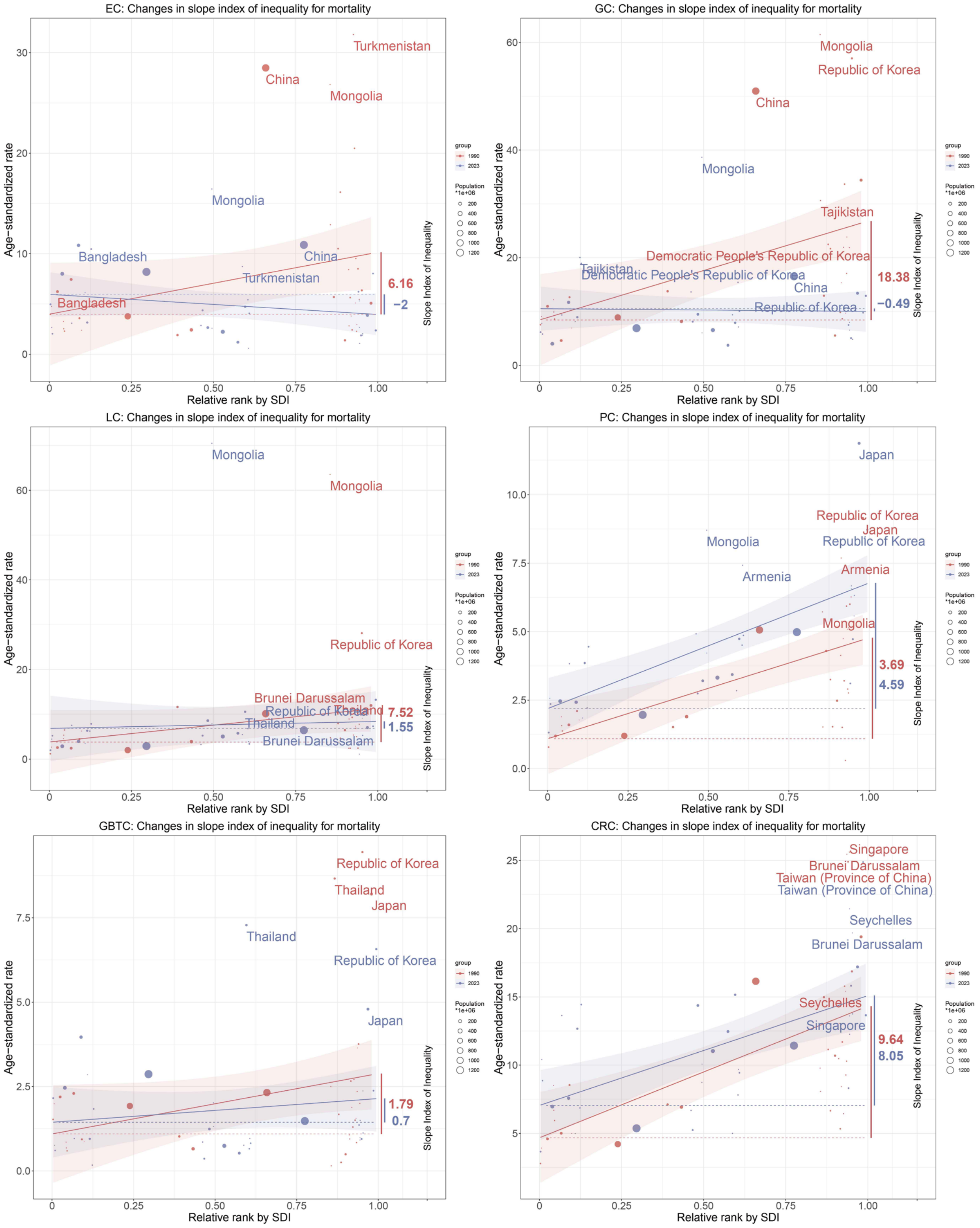

Figure S51: Changes in Absolute Inequality (Slope Index of Inequality) for age-standardized mortality rates of six major digestive system cancers across the SDI spectrum, 1990-2023. Scatter plots display age-standardized rates (per 100,000 population) in 1990 (red points/line) and 2023 (blue points/line) against the relative rank of countries/territories by SDI. Labels indicate the three countries or territories with the highest age-standardized rates in 1990 and 2023, respectively. EC, esophageal cancer; GC, gastric cancer; LC, liver cancer; PC, pancreatic cancer; GBTC, gallbladder and biliary tract cancer; CRC, colorectal cancer.

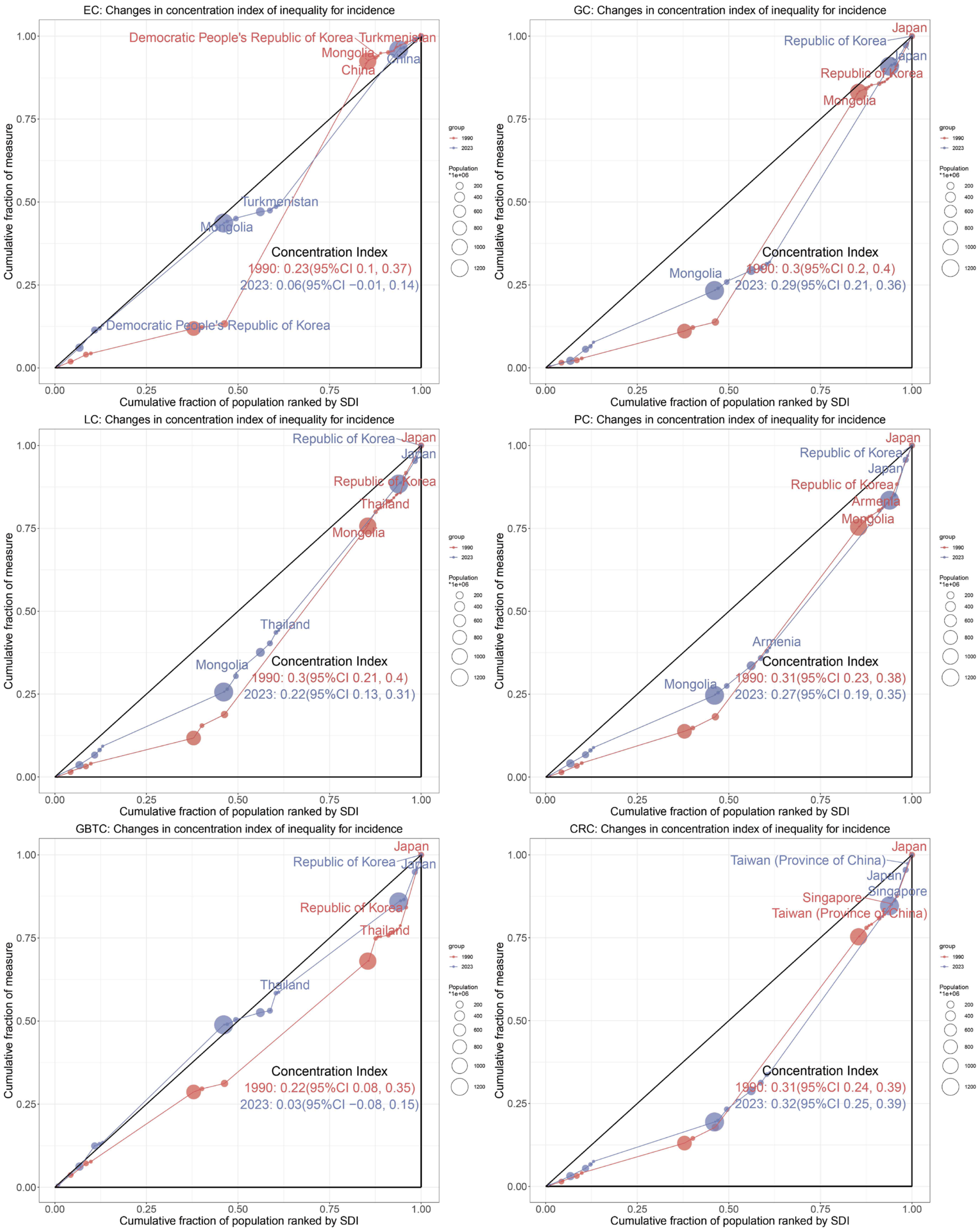

Figure S52: Changes in Relative Inequality (Concentration Index) for age-standardized incidence rates of six major digestive system cancers across the SDI spectrum, 1990-2023. Concentration curves illustrating relative inequality in age-standardized rates for six major digestive system cancers across the SDI spectrum in 1990 (red line/points) and 2023 (blue line/points). Labels indicate the three countries or territories with the highest age-standardized rates in 1990 and 2023, respectively. EC, esophageal cancer; GC, gastric cancer; LC, liver cancer; PC, pancreatic cancer; GBTC, gallbladder and biliary tract cancer; CRC, colorectal cancer.

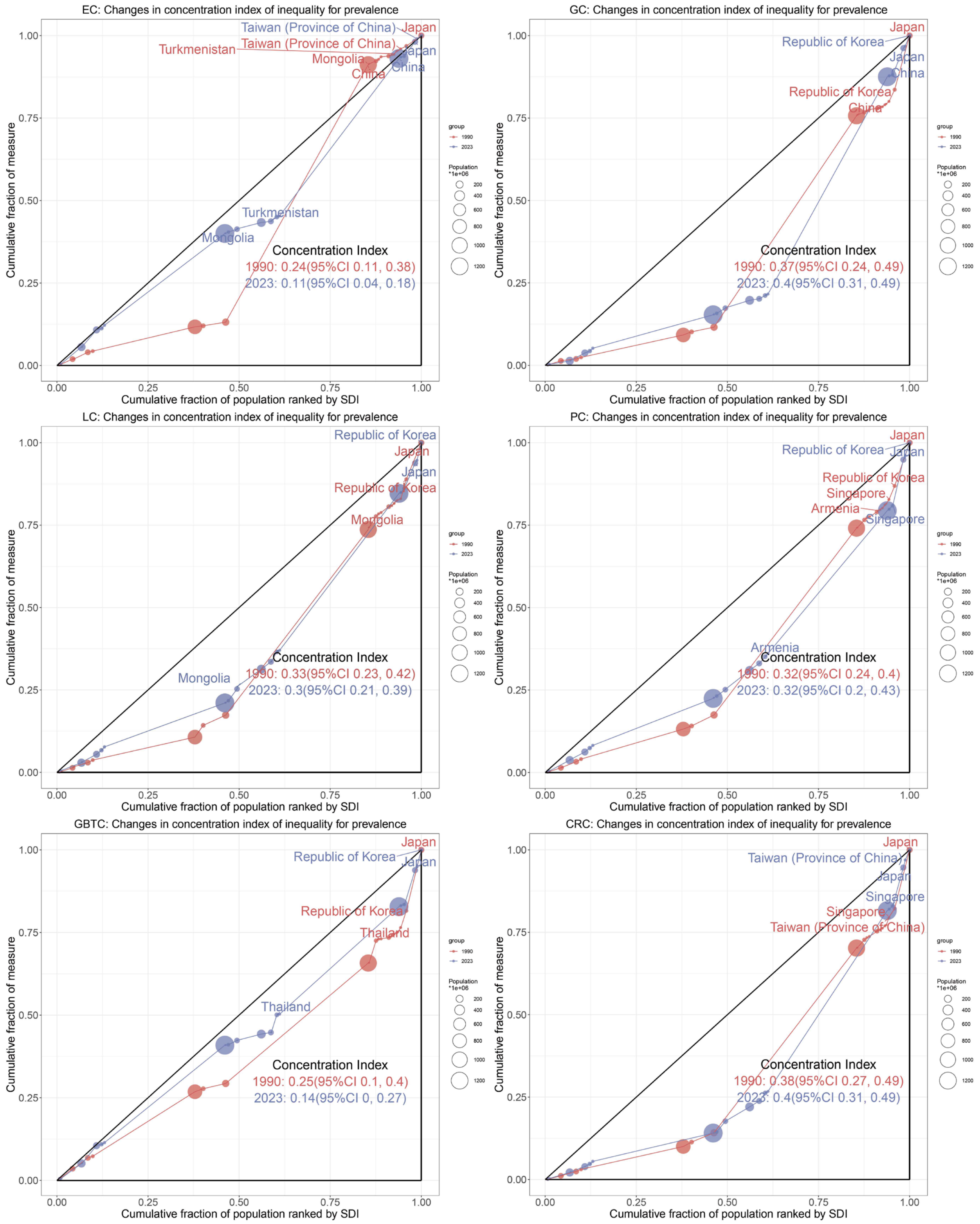

Figure S53: Changes in Relative Inequality (Concentration Index) for age-standardized prevalence rates of six major digestive system cancers across the SDI spectrum, 1990-2023. Concentration curves illustrating relative inequality in age-standardized rates for six major digestive system cancers across the SDI spectrum in 1990 (red line/points) and 2023 (blue line/points). Labels indicate the three countries or territories with the highest age-standardized rates in 1990 and 2023, respectively. EC, esophageal cancer; GC, gastric cancer; LC, liver cancer; PC, pancreatic cancer; GBTC, gallbladder and biliary tract cancer; CRC, colorectal cancer.

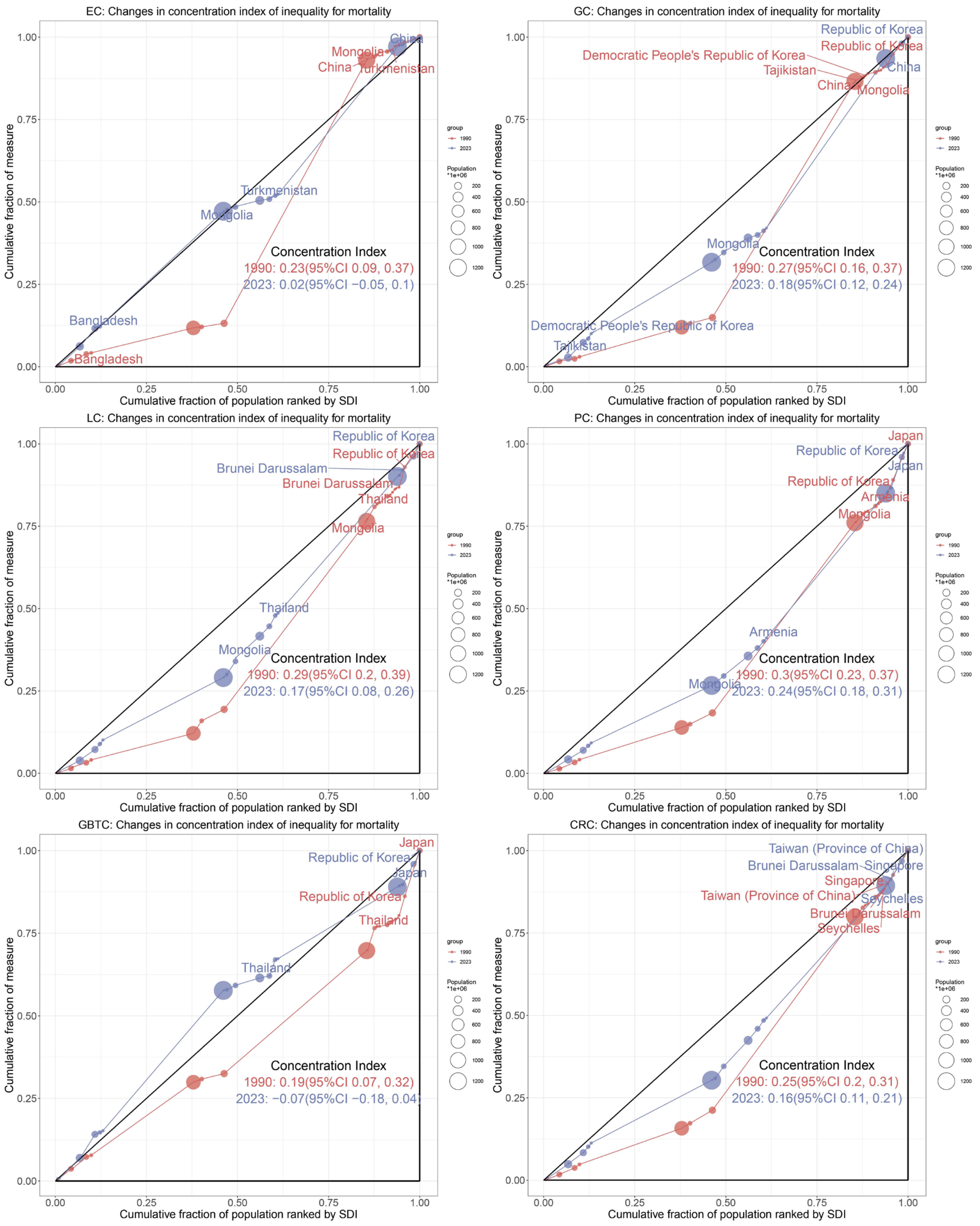

Figure S54: Changes in Relative Inequality (Concentration Index) for age-standardized mortality rates of six major digestive system cancers across the SDI spectrum, 1990-2023. Concentration curves illustrating relative inequality in age-standardized rates for six major digestive system cancers across the SDI spectrum in 1990 (red line/points) and 2023 (blue line/points). Labels indicate the three countries or territories with the highest age-standardized rates in 1990 and 2023, respectively. EC, esophageal cancer; GC, gastric cancer; LC, liver cancer; PC, pancreatic cancer; GBTC, gallbladder and biliary tract cancer; CRC, colorectal cancer.

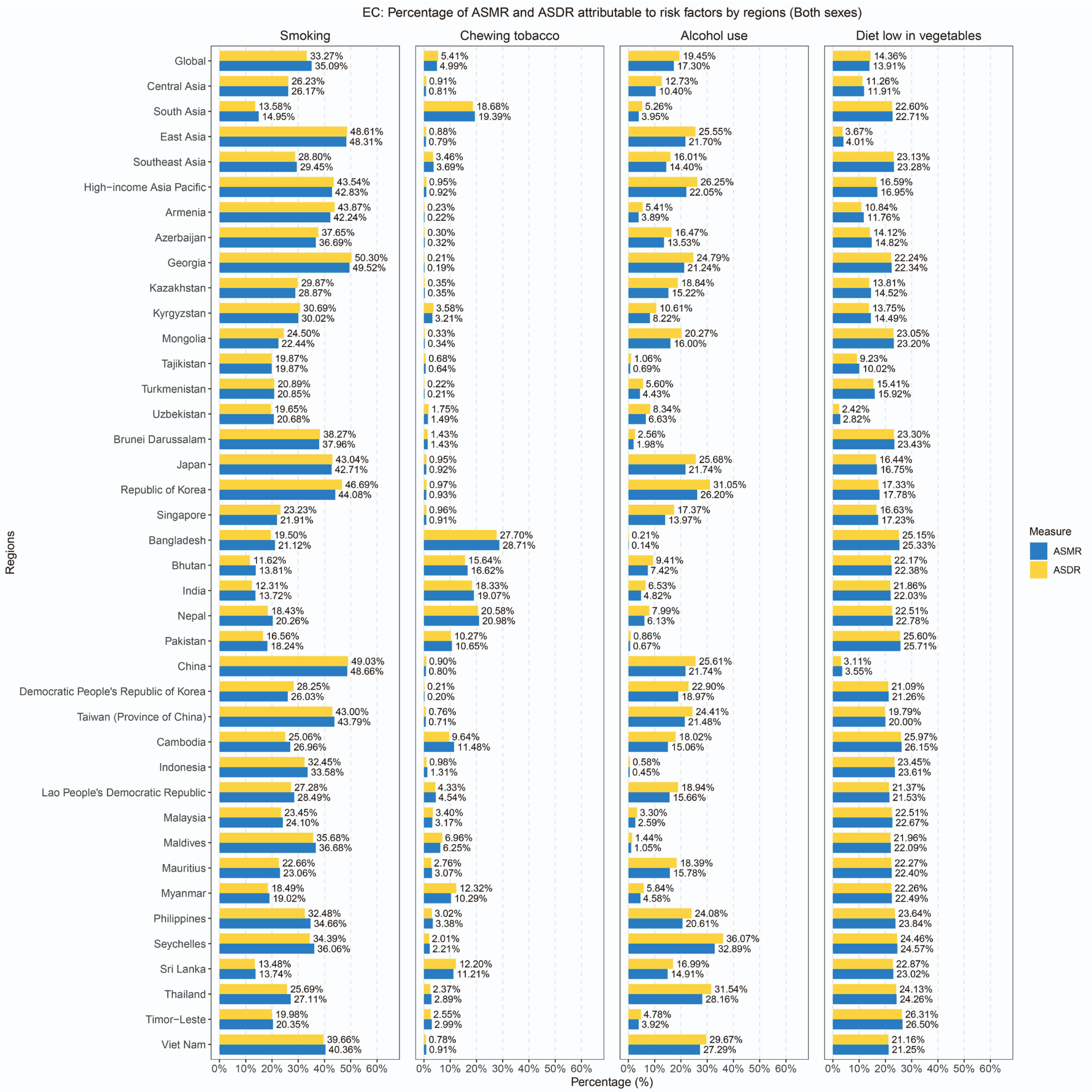

Figure S55: Attributable burden of major risk factors on age-standardized DALY (ASDR) and mortality rates (ASMR) for esophageal cancer (EC) across Asian regions, both sexes combined, 2023. DALY, disability-adjusted life year.

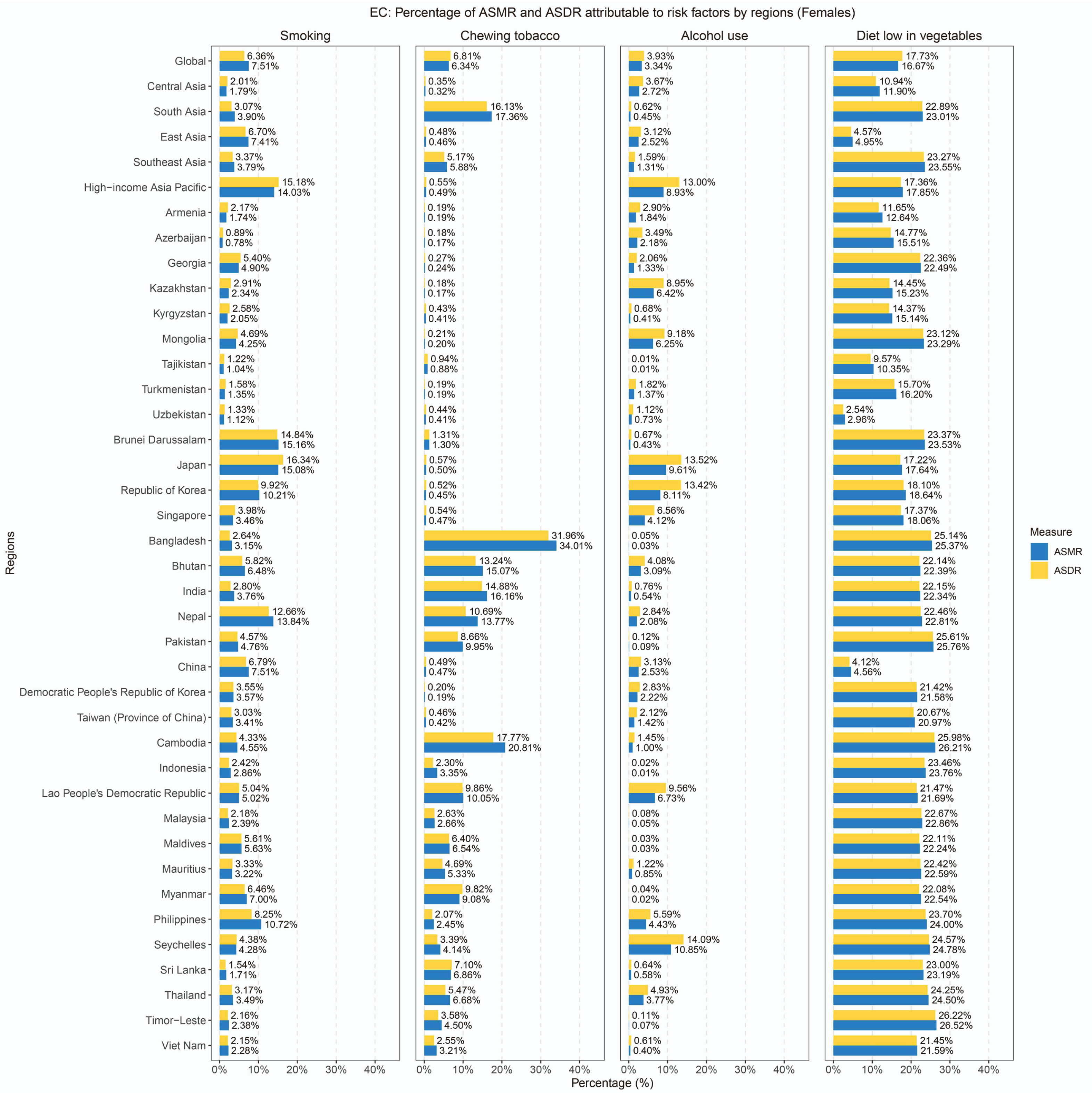

Figure S56: Attributable burden of major risk factors on age-standardized DALY (ASDR) and mortality rates (ASMR) for esophageal cancer (EC) across Asian regions, females, 2023. DALY, disability-adjusted life year.

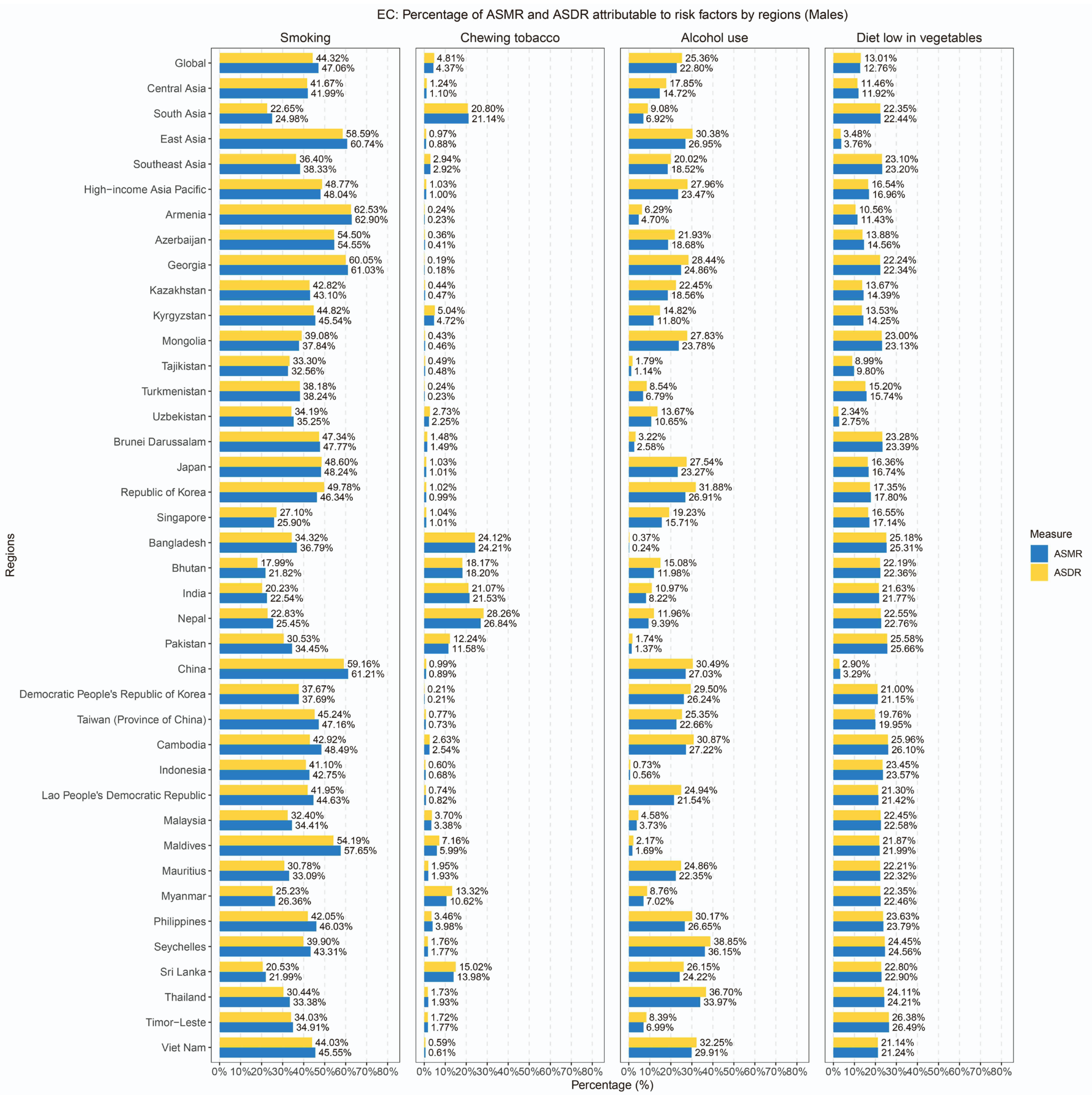

Figure S57: Attributable burden of major risk factors on age-standardized DALY (ASDR) and mortality rates (ASMR) for esophageal cancer (EC) across Asian regions, males, 2023. DALY, disability-adjusted life year.

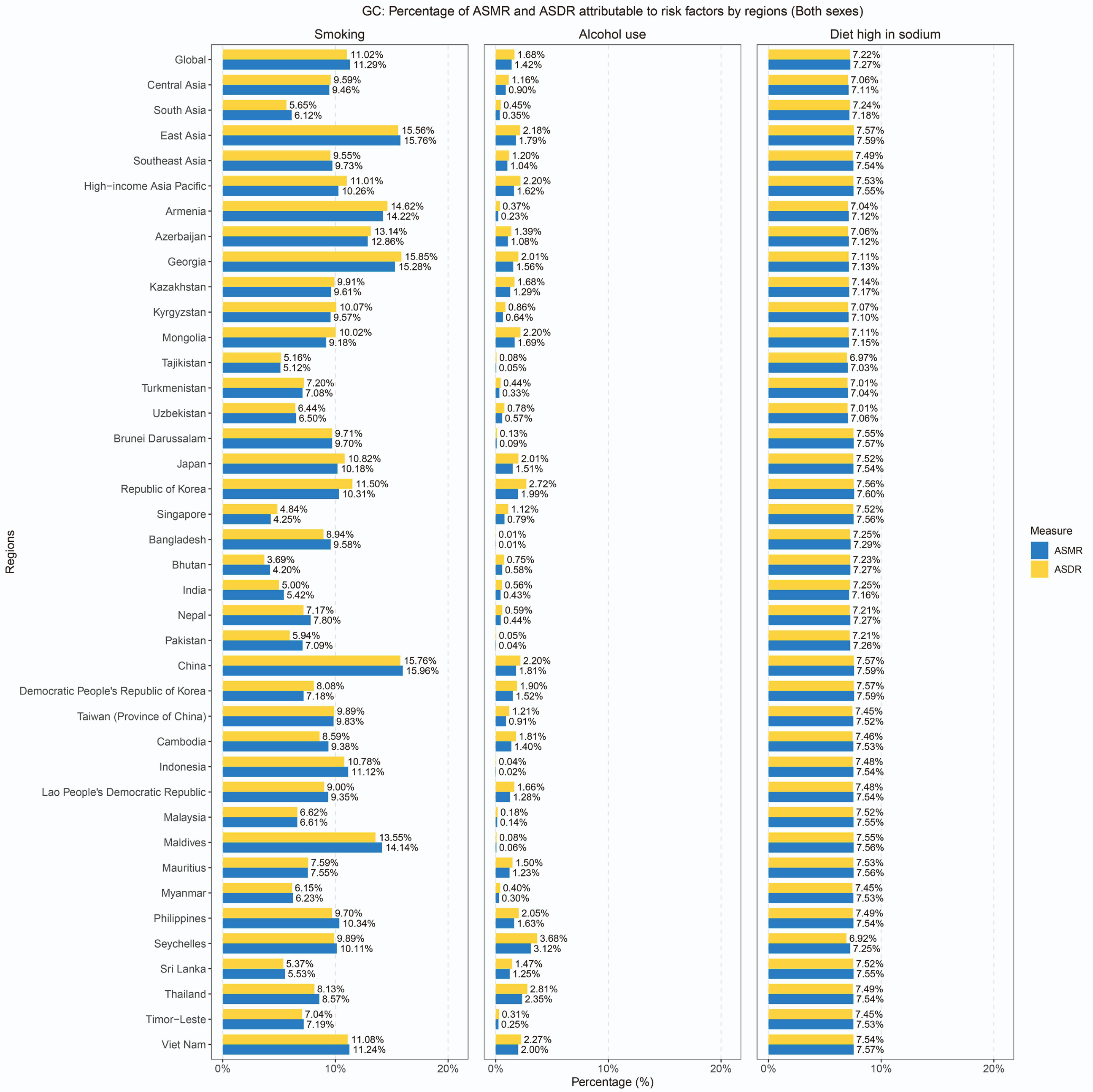

Figure S58: Attributable burden of major risk factors on age-standardized DALY (ASDR) and mortality rates (ASMR) for gastric cancer (GC) across Asian regions, both sexes combined, 2023. DALY, disability-adjusted life year.

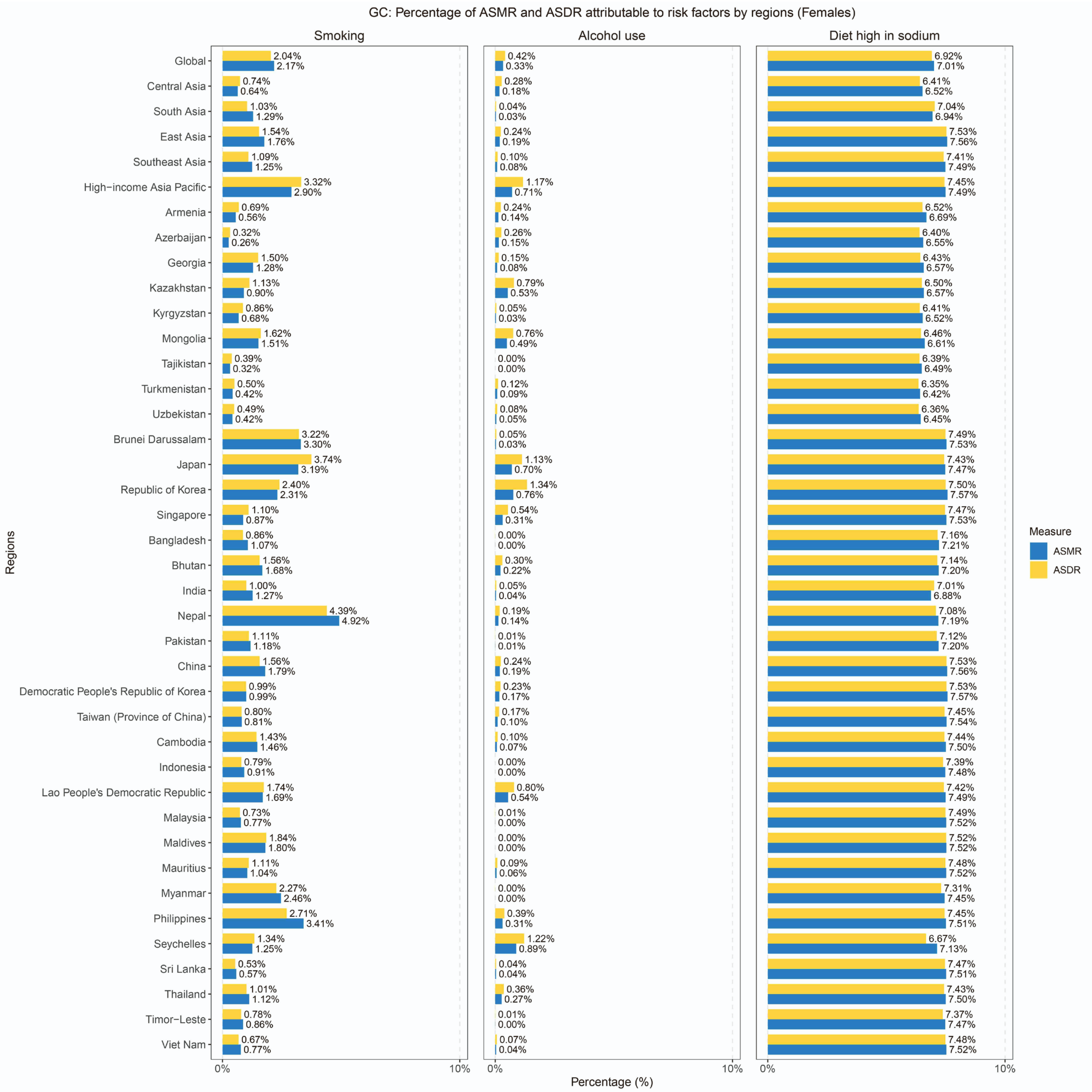

Figure S59: Attributable burden of major risk factors on age-standardized DALY (ASDR) and mortality rates (ASMR) for gastric cancer (GC) across Asian regions, females, 2023. DALY, disability-adjusted life year.

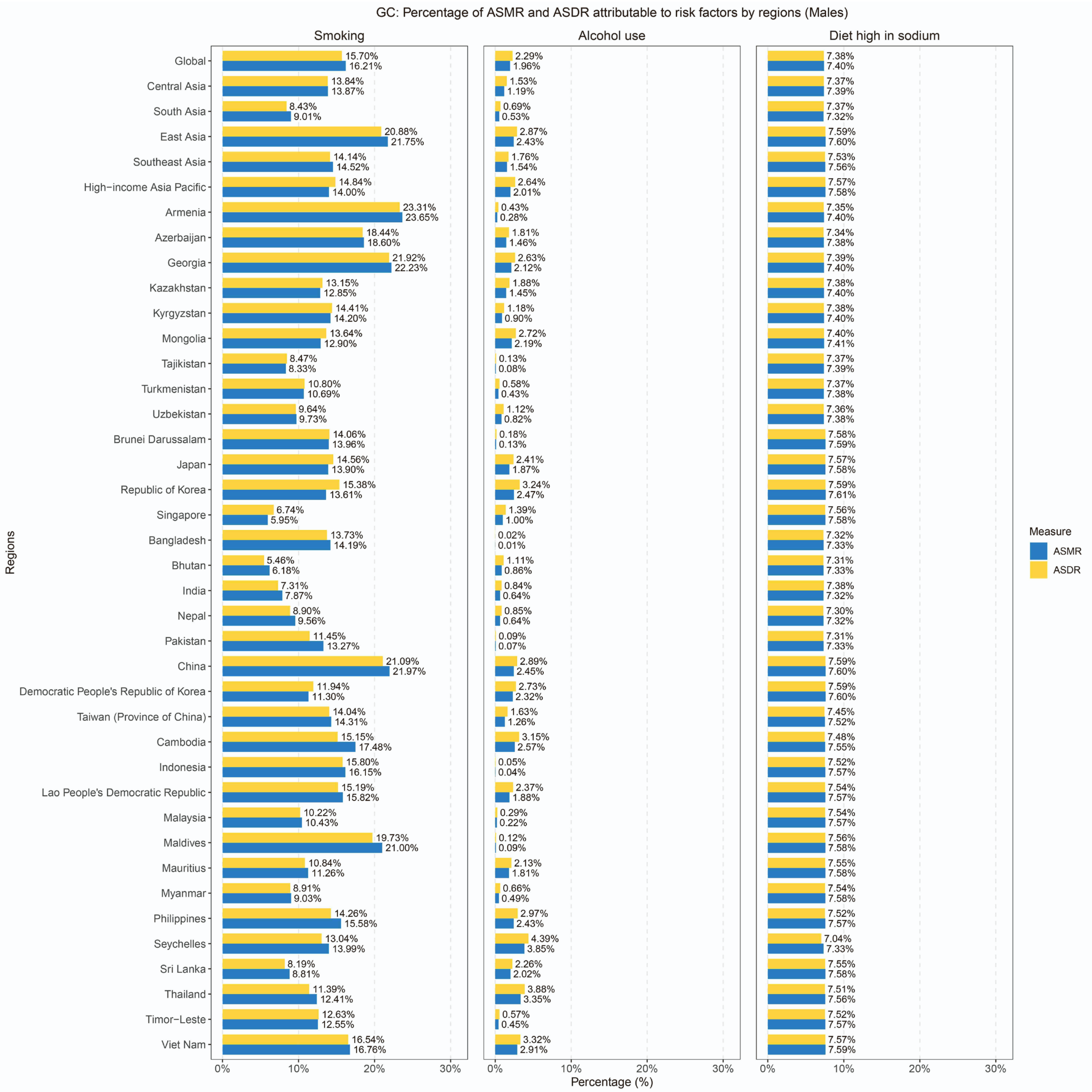

Figure S60: Attributable burden of major risk factors on age-standardized DALY (ASDR) and mortality rates (ASMR) for gastric cancer (GC) across Asian regions, males, 2023. DALY, disability-adjusted life year.

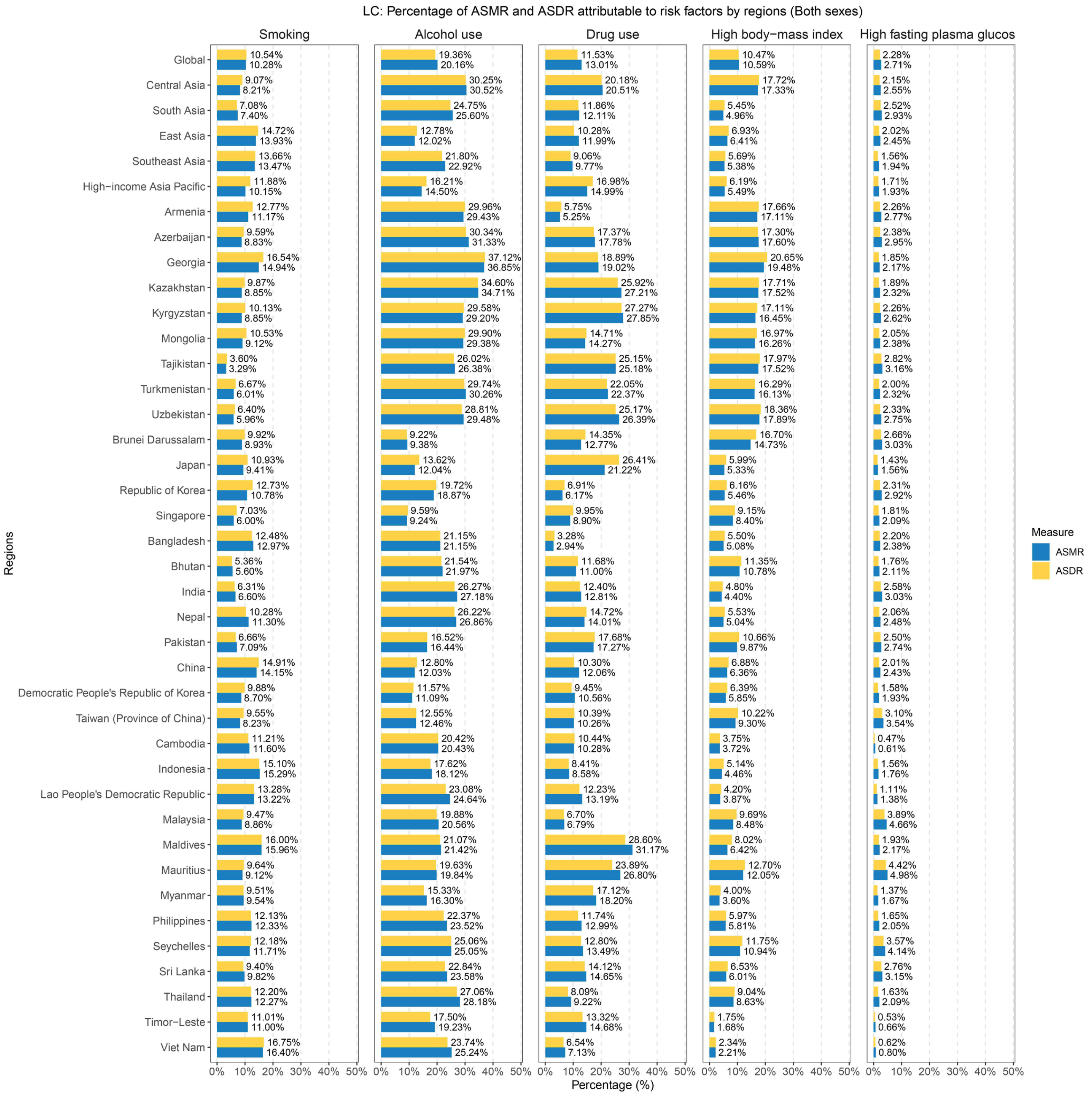

Figure S61: Attributable burden of major risk factors on age-standardized DALY (ASDR) and mortality rates (ASMR) for liver cancer (LC) across Asian regions, both sexes combined, 2023. DALY, disability-adjusted life year.

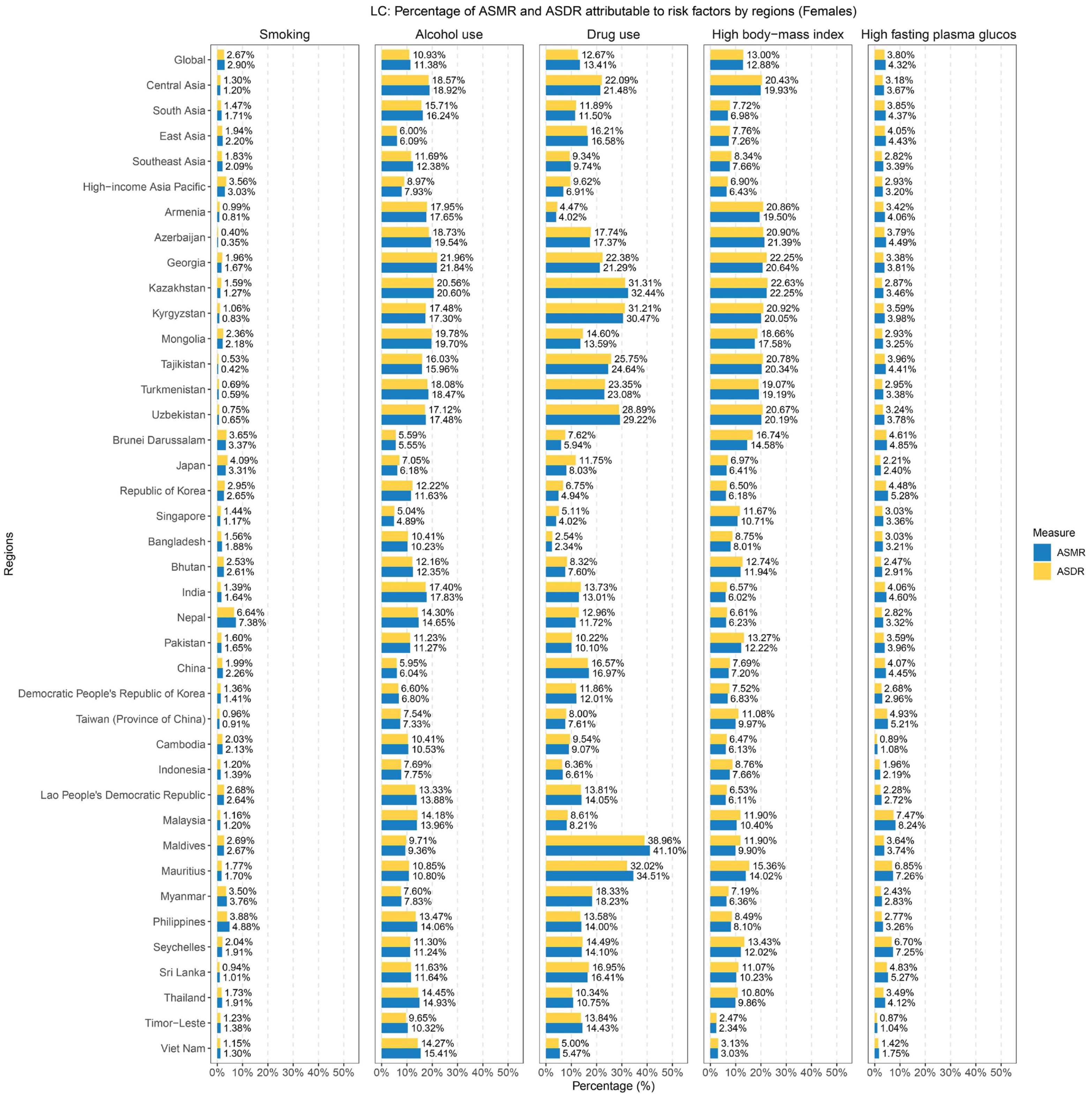

Figure S62: Attributable burden of major risk factors on age-standardized DALY (ASDR) and mortality rates (ASMR) for liver cancer (LC) across Asian regions, females, 2023. DALY, disability-adjusted life year.

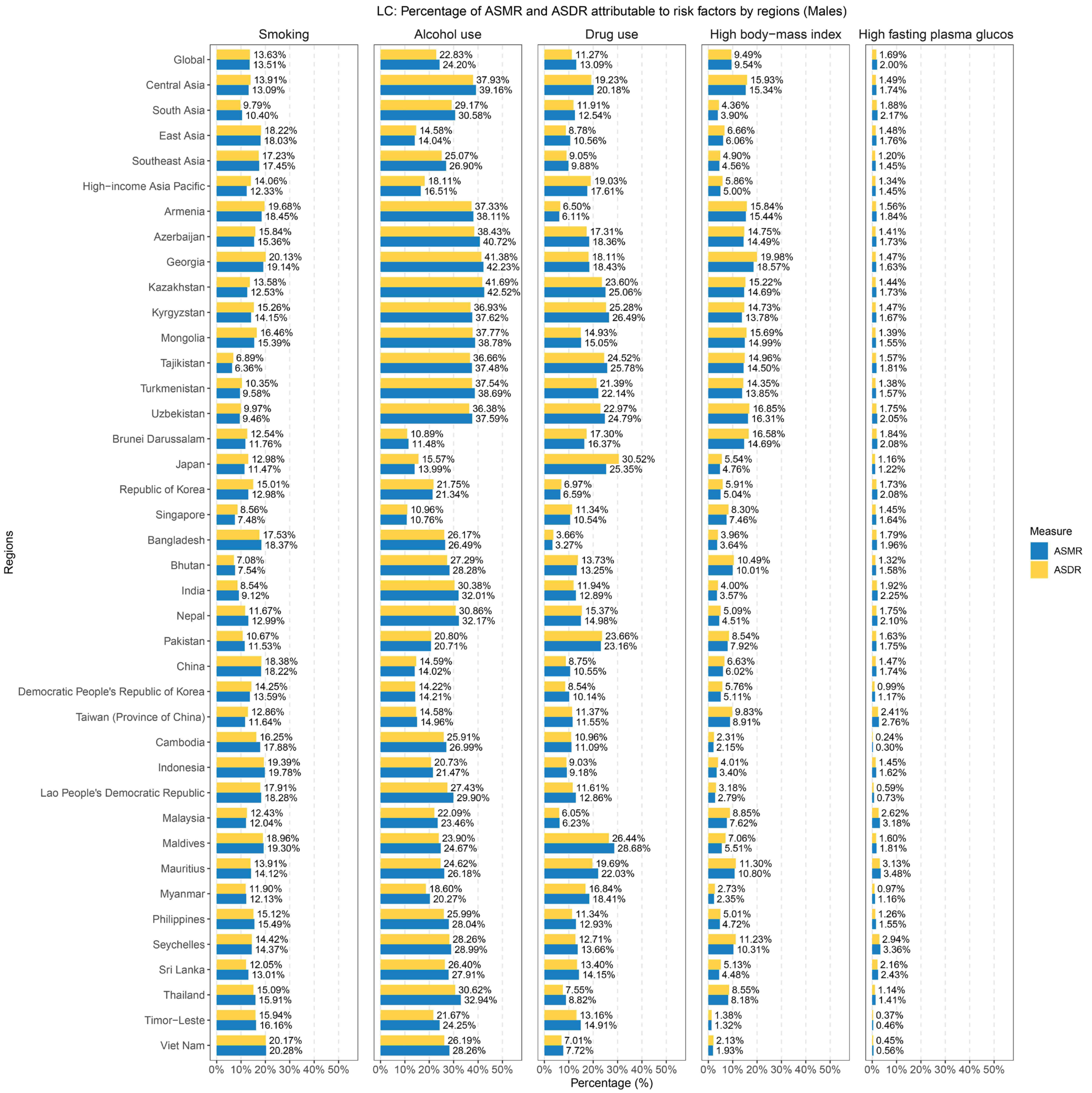

Figure S63: Attributable burden of major risk factors on age-standardized DALY (ASDR) and mortality rates (ASMR) for liver cancer (LC) across Asian regions, males, 2023. DALY, disability-adjusted life year.

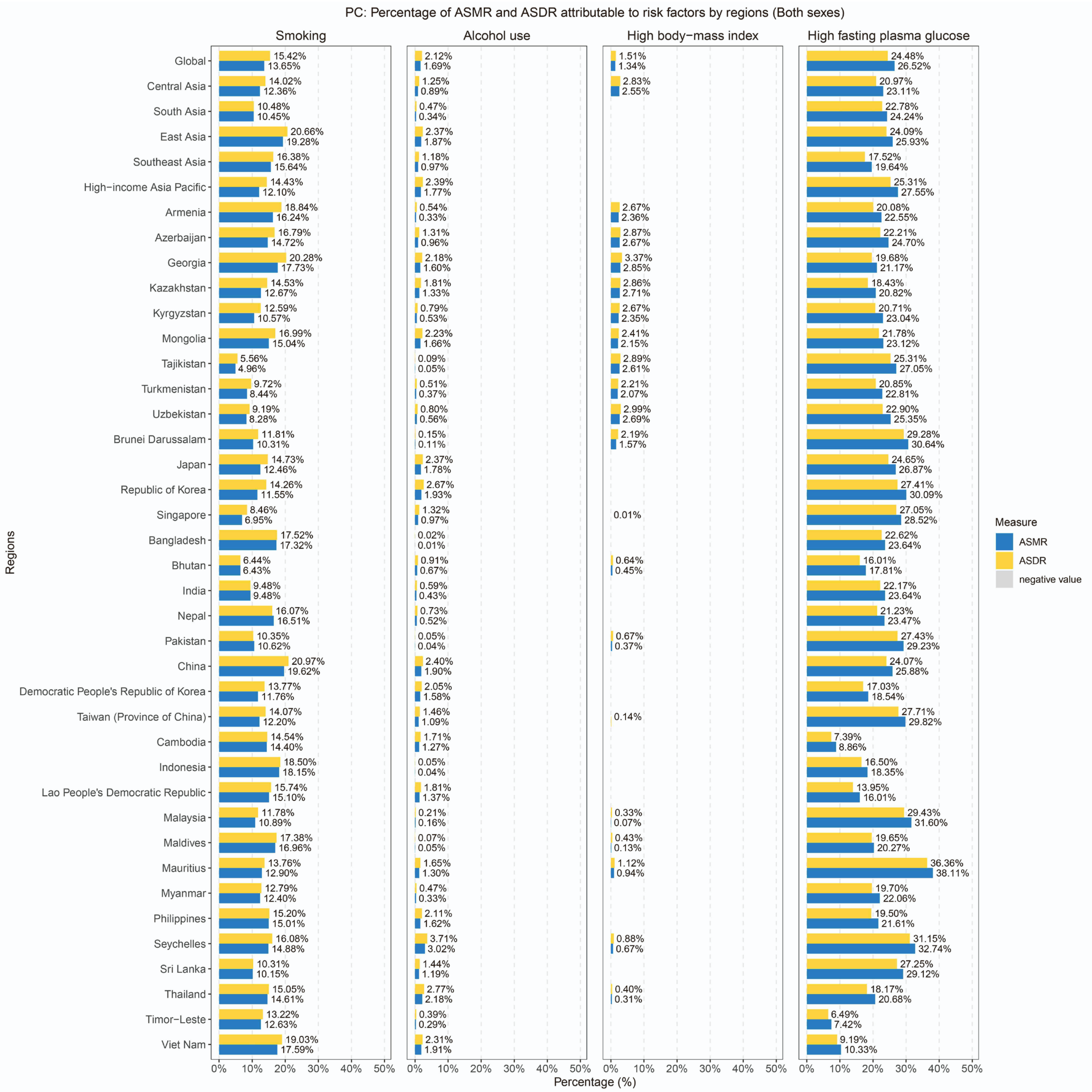

Figure S64: Attributable burden of major risk factors on age-standardized DALY (ASDR) and mortality rates (ASMR) for pancreatic cancer (PC) across Asian regions, both sexes combined, 2023. DALY, disability-adjusted life year.

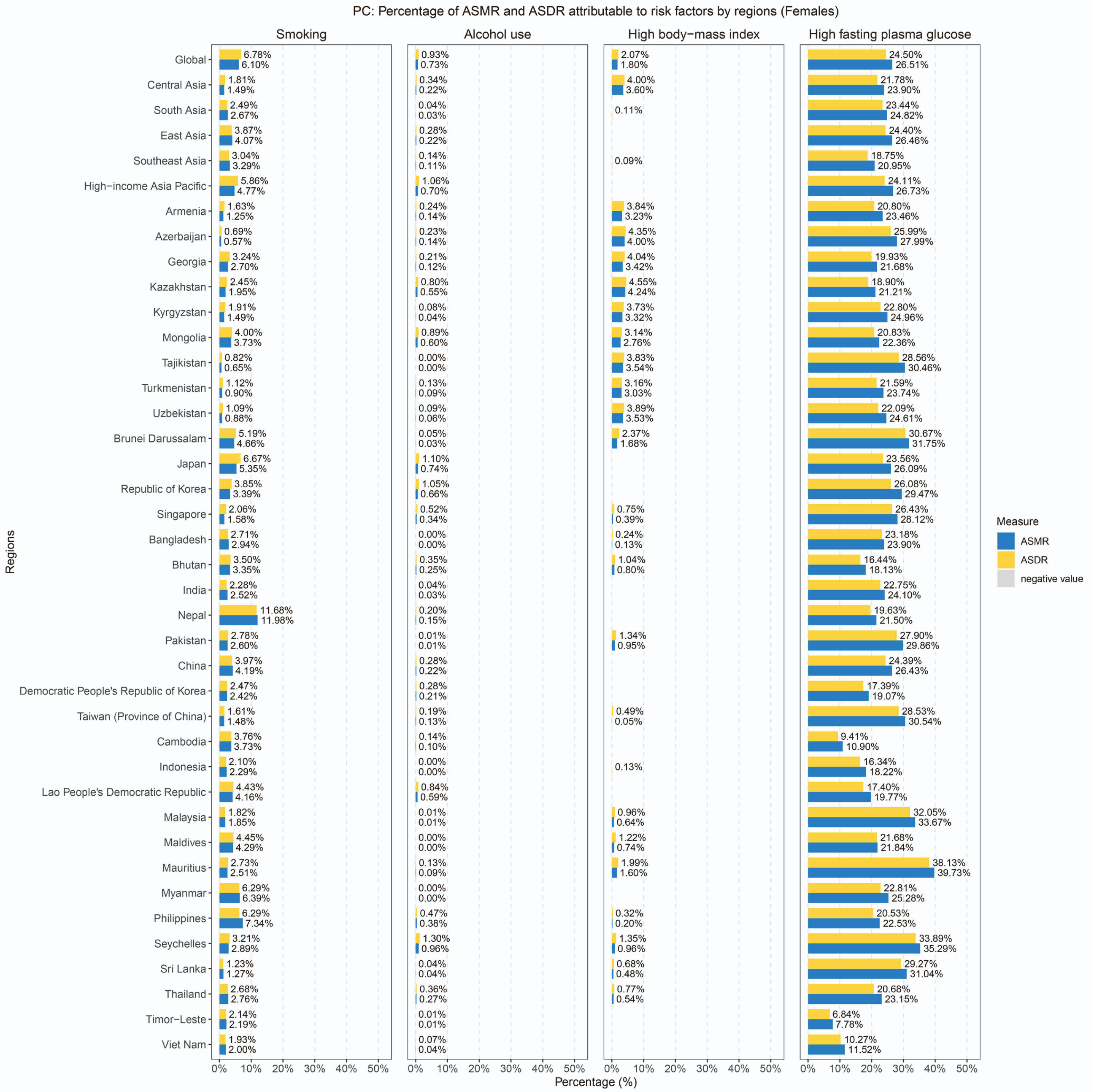

Figure S65: Attributable burden of major risk factors on age-standardized DALY (ASDR) and mortality rates (ASMR) for pancreatic cancer (PC) across Asian regions, females, 2023. DALY, disability-adjusted life year.

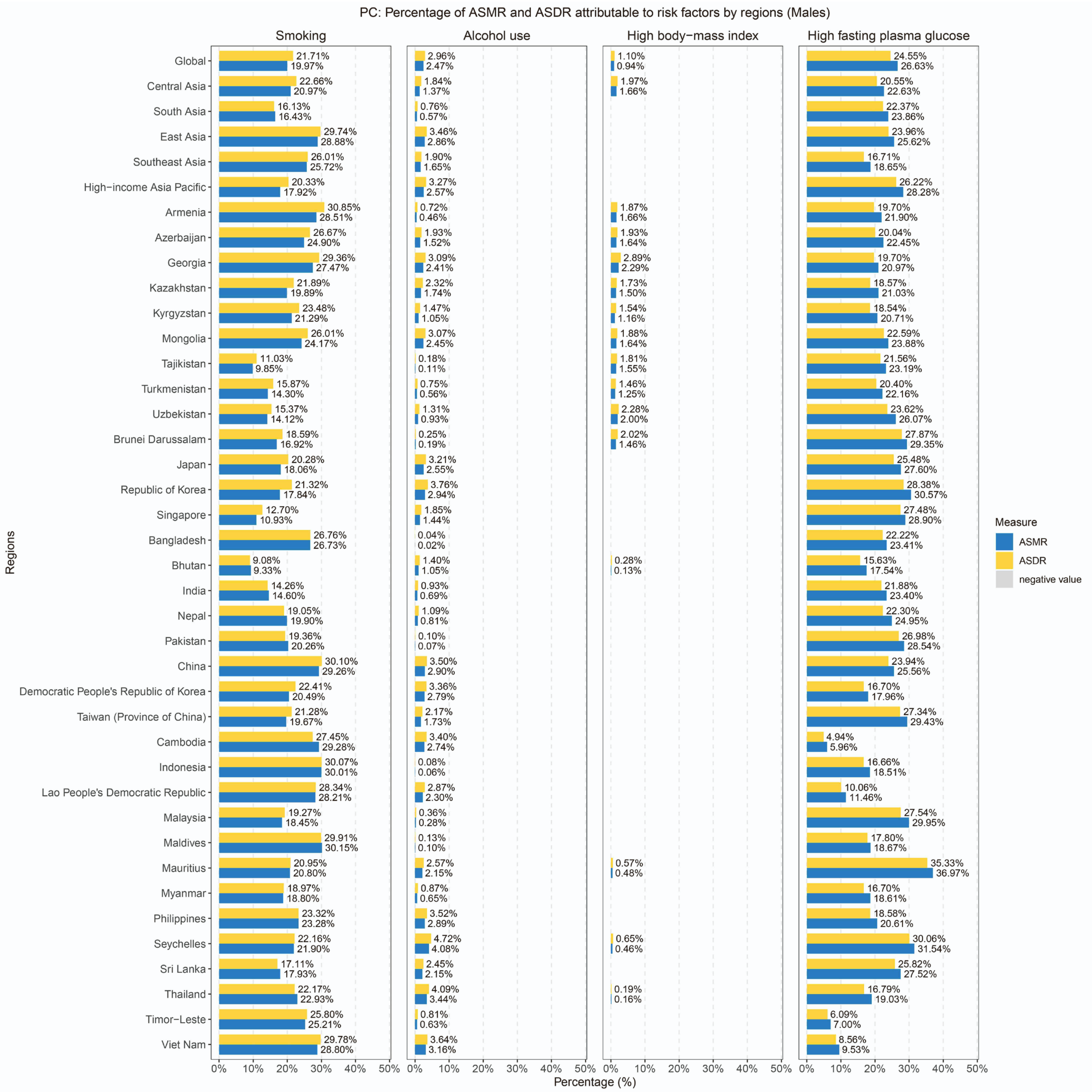

Figure S66: Attributable burden of major risk factors on age-standardized DALY (ASDR) and mortality rates (ASMR) for pancreatic cancer (PC) across Asian regions, males, 2023. DALY, disability-adjusted life year.

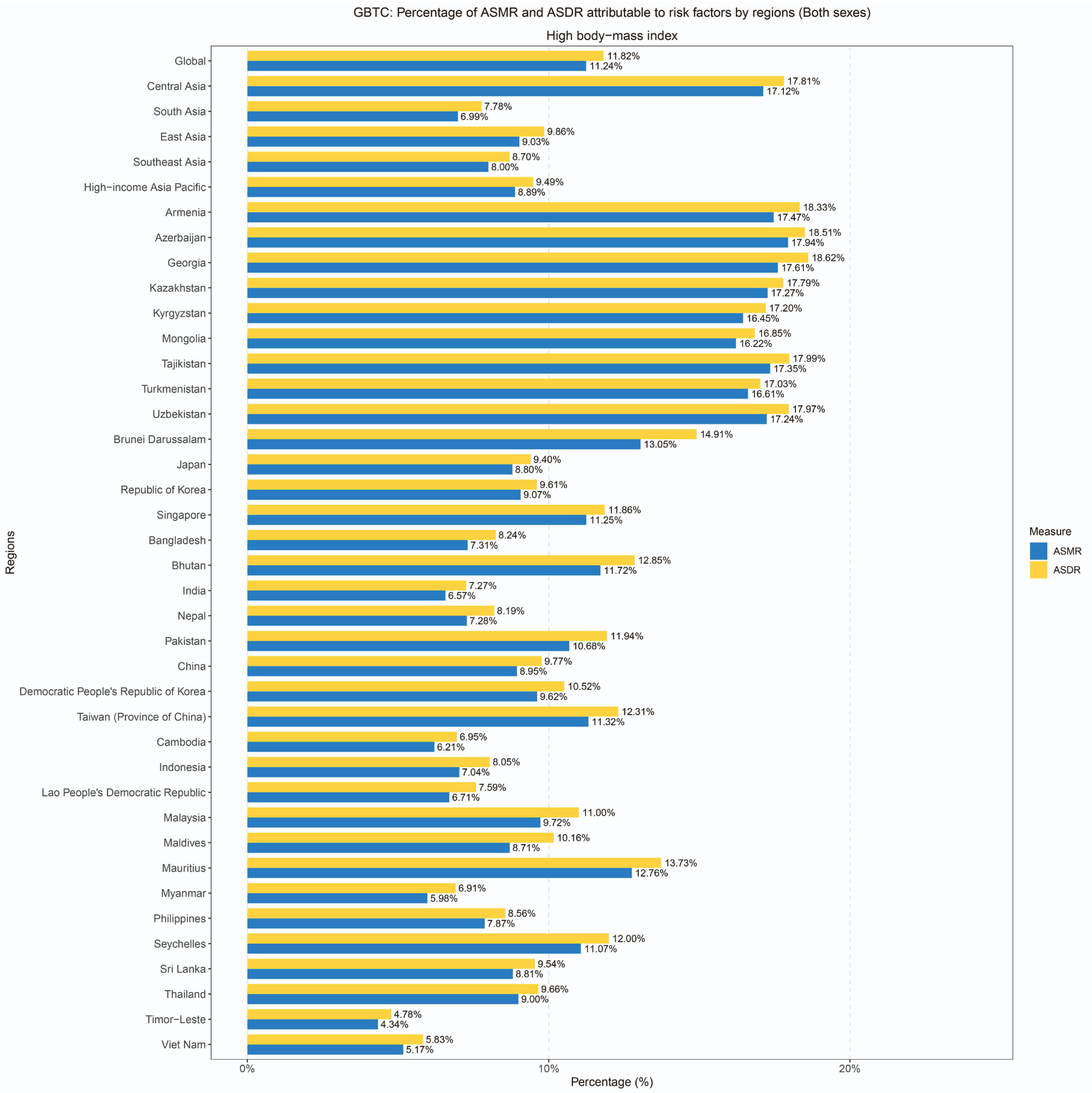

Figure S67: Attributable burden of major risk factors on age-standardized DALY (ASDR) and mortality rates (ASMR) for gallbladder and biliary tract cancer (GBTC) across Asian regions, both sexes combined, 2023. DALY, disability-adjusted life year.

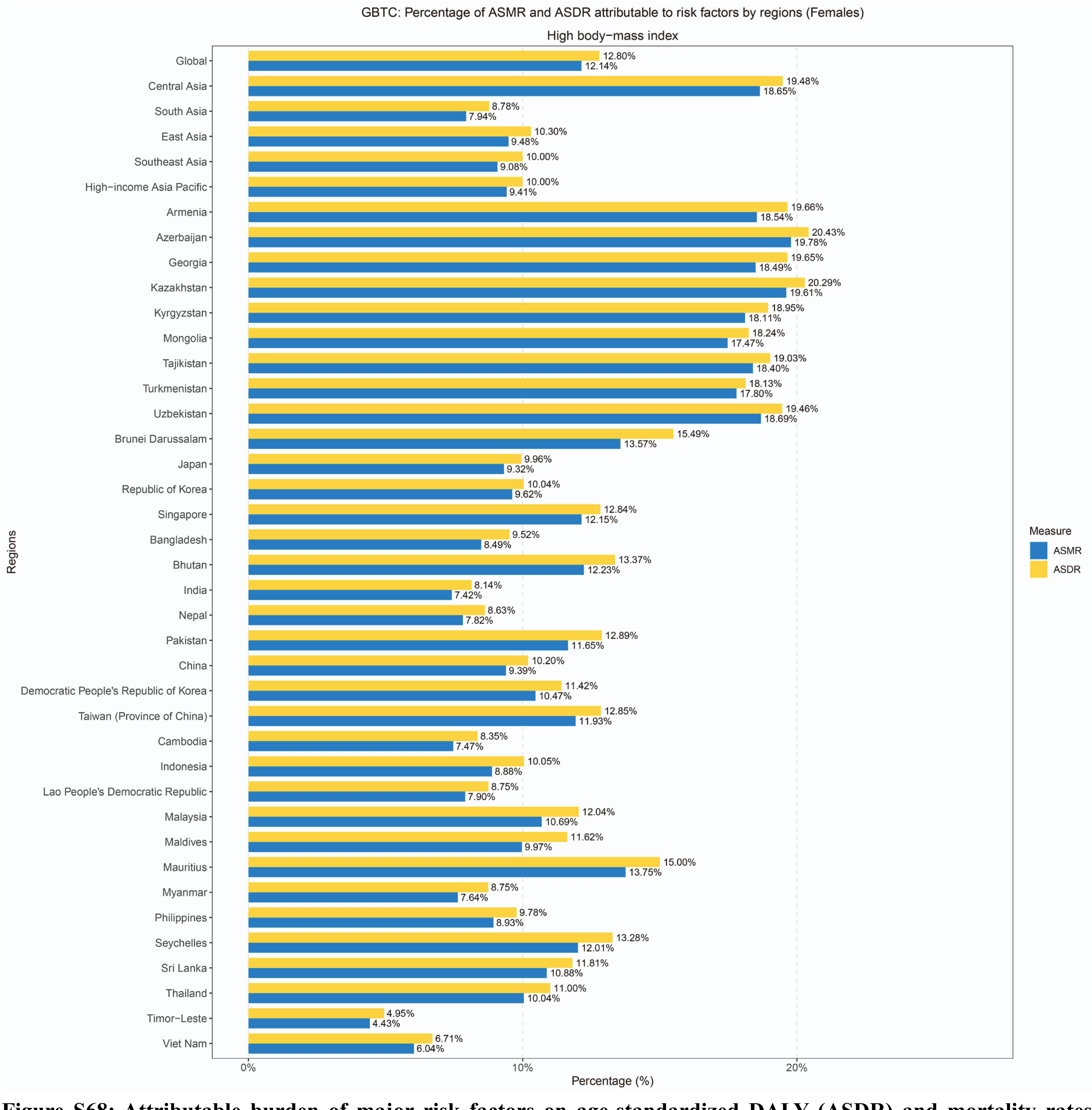

Figure S68: Attributable burden of major risk factors on age-standardized DALY (ASDR) and mortality rates (ASMR) for gallbladder and biliary tract cancer (GBTC) across Asian regions, females, 2023. DALY, disability-adjusted life year.

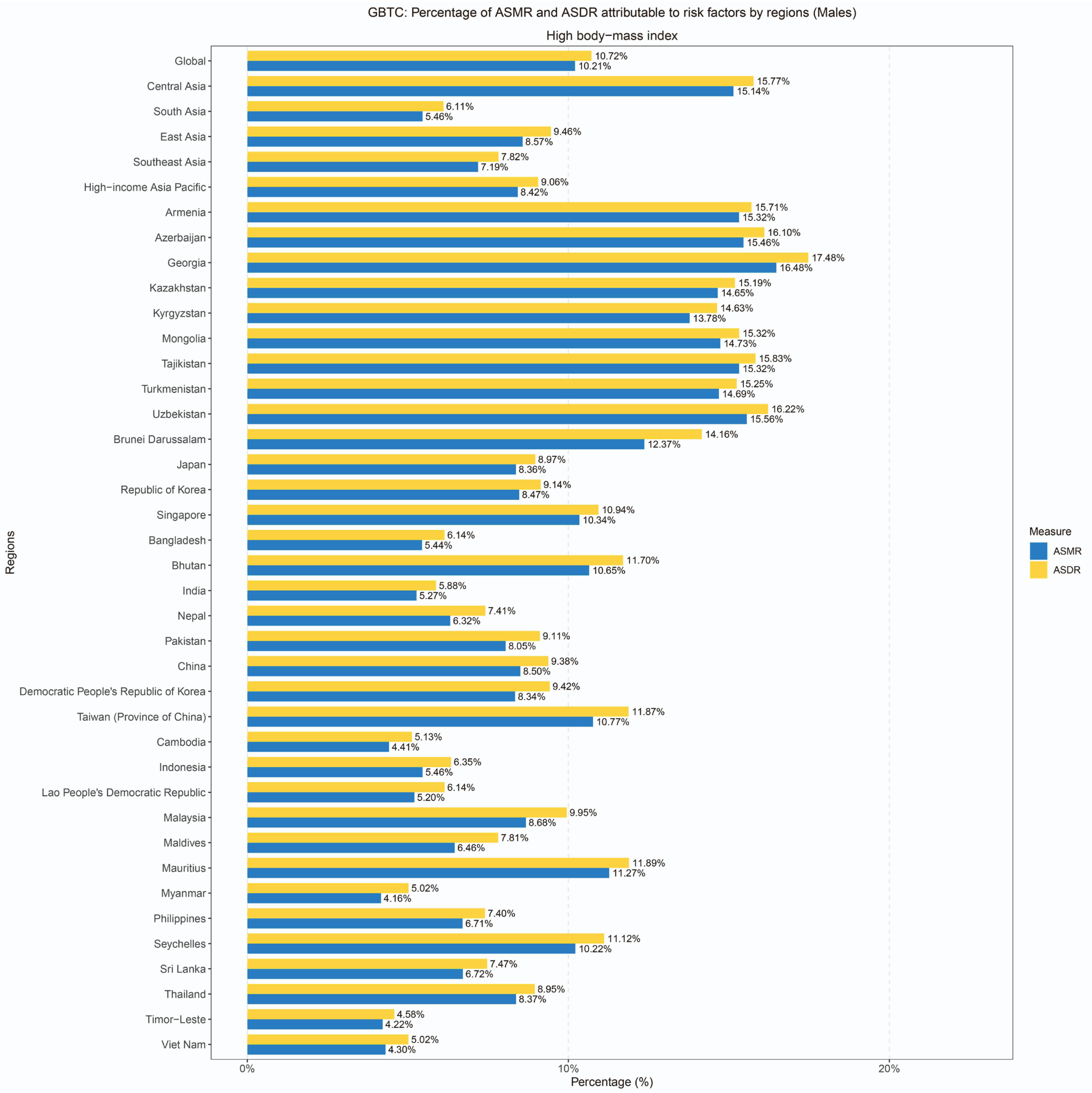

Figure S69: Attributable burden of major risk factors on age-standardized DALY (ASDR) and mortality rates (ASMR) for gallbladder and biliary tract cancer (GBTC) across Asian regions, males, 2023. DALY, disability-adjusted life year.

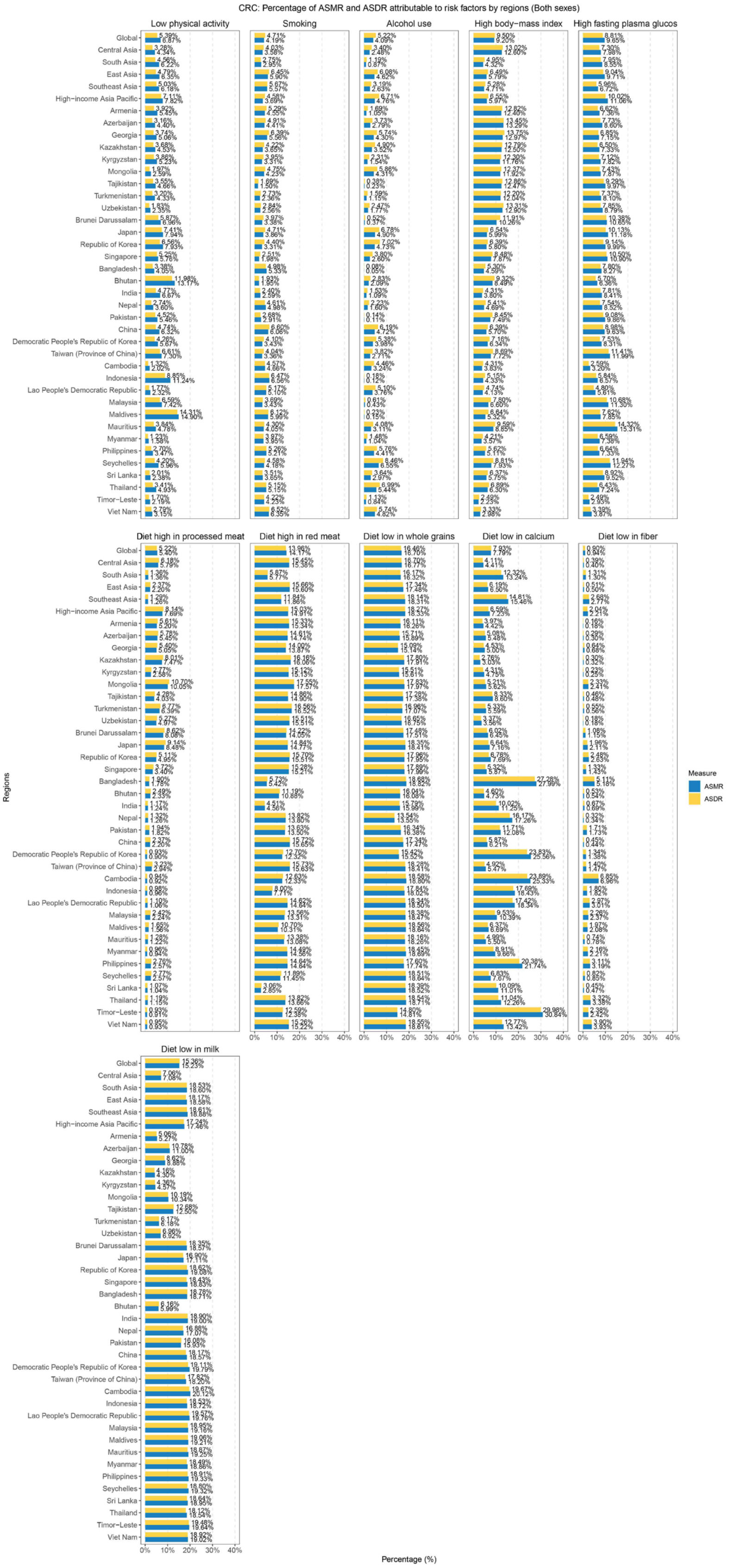

Figure S70: Attributable burden of major risk factors on age-standardized DALY (ASDR) and mortality rates (ASMR) for colorectal cancer (CRC) across Asian regions, both sexes combined, 2023. DALY, disability-adjusted life year.

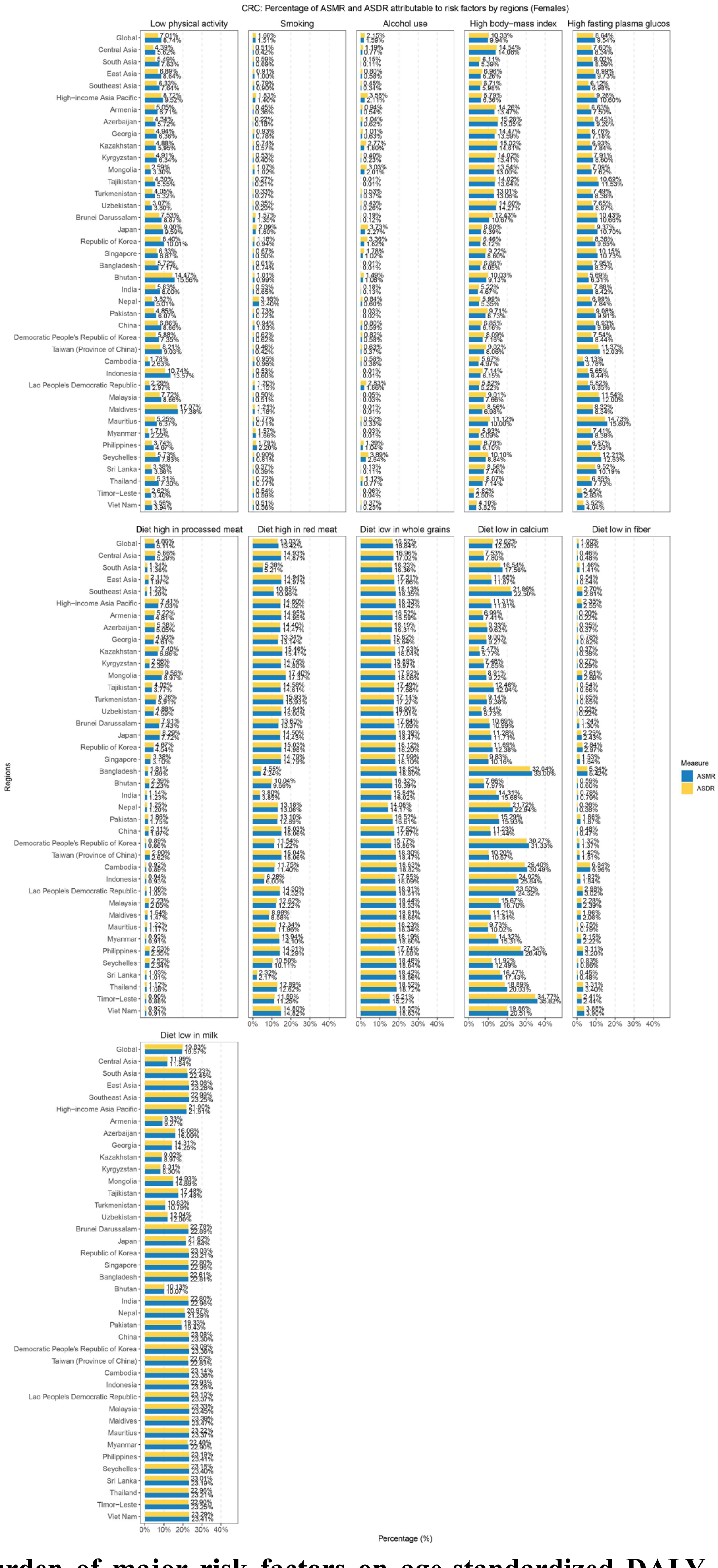

Figure S71: Attributable burden of major risk factors on age-standardized DALY (ASDR) and mortality rates (ASMR) for colorectal cancer (CRC) across Asian regions, females, 2023. DALY, disability-adjusted life year.

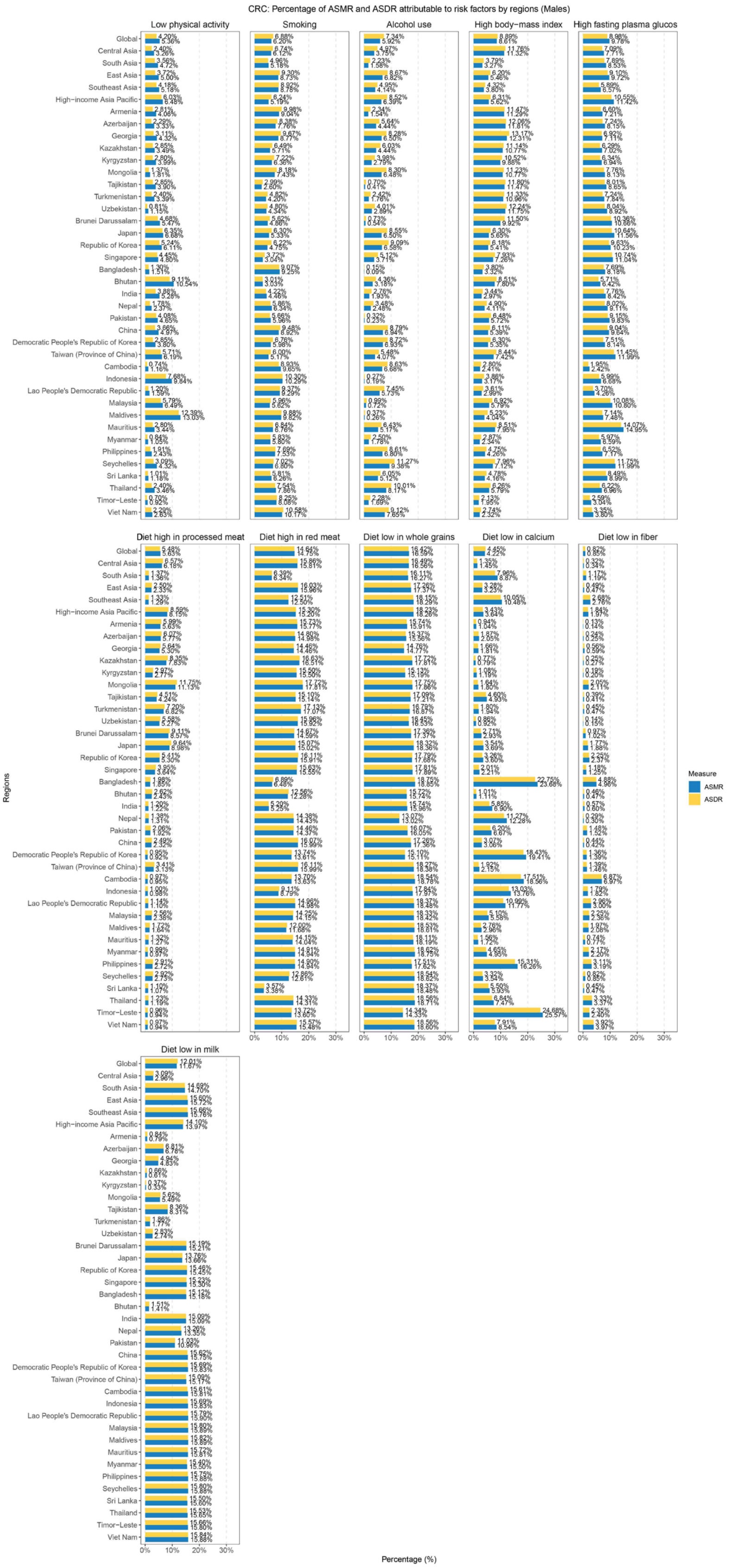

Figure S72: Attributable burden of major risk factors on age-standardized DALY (ASDR) and mortality rates (ASMR) for colorectal cancer (CRC) across Asian regions, males, 2023. DALY, disability-adjusted life year.

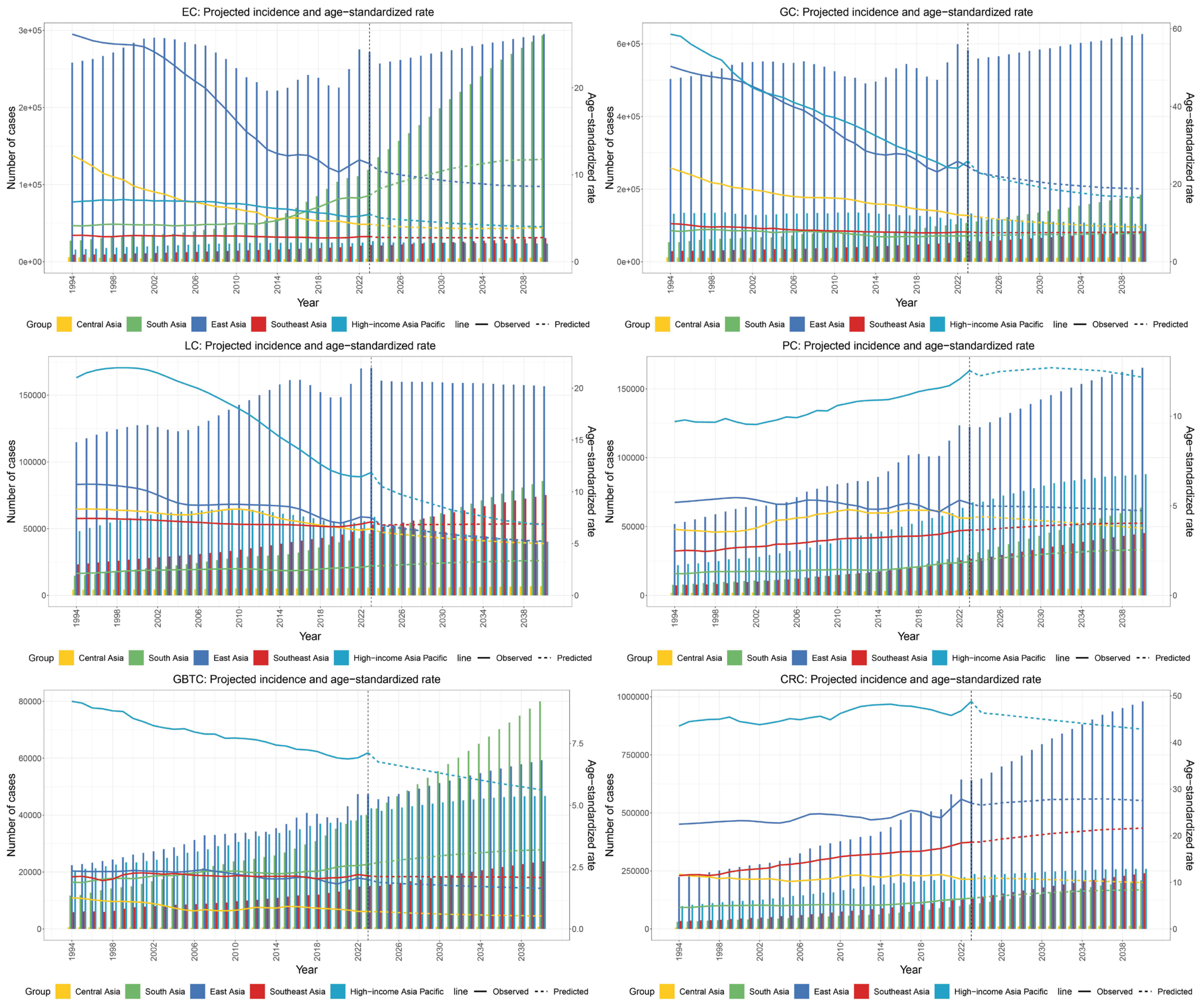

Figure S73: Nordpred projections of absolute incidence and age-standardized rates for six major digestive system cancers in Asian regions, 2024-2040. EC, esophageal cancer; GC, gastric cancer; LC, liver cancer; PC, pancreatic cancer; GBTC, gallbladder and biliary tract cancer; CRC, colorectal cancer.

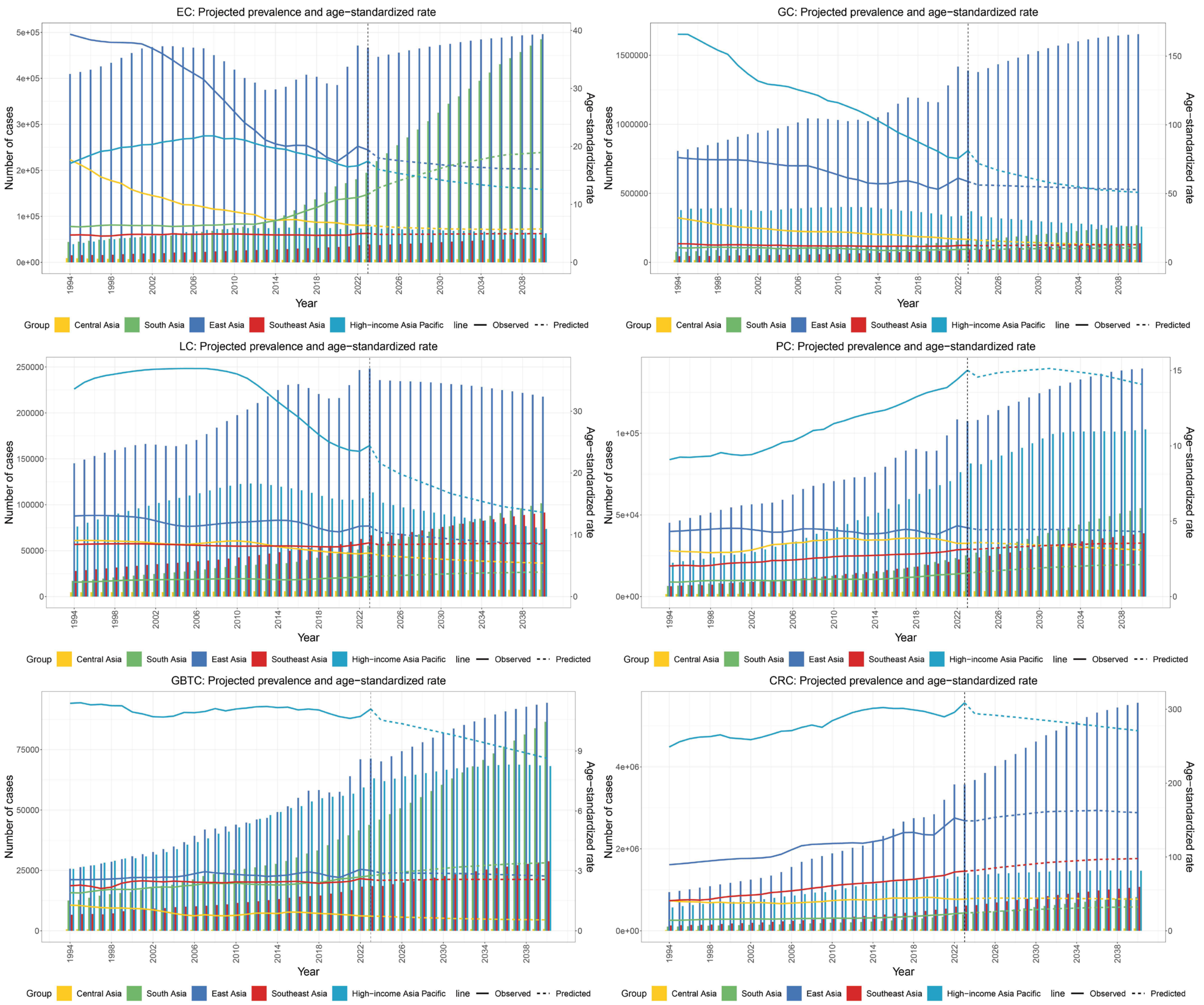

Figure S74: Nordpred projections of absolute prevalence and age-standardized rates for six major digestive system cancers in Asian regions, 2024-2040. EC, esophageal cancer; GC, gastric cancer; LC, liver cancer; PC, pancreatic cancer; GBTC, gallbladder and biliary tract cancer; CRC, colorectal cancer.

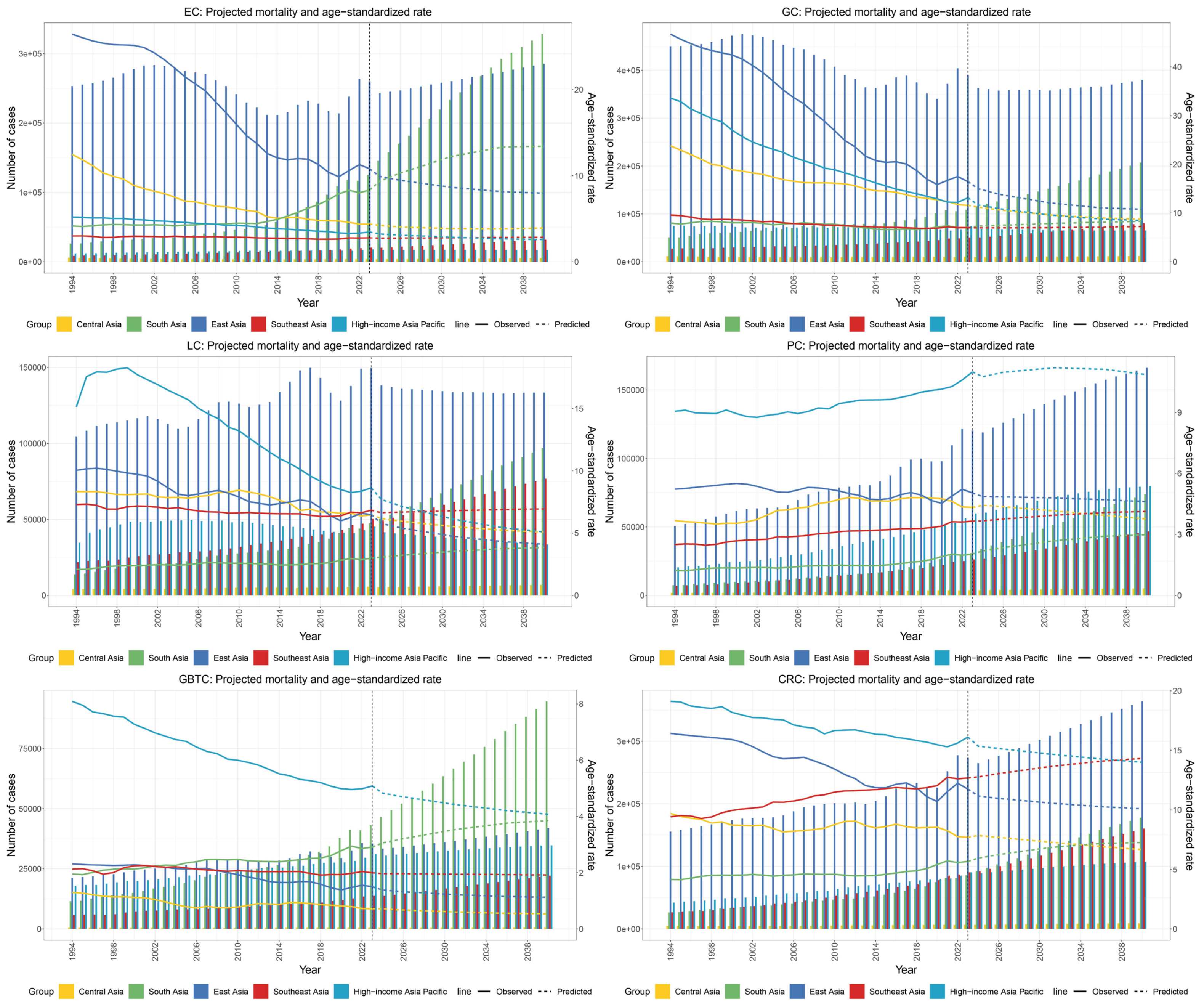

Figure S75: Nordpred projections of absolute mortality and age-standardized rates for six major digestive system cancers in Asian regions, 2024-2040. EC, esophageal cancer; GC, gastric cancer; LC, liver cancer; PC, pancreatic cancer; GBTC, gallbladder and biliary tract cancer; CRC, colorectal cancer.

Table S3: List of International Classification of Diseases (ICD) codes mapped to six major digestive system cancers, related to Methods

Cause	ICD10
Gastric cancer	C16-C16.9, D00.2, D13.1, D37.1
Colon and rectum cancer	C18-C21.9, D01.0-D01.3, D12-D12.9, D37.3-D37.5
Liver cancer	C22-C22.8, D13.4
Gallbladder and biliary tract cancer	C23-C24.9, D13.5
Pancreatic cancer	C25-C25.9, D13.6-D13.7
Esophageal cancer	C15-C15.9, D00.1, D13.0
